# Genome and transcriptome characterization of the glycoengineered *Nicotiana benthamiana* line ΔXT/FT

**DOI:** 10.1186/s12864-019-5960-2

**Published:** 2019-07-19

**Authors:** Matteo Schiavinato, Richard Strasser, Lukas Mach, Juliane C. Dohm, Heinz Himmelbauer

**Affiliations:** 1 Department of Biotechnology, University of Natural Resources and Life Sciences (BOKU), Muthgasse 18, 1190 Vienna, Austria; 2 Department of Applied Genetics and Cell Biology, University of Natural Resources and Life Sciences (BOKU), Muthgasse 18, 1190 Vienna, Austria

**Keywords:** *Nicotiana benthamiana*, Genome, Gene prediction, Transgene, Intraspecific variation, Accession history

## Abstract

**Background:**

The allotetraploid tobacco species *Nicotiana benthamiana* native to Australia has become a popular host for recombinant protein production. Although its usage grows every year, little is known on this plant’s genomic and transcriptomic features. Most *N. benthamiana* accessions currently used in research lack proper documentation of their breeding history and provenance. One of these, the glycoengineered *N. benthamiana* line ΔXT/FT is increasingly used for the production of biopharmaceutical proteins.

**Results:**

Based on an existing draft assembly of the *N. benthamiana* genome we predict 50,516 protein –encoding genes (62,216 transcripts) supported by expression data derived from 2.35 billion mRNA-seq reads. Using single-copy core genes we show high completeness of the predicted gene set. We functionally annotate more than two thirds of the gene set through sequence homology to genes from other *Nicotiana* species. We demonstrate that the expression profiles from leaf tissue of ΔXT/FT and its wild type progenitor only show minimal differences. We identify the transgene insertion sites in ΔXT/FT and show that one of the transgenes was inserted inside another predicted gene that most likely lost its function upon insertion. Based on publicly available mRNA-seq data, we confirm that the *N. benthamiana* accessions used by different research institutions most likely derive from a single source.

**Conclusions:**

This work provides gene annotation of the *N. benthamiana* genome, a genomic and transcriptomic characterization of a transgenic *N. benthamiana* line in comparison to its wild-type progenitor, and sheds light onto the relatedness of *N. benthamiana* accessions that are used in laboratories around the world.

**Electronic supplementary material:**

The online version of this article (10.1186/s12864-019-5960-2) contains supplementary material, which is available to authorized users.

## Background

*Nicotiana benthamiana* is an allotetraploid plant indigenous to Australia. The *Nicotiana* genus is a member of the *Solanaceae* family which is particularly relevant in agriculture, and includes potato (*Solanum tuberosum*), tomato (*Solanum lycopersicum*), eggplant (*Solanum melongena*), and the smoking tobacco (*Nicotiana tabacum*). The fame of *N. benthamiana* is however mostly due to its versatility for studies of plant-pathogen interaction and molecular farming rather than crop sciences [1–4]. During the last two decades this plant emerged as a very promising host for recombinant protein production, in particular for medical application as vaccines or antibodies [5–7].

Most prominently, the transgenic *N. benthamiana* line ΔXT/FT has been engineered [8] to act as a production system for therapeutic proteins and has been successfully used to produce antibodies at an industrial scale [5, 9, 10]. Its main feature is the knockdown of genes encoding fucosyl-transferases (FT) and xylosyl-transferases (XT) through RNA interference, a procedure that enables the production of recombinant glycoproteins with human glycan profiles *in planta*. Glycans influence protein folding and modulate protein activity [11, 12], and there is evidence that plant-specific glycan structures could potentially be antigenic to humans [13–15], even though this has been recently debated [16]. A linkage between core fucosylation and monoclonal antibody potency has also been described [17].

Despite *N. benthamiana*’s widespread use in research, and its growing importance as an expression platform for recombinant proteins, comparatively little is known about its genomic and transcriptomic features on the sequence level. In 2012, a first milestone was achieved with the publication of the Nb-1 draft genome assembly [18] that is available at the SOL Genomics Network website (https://solgenomics.net/) [19]. This assembly covers around 86% of the haploid genome size of *N. benthamiana*, which is estimated at 3.136 Gbp [3]. Another draft genome assembly was published the same year from a different research group [20], which published also a de novo assembled transcriptome in the following years [21, 22]. We also note the publication of a recent *N. benthamiana* gene set, which was obtained from mapping of genes identified in other *Nicotiana* species onto the *N. benthamiana* genome [23]. Here, we perform evidence-based gene prediction supported by 2.35 billion mRNA-seq reads and characterize the transcriptome. We use our predicted gene set to carry out genomic and transcriptomic analyses of the glycoengineered *N. benthamiana* line ΔXT/FT. We address the question where the two RNA interference cassettes have been inserted within the genome, and if the insertions might impact gene expression. For these comparisons, we generated additional high-coverage genomic and transcriptomic datasets from our parental *N. benthamiana* wild type line (WT) as well as the glycoengineered line ΔXT/FT derived thereof. We use transcriptomic data to explore whole-transcriptome differential expression between ΔXT/FT and WT, and we use the genomic data to identify single-nucleotide variants (SNVs) and insertion/deletion variants (indels) and discuss their functional impact. Finally, we address inter-accession relatedness between *N. benthamiana* lines in use at different research institutions. The lack of documentation for most of these lines makes it challenging to understand their real genetic diversity. The reproducibility of experimental results could in fact depend heavily on the genotype of the accession. By assessing the variants found within annotated coding regions of the *N. benthamiana* genome, we attempt to characterize this diversity.

## Results

### *N. benthamiana* gene catalogue and functional annotation

The Nb-1 draft genome assembly [18] comprising a total size of 2.97 Gbp with an N50 size of 0.5 Mbp was used as starting point to predict a gene set for *N. benthamiana*. We identified 60.7% of the sequence (excluding Ns) being composed of transposable elements (TEs) of which the majority belonged to the class of LTR retrotransposons (Additional file 1: Table S1), as expected for plant genomes [24, 25]. On the TE-masked Nb-1 genome we performed gene prediction using the Augustus pipeline [26]. A particular strength of Augustus is its combination of in silico gene prediction and integration of evidence from transcriptome sequencing, providing experimental support for the predictions. As transcriptomic evidence a total of 2.35 billion mRNA-seq reads from eight different *N. benthamiana* accessions were used, corresponding to 151.6 Gb of sequencing data; of these, 126 million reads (31.5 Gb) were generated in this study (Additional file 2). Data sources were chosen in a way that multiple tissues and stress conditions were represented. From 114,605 initial predictions we kept 62,216 transcripts (50,516 genes) that were supported by at least 1% mRNA-seq evidence and had no major overlap (max. 10 nt) with annotated TEs in coding regions; thirteen peptides of less than ten amino acids were removed from the set of protein sequences. The final set of gene predictions is referred to as “NibSet-1”. The average gene length including introns was 5,573 bp, the average transcript length was 1,665 bp, and the average protein length was 404 amino acids. The average number of exons per transcript was 6.2, and 59,410 transcript models (95.5%) included both start and stop codon (Table 1). Notably, 30,974 (61.3%) of the predicted gene models were fully supported by mRNA-seq evidence, i.e. all their predicted features, such as exon-intron junctions and UTRs, were supported by transcriptomic reads.

**Table 1:** *N. benthamiana* NibSet-1 gene set metrics

Genes	50,516
Transcripts	62,216
Protein sequences	62,203
Multi-isoform genes	8,676
Transcripts with start and stop codons	59,410
Average gene length	5,573 nt
Average transcript length	1,665 nt
Average number of exons per transcript	6.2
Number of single-exon transcripts	7,410
Average exon length	268 nt
Average length of coding exon (CDS)	213 nt
Average intron length	801 nt
Average protein length	404 aa

We used the fully supported models to test if they extend the gene set of an older gene prediction available at the SOL Genomics Network website [19], called Niben101_annotation. Most of the NibSet-1 high-confidence genes (26,817 of 30,974; 86.6%) overlapped at least for half of their length with a Niben101_annotation model of which 6,364 coincided perfectly when comparing annotated CDS coordinates. To verify the remaining 4,157 high-confidence NibSet-1 gene models we mapped them against the transcriptome of the paternal progenitor *Nicotiana sylvestris*. A large fraction (3,651 genes, 87.8%) found a match in *N. sylvestris* (minimum 90% sequence identity) and, hence, are likely to represent true genes that were missing in Niben101_annotation. We concluded that given the high amount of mRNA-seq data supporting our gene models, NibSet-1 is likely to be more accurate than Niben101_annotation and that NibSet-1 provides additional high-confidence genes that complement the gene models of Niben101_annotation. We also noted that the average protein length of Niben101_annotation was smaller (327 amino acids) than in NibSet-1 (404 amino acids, see above), suggesting that NibSet-1 was less fragmented than Niben101_annotation.

We validated the completeness of NibSet-1 by searching for sequence homology in a set of highly conserved plant genes using BUSCO (benchmarking universal single-copy orthologs) [27]. Out of 956 conserved plant genes, 937 (98.0%) were matched by a predicted *N. benthamiana* sequence (only one transcript per gene was used). For the sake of comparison, we ran BUSCO also on the Niben101_annotation gene set: 932 (97.5%) conserved plant genes were found (Additional file 1: Table S2) showing that highly conserved genes are well represented in both gene sets with a slightly higher level of completeness in NibSet-1 compared to Niben101_annotation.

Public NCBI databases [28] contained 401 *N. benthamiana* protein sequences (as of June 2017), of which 396 (98.8%) matched NibSet-1 protein sequences with a minimum sequence identity of 95%. All 401 sequences found a match with ≥85% sequence identity. Overall, we consider NibSet-1 to be a highly complete and accurate representation of *N. benthamiana*’s gene repertoire.

**Fig. 1:**
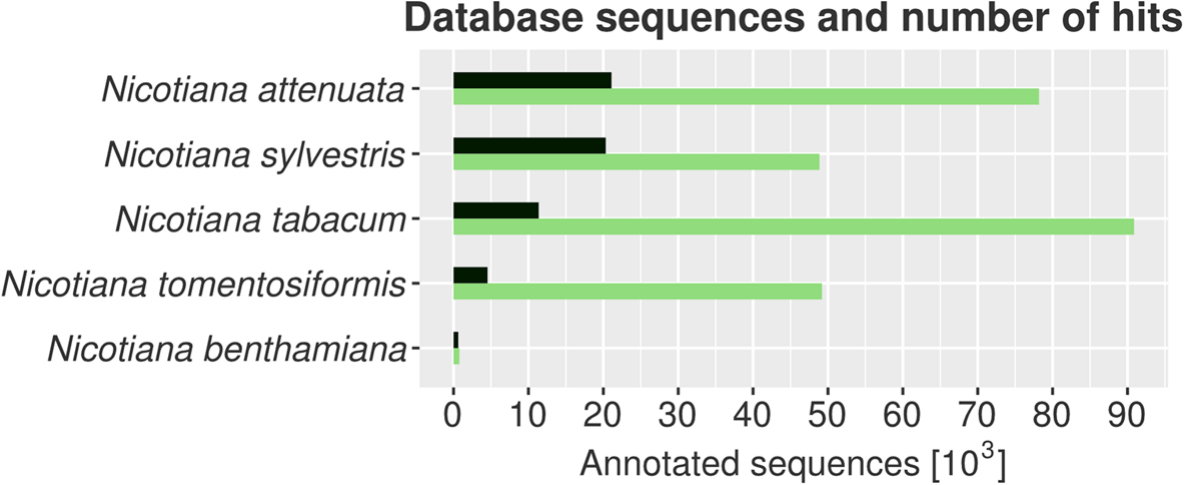
Blast best hits of NibSet-1 proteins on the five most represented *Nicotiana* species in the database. Shown are the number of database sequences belonging to each species (green), and the number of hits that were used for functional annotation (black)

We functionally annotated the NibSet-1 protein sequences by transferring annotations from homologous genes of other plant species (Additional file 1: Table S3) with sequence similarity ≥ 90% and alignment length ≥ 70 amino acids. In total, we assigned functional annotations to 44,184 (71%) *N. benthamiana* protein sequences belonging to 35,428 genes (Fig. 1). The majority (42,344 proteins, 95.8%) was annotated through homologous sequences from the *Nicotiana* genus, further annotations were transferred from the *Solanaceae* family (27 proteins), *Arabidopsis* (13 proteins), and “non-redundant” NCBI databases (1,800 proteins). Only 1,549 (2.5%) protein sequences corresponding to 1,499 genes could not find a match in any of the tested datasets.

### Characterization of transgene integration sites in the *N. benthamiana* line ΔXT/FT

The glycoengineered ΔXT/FT *N. benthamiana* line was generated to avoid the addition of the plant-specific glycan residues β1,2-xylose and core α1,3-fucose to recombinantly produced glycoproteins. This was achieved via the insertion of two transgenes (Additional file 3), which mediate down-regulation of the genes encoding core α1,3-fucosyltransferase (FucT) and β1,2-xylosyltransferase (XylT) by means of RNA interference [8]. In a recent study, five FucT genes have been described, with one of them probably representing a pseudogene [29]. Our raw gene set, prior to any filtering step, included all of them, i.e. FucT1 = g31184, FucT2 = g80352, FucT3 = g3481, FucT4 = g97519, FucT5 = g36277; gene g97519 was later removed due to an overlap with annotated transposable elements. The transgenes used in the glycoengineered ΔXT/FT *N. benthamiana* line were designed to act on at least two FucT genes (g31184 and g80352 in NibSet-1) and on both XylT genes (g40438 and g43728). We replaced Augustus FucT and XylT gene models in NibSet-1 (g31184, g40438, g43728, g80352) with the corresponding manually curated sequences from Strasser et al. (2008) (sequence identity 99%, see Additional file 1: Text; Figure S1).

**Fig. 2:**
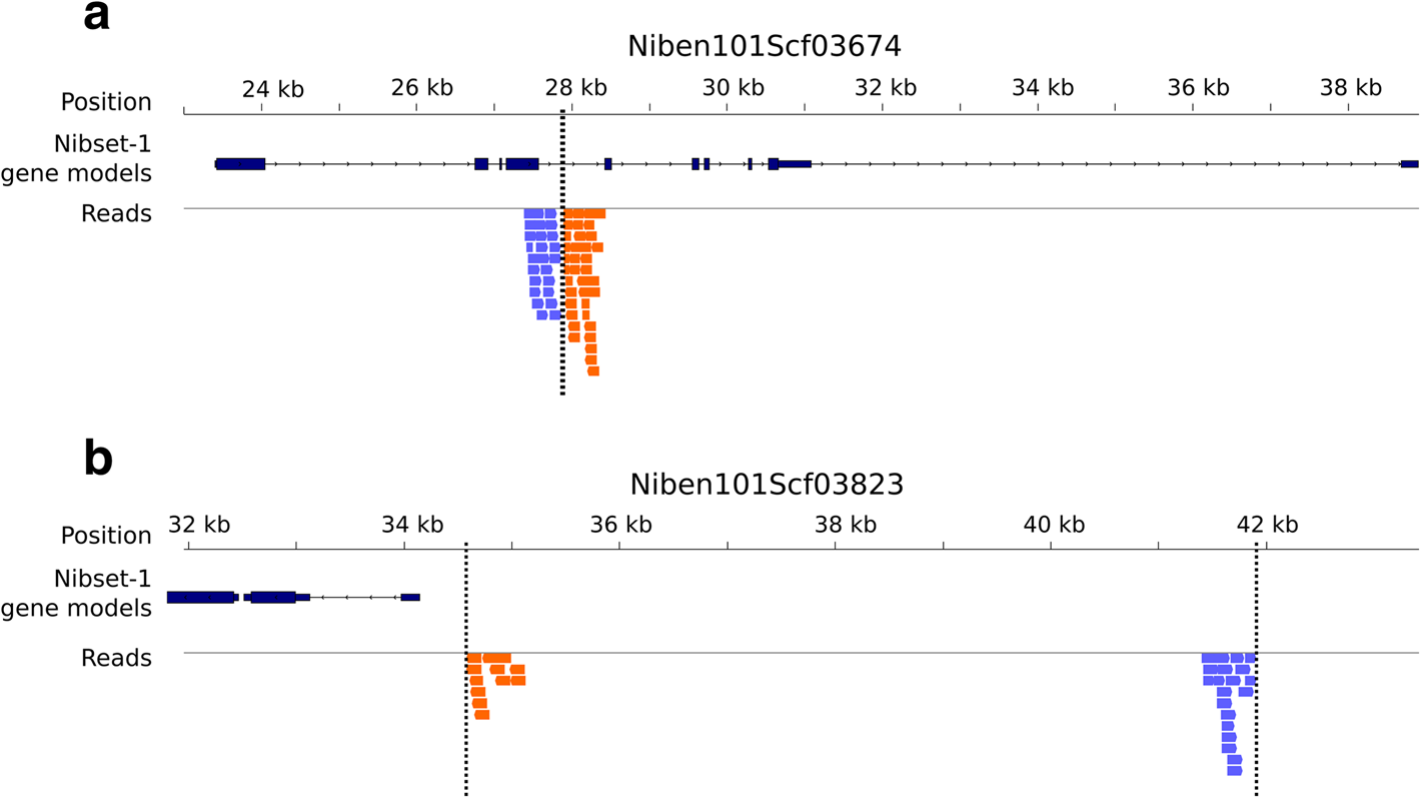
Identification of read-pairs connecting *N. benthamiana* genomic sequence to transgene sequences in ΔXT/FT. Mates of a read pair which establish a connection with the promoter fragment of a transgene are shown in light blue, those indicating a connection with a transgene terminator sequence are shown in orange. NibSet-1 gene models identified within or in proximity of the insertion regions are shown in dark blue. Dashed lines represent junctions identified with chimeric reads. **a** Transgene integration site within scaffold Niben101Scf03674 at a position between exons 4 and 5 of NibSet-1 gene g76921. **b** Transgene insertion site at scaffold Niben101Scf03823

Transgene insertion into the host genome occurs at positions that cannot be predicted [30]; it is therefore important to assess potential unintended changes to the genome upon transformation. To investigate this possibility, we generated Illumina paired-end genomic reads from the ΔXT/FT plant and from its wild-type parent, corresponding to 33-fold and 41-fold coverage, respectively, of the *N. benthamiana* genome (Additional file 2, code LF_DEX_3, LF_NIB_3). The transgenic constructs used in ΔXT/FT had a total length of 4.5 and 4.8 kbp, respectively, and were composed of the CamV35S promoter (2.8 kbp), the transgenic cassette (FucT-transgene, 1.1 kbp, or XylT-transgene, 0.8 kbp), and the 7TTR terminator region (0.9 kbp) [8]. We searched for the regions of the genome where the integration had taken place by identifying ΔXT/FT read pairs that had one mate mapping on the transgenic promoter or terminator sequence, respectively, and the other mate on the host genome represented by the Nb-1 draft assembly. For both transgenic constructs the whole sequence showed read coverage (Additional file 1: Figure S2), and we observed highly supported connections with Nb-1 scaffolds Niben101Scf03674 (62 pairs) and Niben101Scf03823 (32 pairs). We found promoter (P) and terminator (T) pairs clustering separately, defining the junction regions (Fig. 2). The clusters were composed of 34 P and 28 T pairs in Niben101Scf03674 and of 12 P and 20 T pairs in Niben101Scf03823. We note a difference between the two insertion sites in terms of number of bridging pairs. As outlined further below, the study of the insertion site in scaffold Niben101Scf03823 was problematic due to repetitive elements and assembly breakpoints. This likely reduced the ability of mapping reads to the region.

We performed a local alignment with the matching reads to localize the insertion position at base-pair precision by identifying chimeric reads that spanned the junctions between host genome and the transgenes. Supported by 10 P and 18 T chimeric reads we marked positions 27872 and 27901 as junction positions in Niben101Scf03674, and 11 P and 10 T chimeric reads supported positions 34601 and 41896 as junctions in Niben101Scf03823 (Fig. 2).

The location of mapped reads indicated that transgene integration in scaffold Niben101Scf03674 had led to a small deletion of 28 bases (Additional file 1: Figure S3).

**Fig. 3:**
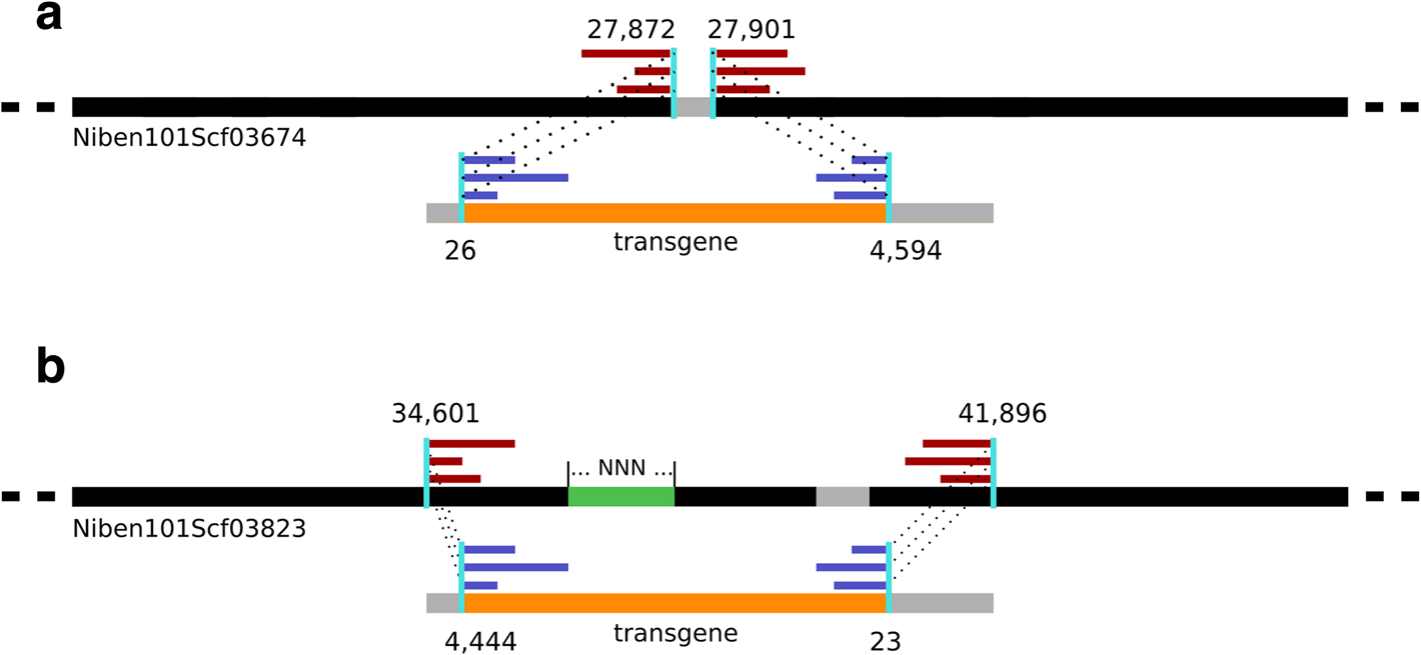
Organisation of transgene integration sites within the genome of *N. benthamiana* ΔXT/FT, as explored using chimeric sequencing reads spanning integration boundaries. **a** Expected model for the chimeric reads mapping, as exemplified by the insertion at scaffold Niben101Scf03674. Shown are chimeric reads mapping on the host genome with one side (dark red) and on the transgene promoter/terminator site with the other side (blue). The dashed lines connect the two sides, while the cyan vertical lines outline the boundary between the host genome and the transgene. Regions without read coverage are shown in gray, while covered regions are shown in black (host) or orange (transgene). **b** Transgene insertion site on scaffold Niben101Scf03823. A stretch of undetermined bases (~ 500 bp) within the Nb-1 assembly is indicated in green. The numbers over the cyan vertical lines indicate the junction positions on the Niben101Scf03823 scaffold. The proposed rearrangement of the region is shown in Additional file 1: Figure S4

In scaffold Niben101Scf03823 the context and the consequences of the insertion were less obvious (Fig. 2, panel “b”, Fig. 3). The gap density in the insertion region, a high amount of annotated TEs, and a coverage drop in ΔXT/FT may support a scenario whereby the region was misassembled in the Nb-1 draft and altered by a rearrangement that took place during transgene insertion (see Additional file 1: text; Figure S4).

### Molecular consequences of transgene insertions in ΔXT/FT

**Fig. 4:**
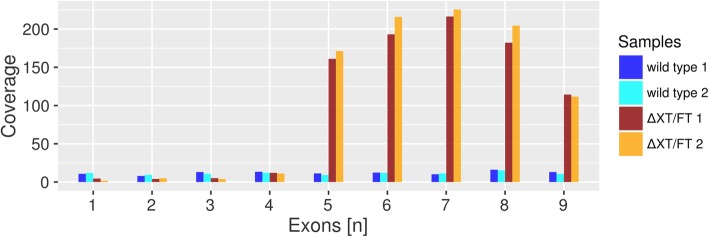
Per-exon transcriptomic coverage of NibSet-1 gene g76921, as detected with transcriptomic reads. WT replicates are indicated in blue and cyan, and ΔXT/FT replicates in red and orange, respectively. Increased coverage of exons 5–9 in ΔXT/FT is the result of transgene integration which leads to the production of a fusion transcript under control of the strong CamV35S promoter

In the case of scaffold Niben101Scf03823, our data supported transgene insertion in a region consisting of non-coding, highly repetitive DNA, where no predicted gene was disrupted by the insertion. Therefore, this insertion site was considered as not critical regarding its functional impact. In contrast, the inferred insertion site in the region corresponding to scaffold Niben101Scf03674 was located within intron 4 of gene g76921, encoding for TFIID subunit 12-like isoform X1, a subunit of an important general transcription factor [31]. Analysing mRNA-seq data from ΔXT/FT (see below), the expression profile of this gene showed a much higher transcriptomic coverage in the exons downstream of the insertion site (exons 5–9) than in the exons further upstream (Fig. 4). This supported the idea that the transgene under the control of the CamV35S promoter had become fused to the exons of g76921 from exon 5 onwards in ΔXT/FT. Indeed, we found 11 transcriptomic read pairs that confirmed the occurrence of such a fusion transcript: these read pairs showed one mate mapping onto g76921 and the other mate mapping onto the FucT-transgene, unequivocally assigning its integration site to scaffold Niben101Scf03674. Therefore, we could infer that the XylT transgene insertion had occurred on scaffold Niben101Scf03823. However, no formal proof of this conclusion was possible due to highly repetitive sequences surrounding the integration site. Read pairs which linked the FucT transgene to g76921 mapped not only to exon 5 but also to exons 6 to 8, respectively, indicating that exons downstream of the insertion site kept their original splicing pattern. We concluded that the g76921 locus was disrupted in ΔXT/FT, and a fusion transcript composed of the FucT-transgene RNA attached to the normally spliced exons 5 to 9 of g76921 was present. Notably, we did not find read pairs linking exons 4 and exon 5 (i.e. no support for the presence of the wild type allele), indicating homozygosity, with both alleles of g76921 being disrupted. However, we considered a disruption of g76921 as not harmful to ΔXT/FT since there is another actively expressed gene copy annotated as TFIID subunit 12-like isoform X1 (g54961, 86% protein seq. Identity; Additional file 1: Figures S6, S7, S8). In principle, g54961 may be sufficient to buffer the loss of function of g76921; however, its TPM expression value in ΔXT/FT (12.6 ± 0.4) was comparable to the one observed in WT (13.8 ± 1.5) and the resulting log-2-fold change was negligible (− 0.029).

### Analysis of the ΔXT/FT transcriptome

**Fig. 5:**
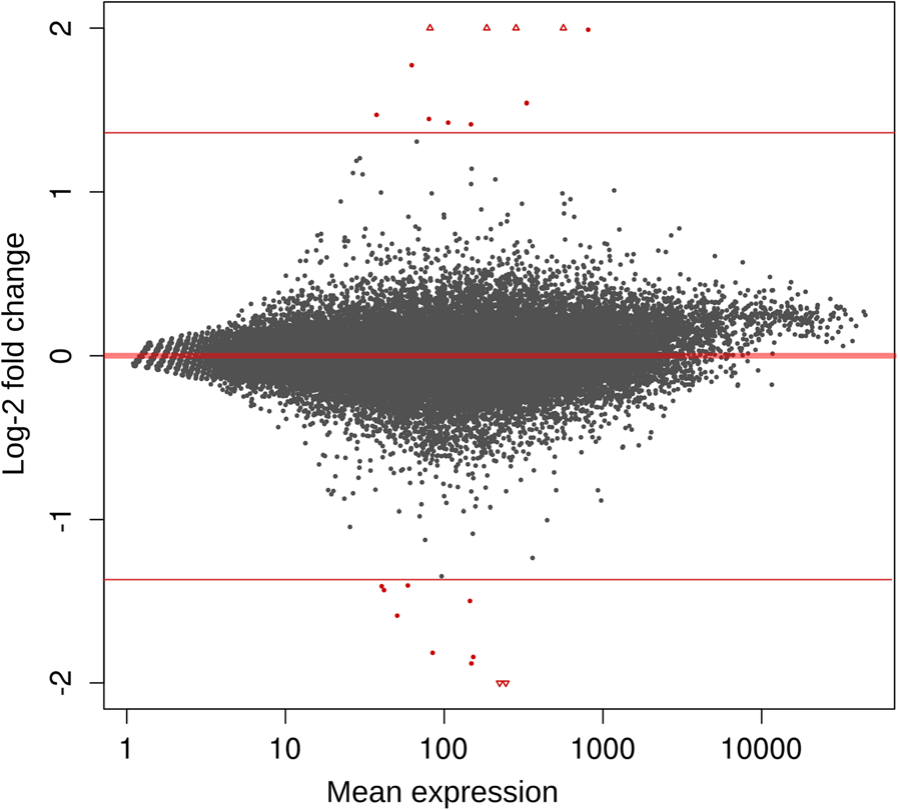
Comparison of global gene expression in leaves of the *N. benthamiana* wild type WT and the transgenic line ΔXT/FT. Log2-fold change (LFC) is plotted against mean expression (counts) for each NibSet-1 gene. Red dots represent genes with a sufficient mean expression, LFC and adjusted *p*-value to be considered differentially expressed genes (DEGs). Triangles represent genes whose LFC value exceeded the plot margins [− 2,2]. Thin red lines enhance the separation between DEGs and the other genes. Eleven genes are found upregulated (log2-fold change > 1.40) and ten genes are found downregulated (log2-fold change < − 1.40) in ΔXT/FT compared to the *N. benthamiana* wild type

The perturbation of the ΔXT/FT genome upon transgene insertion might have unpredictable effects on the plant’s transcriptome. We therefore generated leaf mRNA-seq data from ΔXT/FT and its wild type (WT) parent, both in duplicate. The paired-end reads were quality-trimmed and mapped against the Nb-1 draft genome assembly, using NibSet-1 gene models as guide for mapping. We extracted the raw counts for each gene in each replicate and condition; the counts were then normalized to the sequencing depth of the corresponding replicate. Genes with low mean coverage across replicates and samples (< 10) were removed. We assessed the potential presence of artifacts in the normalized counts through a principal component analysis (PCA). The PCA outlined no clear distinction between conditions and replicates (Additional file 1: Figure S9). Pearson’s correlation scores calculated between the four samples were all ≥ 0.9 (Additional file 1: Table S8). We concluded that the transcriptome in WT and in ΔXT/FT are likely to be highly comparable. From the normalized counts of the retained genes we computed Fragments Per Kilobase of exon per Million fragments mapped (FPKM) and Transcripts Per Million (TPM) for each gene. We then computed log2-fold changes (LFC) between the two genotypes (Additional file 4). Considering the high correlation between the samples we made sure that even moderate variation in gene expression were considered; hence, we considered as differentially expressed every gene showing a LFC ≥ 0.5. The test returned a group of 21 differentially expressed genes (DEGs), all with LFC values substantially higher than the 0.5 threshold (≥ 1.40, Fig. 5). From this list we removed seven genes having a TPM value below the sample-specific TPM threshold (indicated in the Methods section) in both conditions.

**Fig. 6:**
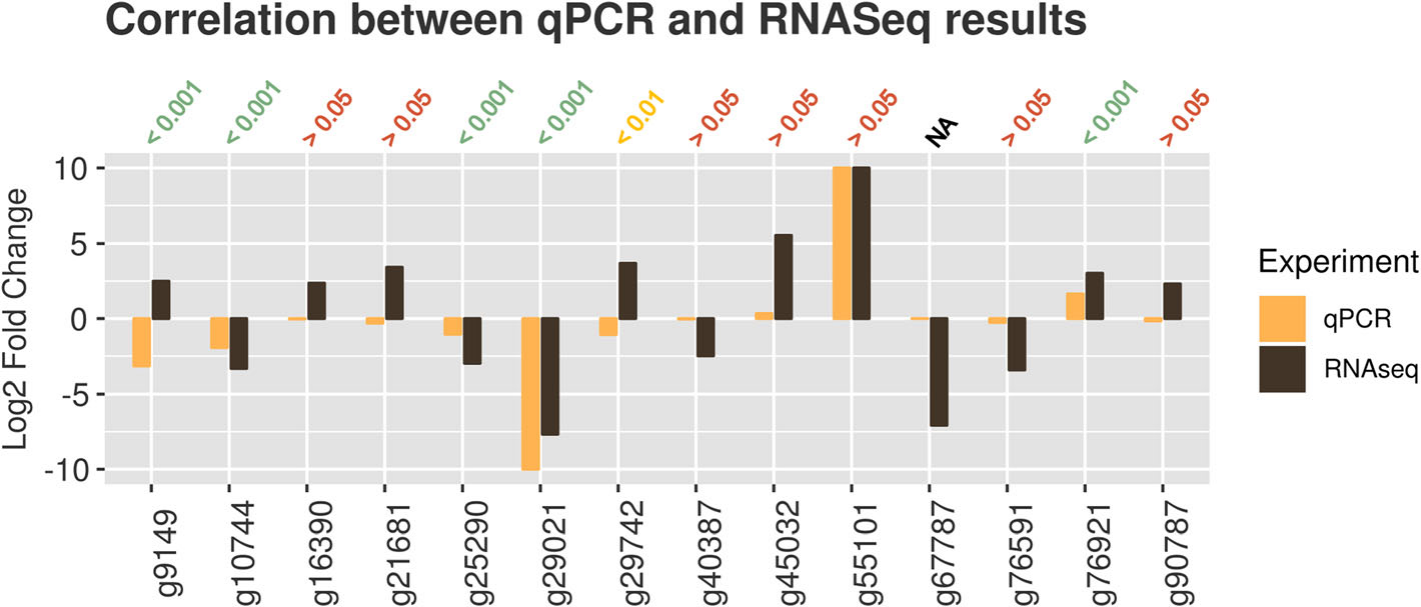
Comparison of differential gene expression as assessed by mRNA-seq and quantitative PCR, respectively. Positive log2-fold change indicates upregulation in ΔXT/FT relative to wildtype, negative log2-fold change indicates downregulation in ΔXT/FT. Values above the table indicate two-tailed *p*-values computed in unpaired *t* tests between ΔXT/FT and WT qPCR results. Red: not significant, yellow: moderately significant, green: highly significant. Not available (“NA”) indicates absence of qPCR signal. We kept for further analyses only genes where both qPCR and mRNA-seq indicated up-regulation or down-regulation, respectively. Gene numbers refer to NibSet-1 genes. The plot is limited to the range [− 10, + 10]

We performed quantitative PCR in triplicate for the remaining 14 DEGs in order to confirm their differential expression. Unpaired *t* tests between ΔXT/FT and WT were performed to test the statistical robustness of each qPCR observation; we retained only those showing the same expression trend and a two-tailed *p*-value < 0.05. We confirmed one up-regulated gene (g76921) as well as three down-regulated genes (g10744, g25290, g29021) (Table 2, Fig. 6, Additional file 1: Figure S10). We note the presence of g76921 among the upregulated DEGs, which was disrupted by the insertion of the FucT-transgene (see above). Through interPro [32] we catalogued protein family, annotated domains, repeats, signature matches, and GO terms of the confirmed DEGs, none of them being directly involved in protein glycosylation. Notably, the four genes targeted by the transgenes (g31184, g80352, g43728, g40438) were not found among the five DEGs. This is most likely due to the efficiency of the knockdown system. We did, in fact, observe a generalized decrease in normalized read counts for the targeted genes in ΔXT/FT with respect to WT (Additional file 1: Table S4). We note that, while the transgenes were designed to act post-transcriptionally, potential homology of their promoter with that of other host genes could have triggered transcriptional gene silencing *in trans* [33–35], altering their transcription. As our results show that this was not the case, we conclude that ΔXT/FT has a transcriptional profile which is highly comparable to the wild type, with the exception of the transgene knockdown of FucT and XylT.

**Table 2:** Differentially expressed genes (DEGs) between wild type *N. benthamiana* and the ΔXT/FT transgenic line based on a comparison of leaf mRNA-seq data and confirmation by quantitative PCR

Gene ID	Function	*E*-Value	Identity	TPM ΔXT/FT	TPM WT
Downregulated genes					
g10744	uncharacterized oxidoreductase At4g09670-like	0	96%	1.9 ± 0.6	18.5 ± 2.4
g25290	alpha-soluble nsf attachment	0	100%	4.3 ± 0.4	34.0 ± 1.1
g29021	PREDICTED: LOW QUALITY PROTEIN: primary amine oxidase-like	0	92%	0.1 ± 0.0	20.4 ± 1.3
Upregulated genes					
g76921	transcription initiation factor TFIID subunit 12-like isoform X1	0	85%	41.4 ± 2.8	5.1 ± 0.0

Gene IDs refer to NibSet-1. The protein sequences of the identified DEGs were mapped on the blast Eudicots database (taxid: 71240)

### Genomic variants in ΔXT/FT

We screened the genome of *N. benthamiana* ΔXT/FT for differences (i.e. variants) that could have accumulated after the generation of ΔXT/FT, dated 2008, during at most 40–50 estimated generations by 2015, when the samples were taken and sequenced. The genotype Nb-1, an inbred *N. benthamiana* line that had been maintained in the laboratory of Gregory B. Martin since the mid-1990s [18] was used as a reference.

We re-sequenced the genomes of both ΔXT/FT and WT to approximately 33-fold and 41-fold respective genomic coverage on the Illumina sequencing platform (Additional file 2, codes LF_DEX_3 and LF_NIB_3) and used the reads to call variants relative to the Nb-1 reference genome (see methods for details). To exclude consensus errors in the assembly, we mapped genomic reads from the Nb-1 genotype against the Nb-1 assembly and removed all varying positions from the analysis (Table 3, panel “a”). After this filtering step, 96,510 SNVs and 6,605 indels were detected between ΔXT/FT and Nb-1; 106,079 SNVs and 7,217 indels were detected between WT and Nb-1 (Table 3, panel “b”); in both cases a transition/transversion (Ti/Tv) ratio of 1.4 was observed. To obtain a list of ΔXT/FT specific variants, we removed 57,362 SNVs and 2,478 indels shared by both genotypes against the Nb-1 reference; In this way, 39,148 SNVs and 4,127 indels specific to ΔXT/FT were retained. Of these, 3,036 SNVs and 80 indels were found within coding regions (CDS) (Table 3, panel “b”). The Ti/Tv ratio within CDS was higher (1.8) than in the whole variant pool (1.4); this could be due to higher selective pressures against transversions in coding regions [36]. We annotated the impact of each variant with the program SnpEff [37] which returned 67 variants (23 SNVs, 44 indels) in different genes annotated as “high impact” variants (Additional file 5). We extracted GO terms for the proteins encoded by these genes, retrieving terms for 29 proteins (43.3%). However, with a false discovery rate (FDR) < 0.05, we found no statistically significant GO term enrichment.

**Table 3:** Number of single-nucleotide variants (SNVs), number of insertion/deletion variants (indels) and transition/transversion (Ti/Tv) ratio for each comparison performed

Line	SNVs	Indels	Ti/Tv
a			
ΔXT/FT vs Nb-1	117,278	7,626	1.4
WT vs Nb-1	127,976	8,257	1.4
Nb-1 vs Nb-1	56,930	4,505	1.3
b			
ΔXT/FT vs Nb-1	96,510	6,605	1.4
WT vs Nb-1	106,079	7,217	1.4
Shared	57,362	2,478	1.4
ΔXT/FT unique	39,148	4,127	1.4
ΔXT/FT unique (CDS)	3,036	80	1.8

a) Raw number of variants before filtering out consensus errors, and b) after filtering out consensus errors, including subsets of variants relevant in the analysis. “Shared”: variants shared between ΔXT/FT and WT relative to Nb-1. “ΔXT/FT unique”: variants found only in ΔXT/FT relative to Nb-1. “ΔXT/FT unique (CDS)”: variants found only in ΔXT/FT relative to Nb-1 restricted to coding regions

### Genetic relatedness of *N. benthamiana* research accessions

A recent study posits that today’s laboratory strains of *N. benthamiana* are all derived from a single specimen collected in the central Australian desert [38, 39]. The two draft genome assemblies available [18, 20] diverge by one SNV every 2,900 base pairs, i.e. 345 SNV/Mbp [38]. To assess whether we could obtain comparable data based on coding regions, we selected seven *N. benthamiana* accessions from which public mRNA-seq data were available (Additional file 2), maintained at the following research institutions: China Agricultural University, Beijing, China; King Abdul Aziz University, Jeddah, Saudi Arabia; National Academy of Agricultural Sciences, Jeonju, South Korea; University of Sydney, Sydney, Australia; Swedish University of Agricultural Sciences, Uppsala, Sweden; University of Natural Resources and Life Sciences (BOKU), Vienna, Austria. From BOKU both the WT and ΔXT/FT accessions used in this study were included. We quality-trimmed reads from each accession, selected 14 million reads each and cropped them to a length of 48 nt. The number of reads extracted was chosen according to the maximum number available from each sample after quality filtering (smallest dataset: *N. benthamiana* accession from Jeonju, South Korea, 14 million reads). The cropping length was decided according to the longest common sequence length available after trimming (shortest reads: *N. benthamiana* accession from Uppsala, Sweden, 48 nt). As some of the datasets were single-end reads, the paired-end samples were processed using only the first read of each pair. The Nb-1 draft genome assembly was used as a reference for mapping.

For each obtained call set we computed the SNV/Mbp ratio dividing the number of SNVs by the positions (in Mbp) covered by the reads (min. Coverage 4x) limiting the computation to CDS regions only. All of the seven tested accessions showed similar rates, with an average of 67 SNV/Mbp (range: 64–75). The lowest recorded rate of SNV/Mbp belongs to the sample from Jeddah, Saudi Arabia, although we note that all of the values were in a very narrow range (Table 4). These values are compatible with the aforementioned divergence estimates by [38]: our estimates were obtained using coding regions, hence variation is expected to be lower than in whole-genome comparisons. The coding sequence-based divergence estimates are all very similar, supporting a scenario whereby the tested accessions display high genomic relatedness.

**Table 4:** Number of single-nucleotide variants (SNVs) obtained by mapping of mRNA-seq data from *N. benthamiana* and *N. sylvestris* against the Nb-1 reference genome sequence, considering only variants within coding exons

	Cov. Positions	SNVs	SNVs/Mbp
WT (AT)	8,630,008	556	64
ΔXT/FT (AT)	8,651,732	562	65
LAB (AU)	11,483,694	789	69
*N. benthamiana* (CN)	6,574,943	495	75
*N. benthamiana* (KR)	10,517,109	695	66
*N. benthamiana* (SA)	8,717,762	562	64
*N. benthamiana* (SE)	11,074,510	719	65
*N. sylvestris*	7,990,760	65,140	8,152

Covered positions: positions with a minimum coverage of 4x; SNVs: total number of variants detected in coding regions; SNVs/Mbp: number of variants per Megabase of coding sequence. Sample names are specified in the first column. Countries of origin are specified as follows: Australia (AU), Austria (AT), China (CN), Saudi Arabia (SA), South Korea (KR), Sweden (SE)

As a control, we used mRNA-seq reads from the presumable *N. benthamiana* paternal subgenome donor *N. sylvestris* [40] processed with the same pipeline; we obtained 8,152 SNV/Mbp distributed in 7,990,760 bp (Table 4). We also confirmed the validity of the variants within coding regions using contigs obtained by assembling ΔXT/FT genomic reads (see Additional file 1: Text). We observed a concordance of 84% between calls from mRNA-seq data (ΔXT/FT cDNA reads) and calls from contig mapping (124 mRNA-seq SNVs in agreement, 24 in disagreement).

As a means of comparison we analysed the variant density observed between *A. thaliana* accessions. For once, we called variants in annotated coding regions using mRNA-seq reads from six *A. thaliana* ecotype Col-0 derived lines in comparison to the TAIR10 reference genome assembly [41], using the same parameters as for *N. benthamiana*. Further, we used Col-0 mRNA-seq reads and mapped them against 13 different *Arabidopsis* genome assemblies of wild accessions generated in the 1001 genomes study [42]. Col-0 intra-accession diversity was very low (2 SNV/Mbp: range: 1–3 SNV/Mbp), while many more variants were observed in comparison to wild-derived accessions (1742 SNV/Mbp; range: 1447–2178 SNV/Mbp) (Table 5, panels “a” and “b”).

**Table 5:** Number of single-nucleotide variants (SNVs) obtained by mapping of mRNA-seq data from *A. thaliana* against the TAIR10 reference genome sequence

	Cov. Positions	SNVs	SNVs/Mbp
a			
Col-0 (CN)	9,098,019	24	3
Col-0 (DE)	10,839,185	12	1
Col-0 (JP)	12,819,475	18	1
Col-0 (MX)	10,992,622	20	2
Col-0 (NL)	11,479,175	23	2
Col-0 (US)	12,320,980	21	2
b			
No-0 (DE)	13,205,980	22,006	1,666
Sf-2 (ES)	13,174,328	23,169	1,759
Can-0 (ES)	13,095,023	28,515	2,178
Edi-0 (GB)	13,198,944	22,051	1,671
Bur-0 (IE)	13,172,042	25,137	1,908
Ct-1 (IT)	13,207,544	23,498	1,779
Tsu-0 (JP)	13,205,663	21,836	1,654
Mt-0 (LY)	13,220,021	21,953	1,661
Kn-0 (LT)	13,185,117	23,141	1,755
Hi-0 (NL)	13,212,525	19,123	1,447
Ler-0 (PL)	13,216,378	21,857	1,654
Ws-0 (RU)	13,194,655	22,999	1,743

Only variants in coding exons were considered. Covered positions: positions with a minimum coverage of 4x; SNVs: total number of variants detected in coding regions; SNVs/Mbp: number of variants per Megabase of coding sequence. a) mRNA-seq data from *A. thaliana* ecotype Col-0 mapped against TAIR10. Provenance of each accession is indicated: China (CN), Taiwan (TW), Japan (JP), Mexico (MX), Netherlands (NL), United States of America (US). b) mRNA-seq data from Col-0 “NL” mapped on genome assemblies from thirteen different wild-derived *A. thaliana* accessions. Ecotype name and country of origin is indicated. Country codes: Germany (DE), Ireland (IE), Italy (IT), Japan (JP), Libya (LY), Lithuania (LT), Netherlands (NL), Norway (NO), Poland (PL), Russia (RU), Spain incl. Canary islands (ES), United Kingdom (GB)

## Discussion

Providing a set of predicted genes along with a draft genome sequence increases greatly the molecular resources for further analyses of a species. Although the existing draft assembly of *N. benthamiana* was based only on short-read sequencing data we were able to predict a large proportion of full-length transcripts including start and stop codon. The gene set was established using comprehensive mRNA-seq data generated in this study and validated by two independent approaches both demonstrating its high level of completeness. To avoid the inclusion of transposable elements we performed repeat masking and posterior filtering of predicted genes that overlapped with repeat annotations. In this way, we lost one of five described FucT genes in the final gene set although it had been predicted initially. Further genes may be filtered out similarly, however, the prediction procedure aimed for a minimized repeat content in the final gene set. The majority of our predicted *N. benthamiana* genes could be matched by functionally annotated genes from other species providing additional valuable information on the *N. benthamiana* gene set and validating the predictions once again. Complementing existing data of *N. benthamiana* we generated genomic sequencing data from two additional *N. benthamiana* accessions one of which was the engineered ΔXT/FT line. Two genomic regions of interest were analysed in detail, i.e. the insertion sites of transgenes for silencing of FucT and XylT genes involved in glycan addition to proteins. While the genomic locations of insertion and corresponding sequence scaffolds could be identified and assigned to each transgene we found a differing amount of genomic read data matching the two transgene insertion sites. This indicated a rather complex scenario for the insertion site of the XylT transgene including repetitive regions, genomic rearrangements, and a potential misassembly in Nb-1, all of which limited the mappability of sequencing reads. The FucT transgene insertion site was covered well by sequencing reads from the ΔXT/FT line revealing transgene insertion within a gene that most likely lost its function. Since another intact copy of a closely related homolog was detected in the genome no harmful effect is to be expected. Transcriptome analysis did not show remarkable differences between ΔXT/FT and the wild type demonstrating specific transgene activity. Further differences between the two lines were only minimal. When comparing several *N. benthamiana* lines used in research laboratories our data suggested that the *N. benthamiana* lab lines tested here were more closely related to each other than wild-derived *A. thaliana* accessions. At the same time, higher divergence existed between *N.benthamiana* lines in comparison to *A. thaliana* Col-0 derivatives. Even though *N. benthamiana* research strains have recently been reported to originate from one source [38, 39], to the best of our knowledge no effort has been made to preserve and maintain a genetically homogeneous strain as is the case for the *A. thaliana* Col-0 ecotype; this might result in the slightly higher variation among *N. benthamiana* accessions that we have observed. All in all, our data confirmed the hypothesis that all currently used *N. benthamiana* laboratory accessions derive from the strain collected at the Australian Granites site [38].

## Conclusion

Over the years, the interest in *N. benthamiana* as an *in planta* protein expression platform has grown considerably, and much information has been accumulated. The gene set presented here, comprising 50,516 genes transcribed in 62,216 isoforms reflects this knowledge gain. However, our functional annotation results also show the lack of information still present: only 71% of the transcriptional isoforms could be functionally annotated. Further research will have to fill this information gap. Our study also showed the need for a genome and transcriptome analysis when using a transgenic plant: the identification of disrupted genes, their potentially altered expression, their copy number, and the zygosity of the insertion are important factors to detect any side-effects of the transgene insertion. The insertion sites of the two transgenes in ΔXT/FT could be located, even though the position of only one insertion could be identified on the nucleotide level. In this study, we also addressed variation within the whole genome and within coding regions, respectively, as a mean to determine accession relatedness. We show that the variation within coding regions is compatible with a scenario whereby the LAB strain is at the root of all accessions used in *N. benthamiana* research [38].

## Methods

### Plant material and isolation of nucleic acids

Seeds of wild-type *Nicotiana benthamiana* plants originally described by Regner and co-workers [43] were provided by Herta Steinkellner (University of Natural Resources and Life Sciences, Vienna). *N. benthamiana* ΔXT/FT is regularly grown in the lab of co-author Richard Strasser who also developed the line [8]. Wild type and ΔXT/FT plants were grown on soil in a growth chamber at 22 °C with a 16-h-light/8-h-dark photoperiod. For extraction of nucleic acids, leaves from 5-week-old plants were immersed in liquid nitrogen and macerated with grinding balls in a mixer mill. Genomic DNA was isolated from 1.5 g leaves using a Nucleospin Plant II Maxi kit (Macherey-Nagel, Düren, Germany) according to the instructions of the manufacturer. RNA was isolated from 40 mg leaves using the SV Total RNA isolation kit (Promega, Madison, WI, USA).

### Library preparation and Illumina sequencing

One microgram of genomic DNA was sheared in a S220 Focused-ultrasonicator (Covaris, Woburn, MA, USA) using covaris microtubes with a duty cycle of 10, intensity 5 and a cycle/burst of 200 for 35 s in order to achieve a peak fragment length of 700 bp. Genomic libraries were prepared using the NEBNext Ultra sample preparation kit (New England Biolabs, Ipswich, MA, USA) according to the recommendations of the manufacturer. Size selection of the libraries was performed on a 2% agarose gel with 1xTAE buffer. A gel slice containing the library fragments of interest was processed using the QIAgen gel extraction kit (Qiagen, Hilden, Germany) and further purified using QIAquick columns. Thereafter, the library was amplified using 7 cycles of PCR. Finally, the library quality was assayed on a DNA1000 chip using an Agilent 2100 Bioanalyzer (Agilent, Santa Clara, CA, USA). Library quantity was assessed on a Qubit fluorometer (Thermo Fisher Scientific, Waltham, MA, USA). From ΔXT/FT and from the corresponding wild type line, we obtained 414 million and 508 million raw read-pairs, respectively (Additional file 2, codes LF_DEX_3, LF_NIB_3). This translates into a genomic coverage of 33-fold (ΔXT/FT) and 41-fold (wild type), assuming a genome size of 3.1 Gbp.

mRNA-seq libraries were generated on a Tecan robotic workstation using the TruSeq stranded mRNA library prep kit (Illumina, San Diego, CA, USA) starting with 1 μg of total RNA. During RNA purification, genomic DNA was digested with RNase-free DNase I (Promega, Madison, WI, USA). Libraries were amplified using 15 PCR cycles. Library quality and quantity was assessed as above. Sequencing was performed in paired-end mode on the Illumina HiSeq 2500 with v4 sequencing chemistry using a 2 × 125 cycle protocol. We obtained between 28 and 38 million raw read-pairs per mRNA-seq library (Additional file 2, codes LF_DEX_1 and 2, LF_NIB_1 and 2).

### Gene prediction

Raw reads (Additional file 2) were analyzed with FastQC [44]. Read trimming was conducted with Trimmomatic [45] (ILLUMINACLIP:TruSeq2-PE.fa:2:30:10 LEADING:3 TRAILING:3 SLIDINGWINDOW:4:15 AVGQUAL:30 MINLEN:36). The Nb-1 draft genome assembly [18] (v1.01, downloaded in January 2016) available at the SOL Genomics Network [19] was used as a reference for the mapping step. With RepeatModeler [46] (−engine ncbi) we generated a library of repetitive elements on this draft genome assembly. Only repeats belonging to the DNA elements, LTR, LINE, SINE, Helitron and Unclassified families were retained, in order to mask transposable elements which can interfere with gene prediction [47]. RepeatMasker [48] (−engine ncbi -gff -noisy -no_is -norna -nolow) was used to generate a masked version of the Nb-1 genome, together with an annotation in GFF format.

We mapped the transcriptomic reads (Additional file 2) to the Nb-1 draft assembly with BLAT [49] (−tileSize = 11 -minIdentity = 92 -stepSize = 11 -minMatch = 2 -maxGap = 2 -oneOff = 0) and with TopHat2 [50] (−-read-mismatches 2 --read-gap-length 2 --max-insertion-length 3 --max-deletion-length 3 --b2-sensitive --microexon-search). PCR duplicates were removed. The results were filtered with samtools [51] keeping only primary alignments (samtools view -F 0×0100). Expression hints from the mapping results of BLAT and TopHat2 were computed separately and combined, giving priority to TopHat2 results in case of conflicts. With the script RNA-seq-noise-reduction.pl [52] we increased the contrast between exon and intron regions. We further limited the hints coverage by applying a minimum coverage of 20 and a maximum coverage of 300 to each hint to reduce background noise. The combined mRNA-seq information was merged with the information on annotated repeats, yielding 72,940,895 hints for exonic positions (genome positions with mRNA-seq coverage), 583,572 hints for introns (full intron span defined by reads mapped in spliced mode) and 1,994,352 hints for repetitive sequences (from RepeatMasker, see above). The unmasked Nb-1 draft genome assembly was split into 50 segments of similar size to parallelize the analysis. We provided repeat information in the hints file, instead of using the masked genome [52, 53]. Each segment was then submitted to the Augustus pipeline [26] (alternatives-from-evidence=true, allow-hinted-splicesites=atac, species=coyote_tobacco).

### Gene set filtering and validation

The raw gene set generated by Augustus was filtered by removing gene structures with < 1% coverage by expression hints. We removed peptides of length < 10 amino acids from the protein set of sequences. We filtered out the genes that overlapped with annotated TEs by more than 10 nt in their coding regions. The consistency between mRNA-seq expression profiles and gene models was assessed for 200 randomly chosen genes with GBrowse2 [54] adding separate data tracks for expression evidence and for transposable elements. We assessed correlation between predicted exons and read coverage, between predicted introns and split-mapped reads, and the absence of annotated TEs in the coding regions. The Niben101_annotation gene set was downloaded from the SOL Genomics Network website (https://solgenomics.net/) [19], from the ftp repository corresponding to *N. benthamiana* (v101). The overlap between gene models was determined using bedtools intersect [55]. The concordance between annotated CDS regions was assessed with a custom Python script. The completeness of the gene set was verified with BUSCO [27] (−m OGS), using the BUSCO plant database (http://busco.ezlab.org/). To avoid biases in the duplicated BUSCOs counts we used only one sequence per gene, corresponding to its longest isoform. The BUSCO validation was run on both NibSet-1 and Niben101_annotation. *N. benthamiana* cDNA sequences were downloaded from GenBank [56]. The sequences were converted to protein sequences and mapped against the proteins of the newly generated gene set using BLAT [49] (−minIdentity=85). The PSL-formatted results were then filtered by sequence identity and alignment length.

### Functional annotation

**Fig. 7:**
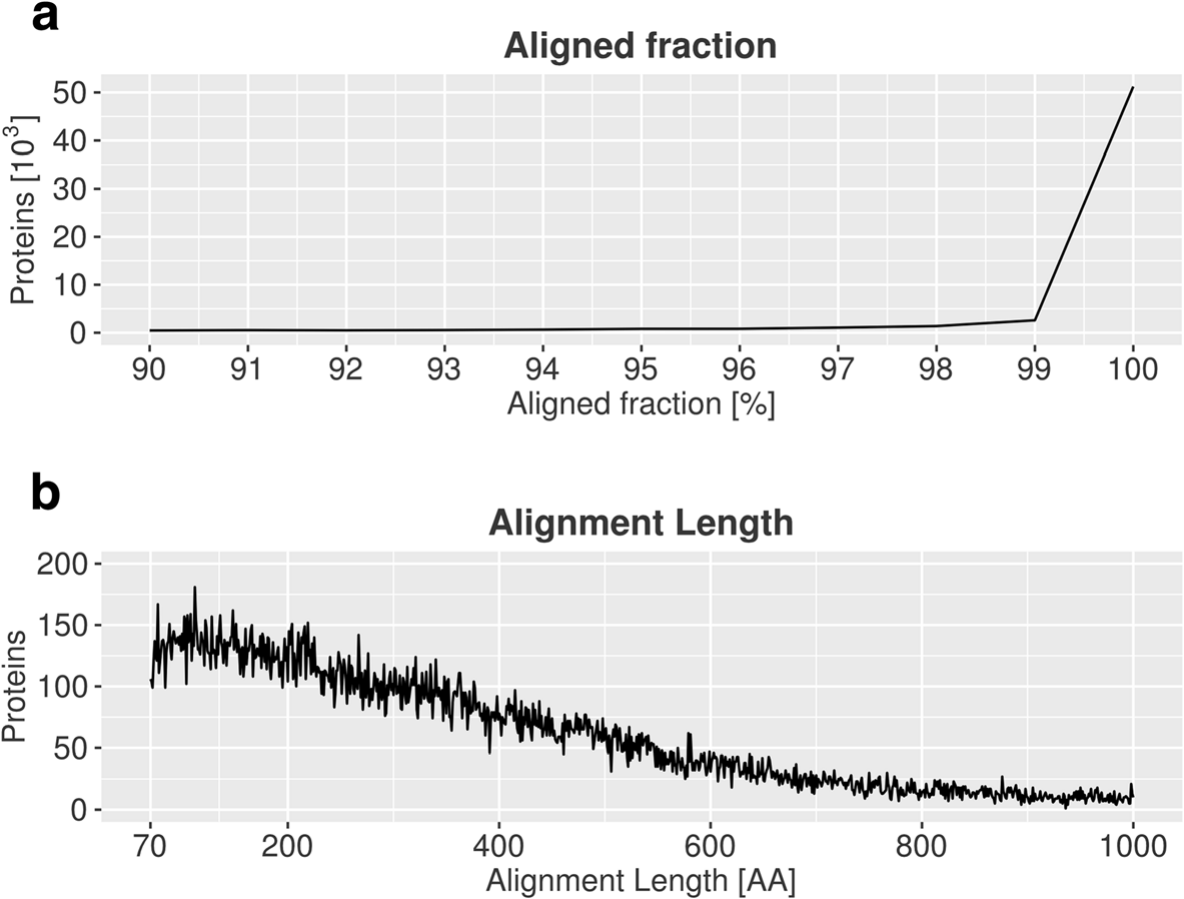
Comparison of NibSet-1 protein sequences against protein sequence databases. **a** Fraction of the sequence of each protein in the NibSet-1 transcriptome aligned to its best match in one of the blast databases used in this study. **b** Length in amino acids of such alignments

**Fig. 8:**
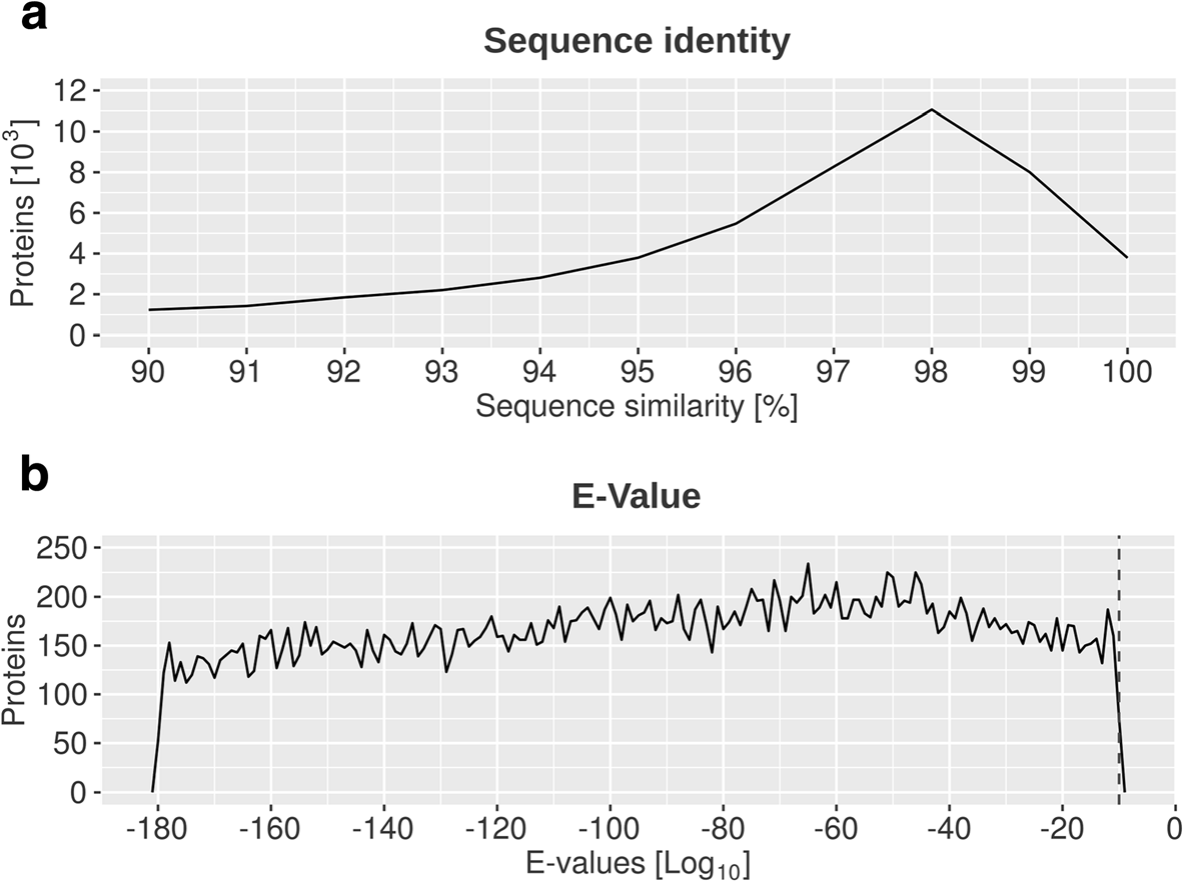
Comparison of NibSet-1 protein sequences against protein sequence databases. **a** Sequence identity retrieved for each blast search. As the search was limited to results with at least 90% identity, the plot range is restricted from 90 to 100% identity. The y-axis shows the number of proteins (in thousands) matching at each level of identity. **b** Log10 of the e-values associated with each blast search. Results with e-values >10e-10 were discarded (dashed line)

The validated gene set was functionally annotated using sequence homology. Four blast databases were built with the protein sequences belonging to the *Nicotiana* genus, to the *Solanaceae* family and to *A. thaliana*, downloaded from NCBI-Protein. The sequences were chosen by querying the NCBI-Protein database for the desired species, genus, family or group, including all the listed results. By generating taxonomically confined databases with significance for *N. benthamiana*’s phylogenetic history, we also reduced computational time. The blast databases were built with makeblastdb [57] (makeblastdb -dbtype prot -input_type fasta -parse_seqids). The pre-formatted non-redundant protein and non-redundant nucleotide databases were downloaded from the blast repository. We mapped the gene set encoded protein sequences against these databases with blastp [57] using default parameters and -evalue 0.001 -word_size 3 -outfmt 5 -max_target_seqs 1. The results were filtered keeping only alignments with an E-value ≤ 10e-10, an alignment length ≥ 70 amino acids, sequence identity ≥ 90% and an aligned sequence fraction ≤ 90% (Figs. 7 and 8). The aligned fraction of each sequence was computed with find-best-hit.py [58] which determines how much of the query sequence is covered by mutually compatible high scoring pairs (HSPs), i.e. by non-overlapping HSPs. We first mapped the protein sequences against the *Nicotiana* genus protein database. We then extracted the ones satisfying our criteria, and mapped the remainder against the *Solanaceae* protein database*.* This scheme was repeated, in order, with the *A. thaliana*, non-redundant protein and nucleotide databases. We did not consider as functionally annotated proteins with the descriptors “uncharacterized”, “unknown”, or “hypothetical” or proteins without a match.

### Detection of transgene insertion sites

Raw genomic reads (Additional file 2) were inspected with FastQC [44]. Read trimming was conducted with Trimmomatic [45] (ILLUMINACLIP:TruSeq2-PE.fa:2:30:10 LEADING:3 TRAILING:3 SLIDINGWINDOW:4:15 AVGQUAL:30 MINLEN:36). We mapped ΔXT/FT paired-end genomic reads from a library with a peak insert size of 700 nt (Additional file 2, Barcode LF_DEX_3) against a combined reference that included the Nb-1 draft genome assembly and the two transgene insert sequences (XylT insert, 4,536 nt, FucT insert, 4,768 nt, both including the LB and RB sequences, Additional file 3) using HISAT2 [59] (hisat2 -I 500 -X 775 --no-spliced-alignment --score-min L,-0.6,-0.6 -k 2). We filtered the mapping results keeping primary alignments only (samtools view -F 0×0100). We then extracted read pairs with one mate mapping on an Nb-1 scaffold and the other mate mapping onto a transgene, labeling them as promoter (P) or terminator (T) pairs depending on which region of the transgene they were bridging; connections with < 10 bridging pairs were excluded from further analyses. Local mapping to detect chimeric reads was conducted with bwa [60] (bwa mem -m 5 -k 20 -c 10 -B 6 -O 5,5 -E 3,3 -U 0 -Y -T 20). We filtered the mapping results keeping primary alignments with supplementary alignments using samtools [51] (samtools view -f 2048 -F 0×0100). The junction positions were calculated from the leftmost mapping position, performing the CIGAR operations (BAM format, 6th field). Genomic read coverage per position was computed from the BAM file used for the bridging pairs analysis, using samtools depth [51].

### Gene disruption in ΔXT/FT

To search for fusion transcripts we concatenated the NibSet-1 transcriptome FASTA file with the two transgene cassette sequences (XylT, 840 nt; FucT, 1072 nt; both including sense, intron and antisense fragment). Trimmed transcriptomic reads from ΔXT/FT (Additional file 2) were used (trimming parameters see under “gene prediction”). We cropped the reads to a length of 36 nt to be able to map also most of the reads spanning the fusion junction; using end-to-end alignment those reads would not have aligned to the reference. We mapped the cropped reads with HISAT2 [59] (hisat2 --rdg 5,3 --rfg 5,3 -k 3 --no-spliced-alignment --no-softclip --ignore-quals --score-min L,-0.2,-0.3). We retained only primary alignments from the mapping results (samtools view -F 0×0100). We then extracted read pairs having one mate mapping on the transgene sense/antisense fragment (“insert mate”), and the other mate mapping on g76921 isoforms (“host mate”). The difference between the transgene cassette sequences allowed us to assign the FucT-transgene to this insertion site. Consequently, the XylT-transgene was assigned to the other. Transcriptomic coverage of g76921 was obtained with samtools depth [51], from the mapping scores of wild type and ΔXT/FT transcriptomic reads (Additional file 2).

### ΔXT/FT expression profile

We mapped trimmed transcriptomic reads from ΔXT/FT and wild type with HISAT2 [59] (−-mp 6,2 --rdg 5,3 --rfg 5,3 --score-min L,0.0,-0.2). We filtered the mapping results keeping primary alignments only (samtools view -F 0×0100) and computed read counts with HTSeq [61]. We expected the transcriptomic reads originating from transgenic molecules in ΔXT/FT to map on the regions they were designed to target. Hence, we filtered out read counts in the targeted regions of g31184, g40438, g43728 and g80352 (Additional file 1: Table S5) to avoid a bias in their log-2-fold changes (LFC) estimation caused by transgenic reads. We performed the principal component analysis (PCA) using the tools available within the DESeq2 package [62] and assessed Pearson’s correlation coefficients using the R built-in cor function. We identified a list of differentially expressed genes (DEGs) with DESeq2 [62]. We kept only DEGs with an average mean coverage of at least 10 across replicates and conditions. We then tested for LFC ≥ 0.5 at α < 0.05. For the resulting DEGs, we computed the TPM in each replicate and condition. We applied a sample-specific TPM threshold to consider a gene as expressed: we obtained the threshold via the conversion formula TPM_i_ = (FPKM_i_ / sum_j_(FPKM_j_))*10^6^ [63] using FPKM_i_ = 1. Only genes with TPM equal or above threshold in at least one condition were kept. The thresholds used were 3.41, 3.43, 3.45 and 3.45 for samples LF_DEX_1, LF_DEX_2, LF_NIB_1 and LF_NIB_2 respectively. Function and GO terms for the identified DEGs were obtained by querying the online Eudicots database of Blast (taxid: 71240) [64] and interPro [32].

### qPCR

Total RNA was reverse transcribed using the iScript cDNA Synthesis kit (Bio-Rad, Hercules, CA, USA). Real-time qPCR was performed in triplicate using the GoTaq qPCR master mix (Promega, Madison, WI, USA). Serine/threonine protein phosphatase 2A (PP2A) expression was used for normalization of qPCR data. Three independent biological replicates were used and mean values ± standard deviation are given, together with a two-tailed *p*-value representing the significance (Additional file 1: Figure S10). Primers used in this study are listed in Additional file 1: Table S6.

### Genomic variants

Trimmed genomic sequencing reads (Additional file 2, codes LF_DEX_3, LF_NIB_3, trimming parameters see “Detection of the transgene insertion sites” methods section) were aligned to the Nb-1 draft genome assembly with Bowtie2 [65] (−-sensitive --mp 6 --rdg 5,3 --rfg 5,3 --score-min L,-0.6,-0.6), setting a minimum and maximum insert size of 500 bp and 775 bp, respectively (−I 500 -X 775), which had been estimated by mapping a subset of 50,000 read pairs of each library (Additional file 1: Figure S11) against Nb-1. The used mapping parameters allowed a maximum of 12 mismatches, a maximum gap length of 23, or a combination of the two. The mapping returned a 21-fold coverage for ΔXT/FT and a 26-fold coverage for WT. The mapping results were then sorted by genomic coordinates keeping only the primary alignments (samtools view -F 0×0100). The raw call set was obtained with samtools mpileup [66] (call -f GQ,GP -v -m). Results were filtered with a combination of custom scripts. We required an average mapping quality and a calling quality of 20 (Phred score), a minimum coverage of 4, a maximum coverage of 30 for ΔXT/FT and of 38 for WT, a maximum fraction of reads with 0-mapping quality of 10% and a minimum number of reads per strand of 1. The filtered set of variants was compared with variants called with the same pipeline using sequencing reads isogenic to the plant used for the draft genome assembly (provided by A. Bombarely, Latham Hall, Virginia Tech, Blacksburg, VA, USA), to remove false calls due to consensus errors in the assembled genome. Isogenic sequencing reads were filtered with Trimmomatic using the following parameters: LEADING:25 TRAILING:25 SLIDINGWINDOW:4:20 AVGQUAL:35 MINLEN:40. Variants shared between ΔXT/FT and WT, and variants unique to either ΔXT/FT or WT were extracted with the bedtools “intersect” function [55].

The functional impact of variants annotated within coding regions of ΔXT/FT was assessed with SnpEff [37], identifying low, moderate and high impact variants as defined in the program documentation (http://snpeff.sourceforge.net/SnpEff_manual.html#eff). We performed a GO term analysis for the genes containing a variant with high impact. This analysis was conducted with InterproScan [67].

### Transcriptomic variants

Quality-filtered reads from *N. benthamiana* samples ΔXT/FT and WT, *N. benthamiana* samples from research institutions other than BOKU (SRR651957, SRR2976595, ERR219219, SRR1043177, SRR2085476), *N. sylvestris* (ERR274390) and *A. thaliana* (SRR6236990, SRR5195552, SRR3223423, SRR3928353, SRR5040365, DRR070513) were cropped to a length of 48 nt. *N. benthamiana* and *N. sylvestris* reads were downsampled to 14 million reads, while *A. thaliana* reads were downsampled to 8.5 million reads. Reads were mapped against the Nb-1 draft genome assembly [18] with HISAT2 [59] (−-trim5 5 --no-softclip --mp 6,6 –rdg 5,3 –rfg 5,3 --score-min L,2.4,-0.3). Only primary alignments (samtools view -F 0×0100) mapping within CDS regions (i.e. excluding UTRs) were retained, if they had at least one mismatch difference between primary and secondary alignment; PCR duplicates were removed with Picard (http://Broadinstitute.Github.Io/Picard). Coverage was extracted with samtools depth [51]. Candidate variants were obtained through samtools mpileup [66] (−t DP,AD,ADF,ADR,SP,DP4) and bcftools call [68] (−f GQ,GP -v -m). We excluded: positions within 10 nt from an indel; indels within 100 nt from each other; clusters of 3 SNVs within 10 nt (all likely alignment artifacts). We requested a minimum base quality of 20, a minimum average mapping quality of 20, a minimum coverage of 4x, a minimum fraction of 0.1 (10%) reads with 0-mapping quality (MQ0F), a minimum fraction of 0.9 (90%) reads showing the alternative allele at each variant position. The thirteen different assemblies of *A. thaliana* were downloaded from the 1001genomes website [42]. For each we determined the coding regions by mapping the TAIR10 [41] *A. thaliana* transcript sequences against the assemblies with GMAP [69] (−f gff3_gene --min-identity 0.95); CDS lines from the resulting GFF3 file were piped to bedtools merge [55] to generate a non-redundant representation of coding positions. Reads from the “Netherlands” sample (lab-grown ecotype Col-0) were mapped against each of the assemblies, and variants were called using the same programs and criteria as used for the six Col-0 accessions.

## Additional files

Additional file 1: **Table S1.** Transposable elements within the *N. benthamiana* reference genome. **Table S2.** BUSCO analysis to assess gene set completeness. **Table S3.** Number of sequences, database total length of each constructed database. **Table S4.** Normalized counts for target genes of the FucT and XylT transgenes. **Table S5.** Regions of FucT1, FucT-pseudogene, XylT1, XylT2 targeted by transgenes. **Table S6.** Primer sequences for qPCR. **Table S7.** Potential off-target effects of FucT-transgene and XylT-transgene. **Table S8.** Pearson's correlation between normalized counts of the four mRNA-seq samples. **Figure S1.** Gene models obtained by mapping sequences of FucT and XylT genes onto the Nb-1 draft genome assembly. **Figure S2.** Genomic coverage of transgenes within the ΔXT/FT genome. **Figure S3.** Genomic coverage in ΔXT/FT and wild type on scaffold Niben101Scf03674 and Niben101Scf03823. **Figure S4.** Re-assembly of region of insertion of XylT transgene. **Figure S5.** Alignment between scaffolds containing genes g76921 and g54961. **Figure S6.** Protein sequence alignment between genes g76921 and g54961. **Figure S7.** Multiple sequence alignment of g76921 and g54961. **Figure S8.** Folding of *N. benthamiana* proteins encoded by g76921 and g54961. **Figure S9.** Principal component analysis (PCA) on normalized read counts. **Figure S10.** ΔΔCT values and TPM for differentially expressed genes. **Figure S11.** Insert size estimation of ΔXT/FT, WT genomic sequencing libraries. (PDF 8074 kb)

Additional file 2: High-throughput sequencing data. (ODS 25 kb)

Additional file 3: Sequences of the constructs used for the generation of ΔXT/FT. (PDF 168 kb)

Additional file 4: Differential gene expression analysis results. (TXT 4526 kb)

Additional file 5: High impact variants. (TXT 441 kb)

## Data Availability

*N. benthamiana* genomic and transcriptomic data from BOKU wild type and ΔXT/FT lines are available under SRA Bioproject PRJNA481441, accession numbers SRR7540369, SRR7540370, SRR7540371, SRR7540372, SRR7540367, SRR7540368. Gene models, predicted protein sequences and gff files are available at http://bioinformatics.boku.ac.at/NicBenth/Download/. The sequence of the plasmid used in the generation of ΔXT/FT is provided as Additional file 3. *N. benthamiana* seeds can be obtained from one of the authors (RS). The transgenic line ΔXT/FT is available for academic research upon signature of a Material Transfer Agreement.
