# Supplementary material for: Genome and transcriptome characterization of the glycoengineered Nicotiana benthamiana line ΔXT/FT
Source: BMC Genomics. 2019 Jul 19;20:594. doi: 10.1186/s12864-019-5960-2 (PMC6642603; doi:10.1186/s12864-019-5960-2)
Supplement: Supplementary file 1 — Table S1. Transposable elements within the N. benthamiana reference genome. Table S2. BUSCO analysis to assess gene set completeness. Table S3. Number of sequences, database total length of each constructed database. Table S4. Normalized counts for target genes of the FucT and XylT transgenes. Table S5. Regions of FucT1, FucT-pseudogene, XylT1, XylT2 targeted by transgenes. Table S6. Primer sequences for qPCR. Table S7. Potential off-target effects of FucT-transgene and XylT-transgene. Table S8. Pearson's correlation between normalized counts of the four mRNA-seq samples. Figure S1. Gene models obtained by mapping sequences of FucT and XylT genes onto the Nb-1 draft genome assembly. Figure S2. Genomic coverage of transgenes within the ΔXT/FT genome. Figure S3. Genomic coverage in ΔXT/FT and wild type on scaffold Niben101Scf03674 and Niben101Scf03823. Figure S4. Re-assembly of region of insertion of XylT transgene. Figure S5. Alignment between scaffolds containing genes g76921 and g54961. Figure S6. Protein sequence alignment between genes g76921 and g54961. Figure S7. Multiple sequence alignment of g76921 and g54961. Figure S8. Folding of N. benthamiana proteins encoded by g76921 and g54961. Figure S9. Principal component analysis (PCA) on normalized read counts. Figure S10. ΔΔCT values and TPM for differentially expressed genes. Figure S11. Insert size estimation of ΔXT/FT, WT genomic sequencing libraries. (PDF 8074 kb) [file 12864_2019_5960_MOESM1_ESM.pdf]

Supplementary Material

1 index

Supplementary Text:

2 *ΔXT/FT XylT-transgene insertion site assembly*
2 *Possible complementation of TFIID subunit 12-like isoform X1 gene disruption in ΔXT/FT*
3 *ΔXT/FT transgene targets and off-targets*
4 *Transgene-targeted regions*
4 *Genomic variant calling with assembled contigs*

5 Supplementary methods

7 Supplementary Tables S1-S8

11 Supplementary Figures S1-S11

20 Supplementary Table Legends

21 Supplementary Figure Legends

23 Supplementary References

ΔXT/FT XylT-transgene insertion site assembly

To get more information on the sequences flanking the transgene ends, we re-assembled the insertion region using ΔXT/FT genomic Illumina sequencing reads. We assembled the transgene promoter with 570 bp of flanking genomic sequence, and the terminator together with 1451 bp of flanking genomic sequence. The flanking regions corresponded to the sequences where bridging pairs mapped with their host mate (Figure 2, panel "b"). We mapped genomic paired-end reads on the reassembled version of the region, to detect candidate links to other contigs. We formed a connection upon detection of at least 10 bridging pairs. Each connected contig was then mapped against the Nb-1 draft genome assembly to identify its position with respect to the identified junctions. Connections were evaluated manually. The proposed structure is the one which required the least number of assumptions (Supplementary Figure S4).

Possible complementation of TFIID subunit 12-like isoform X1 gene disruption in ΔXT/FT

Considering that *N. benthamiana* is an ancient tetraploid, we assessed whether other copies of a related TFIID subunit 12-like isoform X1 gene could compensate for a loss of function at the g76921 locus. We aligned the protein sequence of g76921 against the NibSet-1 protein sequences, and detected a match at 90% minimum identity with g54961, also annotated as TFIID subunit 12-like isoform X1. To confirm that the two genes are located on different chromosomes and do not represent the same gene separately assembled twice, we aligned against each other the two scaffolds that contain the genes g76921 and g54961, respectively (Supplementary Figure S5). The alignment showed that these scaffolds do not represent separately assembled haplotypes.
We then checked whether g54961 is an active gene. We observed high expression (FPKM=9.0, TPM=13.9) in leaf tissue and did not detect differential expression (LFC = -0.02) between ΔXT/FT and WT; we therefore concluded that g54961 is expressed in ΔXT/FT as it is in WT. We then performed a pairwise global alignment between the g76921.t1 and g54961.t1 protein sequences (Supplementary Figure S6). The functional domain that is specific to the TFIID 20 kDa protein family (PF03847) of Pfam (70) was found with 57-60% sequence identity in the two sequences (positions 394-461 in g76921.t1, positions 408-475 in g54961.t1); the domain matched with bitscore 102.0 and E-Value 1.7e-29 on g76921.t1, with bitscore 106.7 and E-Value 5.8e-31 on g54961.t1.
We then assessed the level of conservation between TFIID subunit 12-like isoform X1 proteins across species. We downloaded the protein sequences annotated as such in the NCBI-protein database and performed a multiple sequence alignment including the two *N. benthamiana* proteins g76921.t1 and

g54961.t1. The results indicate that these protein sequences are not well conserved, in contrast to the C-terminal TFIID-specific well-conserved functional domain (Supplementary Figure S7). We finally performed a secondary structure prediction (71) that showed a strong alpha-helix propensity in this region. Using Swiss-MODEL (72,73) we observed the predicted fold for both g76921.t1 and g54961.t1, demonstrating that the folding of both proteins is essentially equal (Supplementary Figure S8). We concluded that the disruption of g76921 likely has no impact on the plant and that g54961 could potentially buffer the loss of function of the disrupted gene.

ΔXT/FT transgene targets and off-targets

The two transgenes were designed to target the core α1,3-fucosyltransferases (FucT) and β1,2-xylosyltransferases (XylT) of *N. benthamiana*. For the FucT gene family, five copies have been reported in *N. benthamiana* (29), with two of them characterized in a previous study (8). The two copies characterized in Strasser et al. (2008) are the ones targeted by the FucT-transgene that was inserted in the ΔXT/FT genome; they are named FucT1 and FucT-pseudogene (FucT-p), and the latter is not encoding a functional protein. They are highly similar in terms of sequence and a single transgene was used to target both of them. The other transgene was used to target the two documented XylT genes (XylT1 and XylT2). The mRNA sequences of these genes are publicly available in Genbank (EF562630.1, EF562631.1, EF562628.1, EF562629.1), and were obtained by sequencing cDNA from the wild type genotype used for the generation of ΔXT/FT. Based on sequence identity, we identified the four corresponding transcripts within gene set NibSet-1 (FucT1: g31184.t1, FucT-p: g80352.t1, XylT: g43728.t1, XylT: g40438.t1). NibSet-1 and NCBI gene sequences matched with ≥ 99% sequence identity. We concluded that these were the four targeted transcripts in ΔXT/FT. Based on this and other observations, we replaced the NibSet-1 sequences of these four genes by their manually curated Genbank counterparts.
We then assessed whether the transgenes could have an off-target effect on other transcripts. The hairpin RNAs produced by the transgenes are processed into short RNA molecules with a length of 19-30 nt (74). The processed RNA molecules interfere with target transcripts either as siRNAs, mediating cleavage of the target (75), or as miRNAs through mechanisms that prevent protein translation (76). Optimal criteria for gene silencing have been assessed multiple times, leading to different conclusions (77–80). In any case, the silencing efficiency correlates with the number of short RNAs that have 100% sequence identity with their target. We therefore generated all possible k-mers of length 19 nt, 21 nt, 25 nt and 30 nt, respectively, from the sequences of the two transgene fragments in sense orientation (Supplementary Table S7) and mapped each k-mer set against the

NibSet-1 transcripts with BLAT, requiring a sequence identity of 100% and allowing multiple hits for each k-mer. With 19-mers we observed two putative off-target transcripts (g3481.t1 and g36277.t1); both were annotated as “PREDICTED: glycoprotein 3-alpha-L-fucosyltransferase A-like” in our functional annotation, and correspond to the FucT3 and FucT5 genes reported in Jansing et al. (2018), respectively. These two were not found among the results obtained with the longest k-mers (30-nt); also, the difference in the number of matching k-mers between the targeted genes and the two putative off-targets was large (Supplementary Table S7). This is in line with the findings in Jansing et al. (2018), as in such study, another transgene had to be generated in order to target the additionally characterized FucT copies. We concluded that there likely are no off-target effects with the two transgenes used.

Transgene-targeted regions

We determined regions of the transcripts that are targeted by the transgenes. We mapped the sense fragment of the transgenes (FucT-transgene=426 nt, XylT-transgene=314 nt, both excluding the leading 'TCTAGA' XbaI restriction site, see Supplementary File 2) against the four NCBI transcript sequences and against their four NibSet-1 counterparts (Supplementary Table S5) using Blast. We observed high identity matches with FucT1, FucT-p and XylT1 for both the NCBI and the NibSet-1 version, but no match with XylT2 in the NibSet-1 (i.e. Augustus) version (g40438.t1, Supplementary Table S5). We therefore assessed the differences between the NibSet-1 and the NCBI sequences. We first obtained gene models in GFF3 format from the NCBI sequences by mapping them on the Nb-1 draft genome assembly with GMAP. We then visualized them together with the NibSet-1 models in a genome browser and we assessed the differences. The models showed overall compatible exon-intron junctions between NibSet-1 and NCBI; the differences resided mostly in the beginning or the end of each model (Supplementary Figure S1). In g40438, the transgene-targeted region was found in a part of the 3' end that is not present in the NibSet-1 model (Supplementary Figure S1, panel “d”). For the other three targeted genes, the regions targeted by the transgene were included in both models.

Genomic variant calling with assembled contigs

The ΔXT/FT genomic paired-end reads used for variant calling were assembled using SOAP-denovo2, with k-mer size 80. We generated 697,264 unscaffolded contigs of at least 1,000 bp length, with a contig N50 of 2,904 bp (total length of assembly with contigs ≥ 1000 bp: 1.92 Gbp). We mapped contigs of at least 1000 bp against the Nb-1 draft genome assembly using nucmer (81) requesting a

minimum alignment length of 1000 nt and a minimum alignment seed of 30 nt. Given the high repeat content and the allopolyploidy of the *N. benthamiana* genome, we allowed nucmer to extend only seeds that were unique both in the genome and in the mapped contig (--mum option). 691,656 contigs (99.2%) found a mapping location of which 604,358 were mapped uniquely. We extracted the mismatching positions from the filtered mapping scores and generated a VCF-formatted file containing candidate SNVs. We note that a higher number of variants is usually recovered with this method, as a fully sensitive alignment of a long sequence will detect many SNVs that are missed by short reads. When asking a contig to map with a minimum identity of 75% and at least 10% of its sequence mapping uniquely, we obtained 2743 SNVs/Mbp over the whole genome and 601 SNVs/Mbp over coding sequences (CDS). In both cases we divided the SNVs by the positions covered by the contigs.

Supplementary Methods

Transgene insertion site assembly

To assemble the insertion region in scaffold Niben101Scf03823, we extracted matching Illumina reads from ΔXT/FT with samtools view. With bedtools bamtofastq (69) we generated paired FASTQ files based on the mapping scores recorded in the BAM files. Unpaired reads were saved in a separate FASTQ file. The generated FASTQ files were used as input for SOAP-denovo2 (82) (all -w -L 450 -K 57 -d 1 -D 1 -F -w -G 70 -L 200 -c 5 -C 35 -b 1.75 -M 3). We mapped the known promoter/terminator sequences against the generated assembly with BLAT (-minIdentity=95), identifying the scaffolds corresponding to the promoter or to the terminator sequence, respectively. We mapped these scaffolds against the Nb-1 assembly with BLAT (-minIdentity=95) to identify the location of the co-assembled flanking regions. To extend the co-assembled flanking regions we mapped the genomic paired-end reads of ΔXT/FT against the generated assembly using the same pipeline used for the bridging pairs analysis.

Analysis of protein structure using web resources

To find genes similar to g76921 within the *N. benthamiana* genome, we aligned the g76921 protein sequence against the NibSet-1 predicted protein sequences with BLAT (-prot -minIdentity=90). Pairwise alignment between protein sequences was done on the EMBOSS Needle web server (83). The multiple sequence alignment was performed on the MUSCLE web server (84). The secondary structure prediction was done according to the Chou & Fasman algorithm using the APSSP web

server, unpublished but referenced in CAFASP3 (85). The protein fold prediction was obtained on the Swiss-MODEL web server. Genomic sequence comparison between scaffolds were performed using nucmer (-b 200 -c 65 --delta -g 1000 -l 10000).

ΔXT/FT transgene targets and possible off-target effects

We mapped the FucT1, FucT-pseudogene, XylT1 and XylT2 mRNA sequences deposited in Genbank (EF562630.1, EF562631.1, EF562628.1, EF562629.1) against NibSet-1 with BLAT (-minIdentity=80). We mapped the sense fragments of the transgenes (FucT-transgene, 426 nt; XylT-Transgene, 314 nt; see Supplementary File 2) against the mRNA sequences from Genbank (see above) and against the corresponding NibSet-1 genes using Blast (match/mismatch 1/-2, word size 28). We generated gene models for the targeted transcripts with GMAP (-f gff3_gene --min-identity 0.95). We generated all the possible k-mers of sizes 19, 21, 25, 30 nt with a custom python script using the transgenes' sense fragments as templates (426 nt for FucT, 314 nt for XylT). We mapped the 19, 21, 25 and 30-nt k-mers against NibSet-1 with BLAT (-minIdentity=100 -tileSize=8 -minMatch=2 -stepSize=1 -minScore=16).

Variant determination based on alignment of assembled contigs against the Nb-1 reference

ΔXT/FT Illumina genomic reads corresponding to 33-fold genomic coverage were assembled with SOAP-denovo2 (-L 500 -K 80 -M 3). The unscaffolded contigs resulting from this assembly were mapped against the Nb-1 draft genome assembly with nucmer (--mum --breaklen=500 --mincluster=60 --maxgap=500 --minmatch=30 --minalign=1000). Nucmer's module "delta-filter" was used to filter alignment results (-i 75.0 -l 1000 -u 10.0). Coordinates from the filtered alignment scores were generated with the "show-coords" module (-T) and were rendered in a non-redundant BED formatted file with bedtools merge. Contigs overlapping coding regions were extracted using bedtools intersect (-u). SNVs were extracted from the filtered alignment scores using the module "show-snps" (-T -r -I) and piped into the mummer2Vcf.pl script (https://github.com/douglasgscofield/bioinfo/blob/master/scripts/mummer2Vcf.pl) to generate a pseudo-VCF formatted file. Variants within coding regions were extracted with bcftools view, providing a non-redundant list of annotated CDS as a condition (-R).

Supplementary Tables

Annotated transposable elements			
Element	Number of elements	Length occupied [bp]	% of sequence occupied
SINEs	85,370	11,479,130	0.46%
LINEs	85,868	68,304,057	2.74%
LTR	1,274,563	1,388,723,157	55.79%
DNA elements	95,760	38,727,508	1.56%
Unclassified	8,782	2,954,159	0.12%
TOTAL	1,550,343	1,510,188,011	60.67%

Supplementary Table S1

	NibSet-1	SGN
Complete Single-copy genes	209	210
Complete Duplicated genes	719	698
Fragmented genes	9	24
% complete + fragmented	98.0%	97.5%
Missing genes	19	24
Total gene groups searched	956	956

Supplementary Table S2

	Number of sequences	Total length [bp]
Nicotiana genus	273,385	113,965,582
Solanaceae family	504,862	205,489,684
A. thaliana	48,315	20,855,795
BLAST Eudicots	1,668,278	744,348,453
BLAST nr protein	106,376,657	38,985,428,197
BLAST nr nucleotide	37,848,925	123,933,400,280

Supplementary Table S3

Gene	Name	Scaffold	Start	End	ΔXT/FT 1 counts	ΔXT/FT 2 counts	WT 1 counts	WT 2 counts
g31184	FucT1	Niben101Scf01272	406	7004	10.83	14.79	21.97	19.23
g80352	FucT-pseudogene	Niben101Scf02631	1984	8357	10.83	12.52	36.03	40.30
g43728	XylT1	Niben101Scf04551	243896	248041	41.15	56.89	96.66	99.83
g40438	XylT2	Niben101Scf04205	352063	356011	24.91	38.68	120.38	103.50

Supplementary Table S4

Gene	Transcript version	Source	Transgene	Match Length	Matches	Mis-matches	Start on transcript	End on transcript
FucT1	EF562630.1	NCBI	FucT	417	415	2	629	1045
	g31184.t1	Augustus	FucT	417	415	2	759	1175
FucT pseudogene	EF562631.1	NCBI	FucT	417	405	12	629	1045
	g80352.t1	Augustus	FucT	417	403	14	1608	1195
XylT1	EF562628.1	NCBI	XylT	310	303	7	1257	1556
	g43728.t1	Augustus	XylT	310	302	8	2117	2426
XylT2	EF562629.1	NCBI	XylT	310	309	1	1240	1549
	g40438.t1	Augustus	XylT	-	-	-	-	-

Supplementary Table S5

Gene	Primer Name	Sequence (5’-3’)
PP2A	Nb_PP2A_Q1F	GACCCTGATGTTGATGTTCGCT
	Nb_PP2A_Q2R	GAGGGATTTGAAGAGAGATTTC
g10744	NbA_Q1F	AATGGTGTTCAGTTTATGGATGC
	NbA_Q2R	TTCAAGAAATACCGGACCAGG
g25290	NbB_Q1F	CAACATTTTCAGGGACACGC
	NbB_Q2R	CAAGCATCCAGTTGTGTCATG
g29021	NbC_Q3F	TGGTACTCCAGGGAAAATGC
	NbC_Q4R	TTCTCACCACCAGGCTTACC
g40387	NbD_Q3F	GCTGGAGTTCCTGCAGATTC
	NbD_Q4R	TGCAAGTTTGTCCACCAAAA
g67787	NbE_Q3F	ATCCAGTTCTTGACGCACCT
	NbE_Q4R	AGTGACGAGGCCATTGAGTT
g76591	NbF_Q3F	ATTATCCTGCTTGGGGGTTC
	NbF_Q4R	TGATACCCTGGATTCCTTGC
g9149	NbG_Q3F	GCGAGACAATGCAACTACGA
	NbG_Q4R	AATTGAAAGGGCCACAGATG
g16390	NbH_Q1F	CCATACCCCTCTCAAGGTCA
	NbH_Q2R	GATAGAGGGATCGCAGCAAC
g21681	NbI_Q1F	GGAGCTTGCACTTTCTTTGG
	NbI_Q2R	CCTTTGCCCACTCTTCTCAG
g29742	NbJ_Q1F	TGGCATTCCCCGTATTCCAT
	NbJ_Q2R	GGCAAATTTCTCCACTGGCA
g45032	NbK_Q3F	GCTGGACAGGATCAGTACGA
	NbK_Q4R	GCTTCCTTTTGTGGCATTGC
g55101	NbL_Q3F	TCATCGAAGAGGAAGTGTCATTTTG
	NbL_Q4R	GATCCTCTGTCCATTCTTCCTGTTTAT
g76921	NbM_Q3F	CACCTCCACCACCTTCGTCCTC
	NbM_Q4R	GCGGTTGCGGCTGTAGTGGTA
g90787	NbN_Q1F	CTTGTGTGAACCCTGAGAGC
	NbN_Q2R	CTTTCGGTTGTGAGGTGCAA

Supplementary Table S6

a

	Produced k-mers			
Source of k-mers	19-mers	21-mers	25-mers	30-mers
FucT-transgene	407	405	401	396
XylT-transgene	295	293	289	284

b

					Mapped k-mers			
Transcript	Name	Scaffold	Start	End	19-mers	21-mers	25-mers	30-mers
g31184.t1	FucT1	Niben101Scf01272	406	7004	370	366	358	348
g80352.t1	FucT-pseudogene	Niben101Scf02631	1984	8357	213	199	171	143
g43728.t1	XylT1	Niben101Scf04551	243896	248041	183	173	153	128
g40438.t1	XylT2	Niben101Scf04205	352063	356011	273	269	261	251
g3481.t1	3-alpha-L-fucosyltransferase A-like	Niben101Scf05494	208147	214675	22	16	8	-
g36277.t1	3-alpha-L-fucosyltransferase A-like	Niben101Scf05447	449846	451560	4	-	-	-

Supplementary Table S7

	WT 1	WT 2	ΔXT/FT 1	ΔXT/FT 2
WT 1	-	-	-	-
WT 2	0.989	-	-	-
ΔXT/FT 1	0.997	0.989	-	-
ΔXT/FT 2	0.994	0.977	0.993	-

Supplementary Table S8

Supplementary Figures

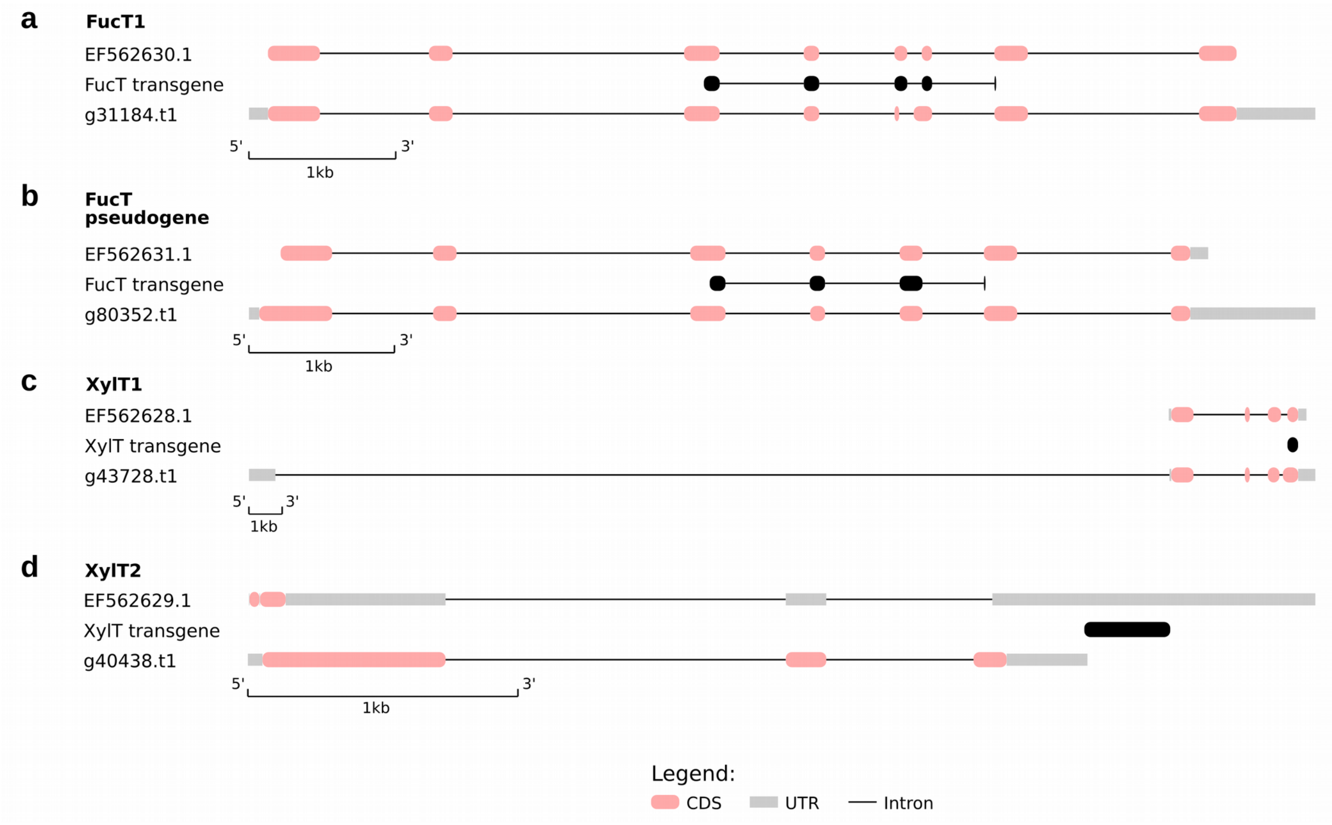

Supplementary Figure S1

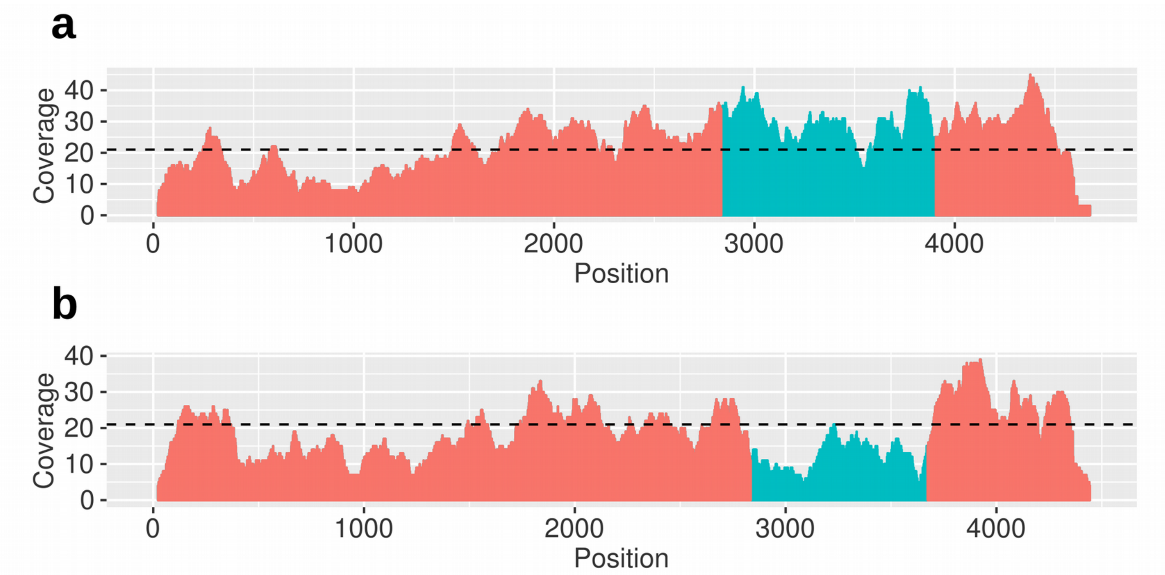

Supplementary Figure S2

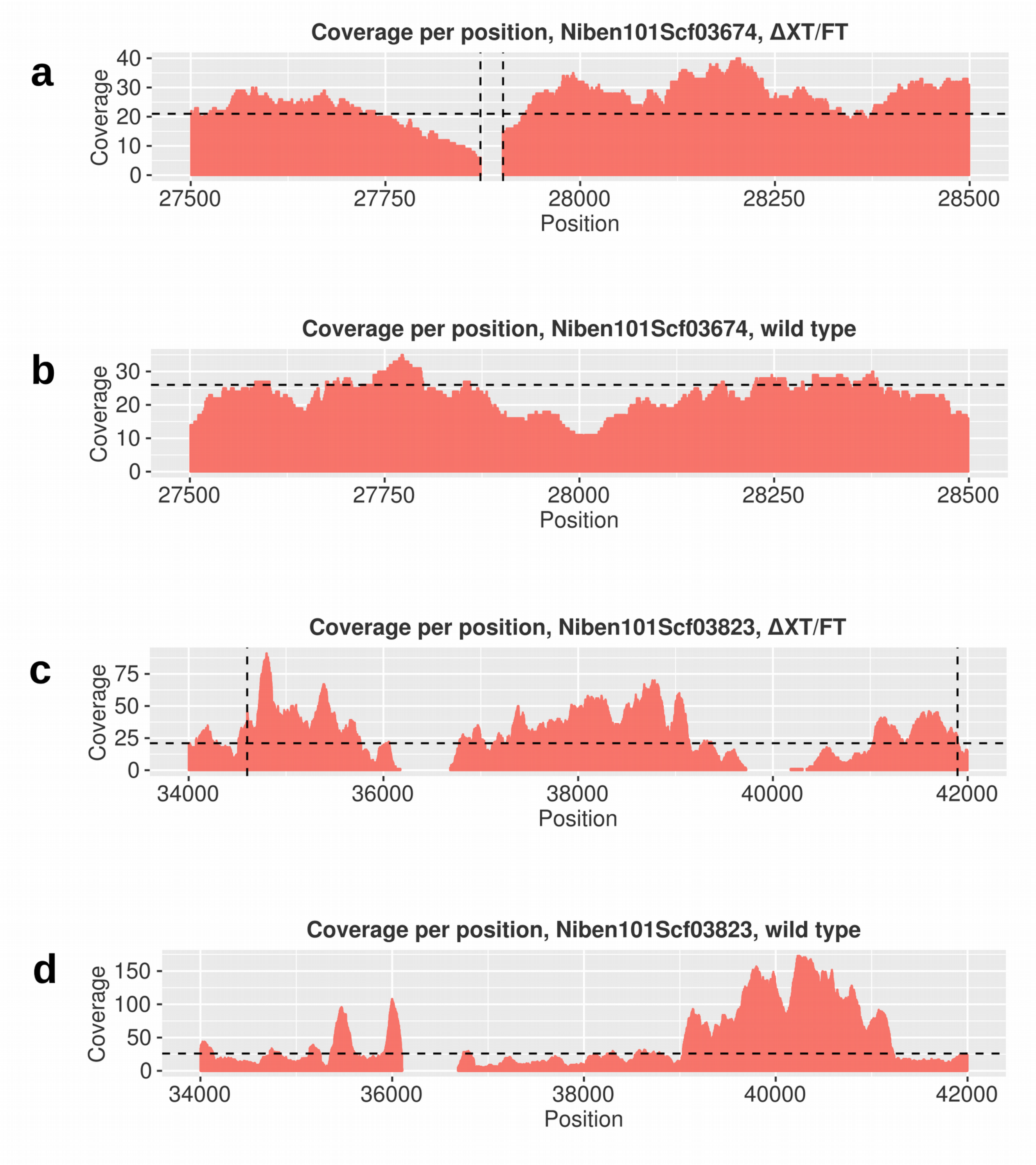

Supplementary Figure S3

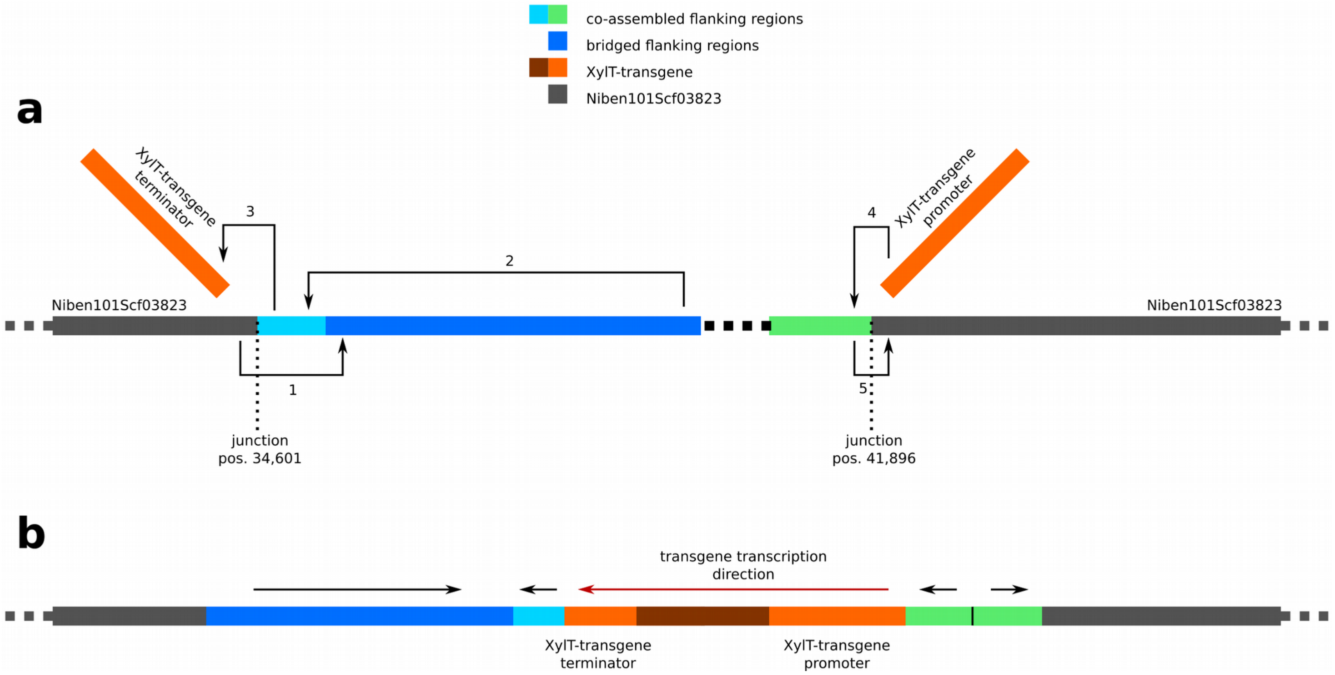

Supplementary Figure S4

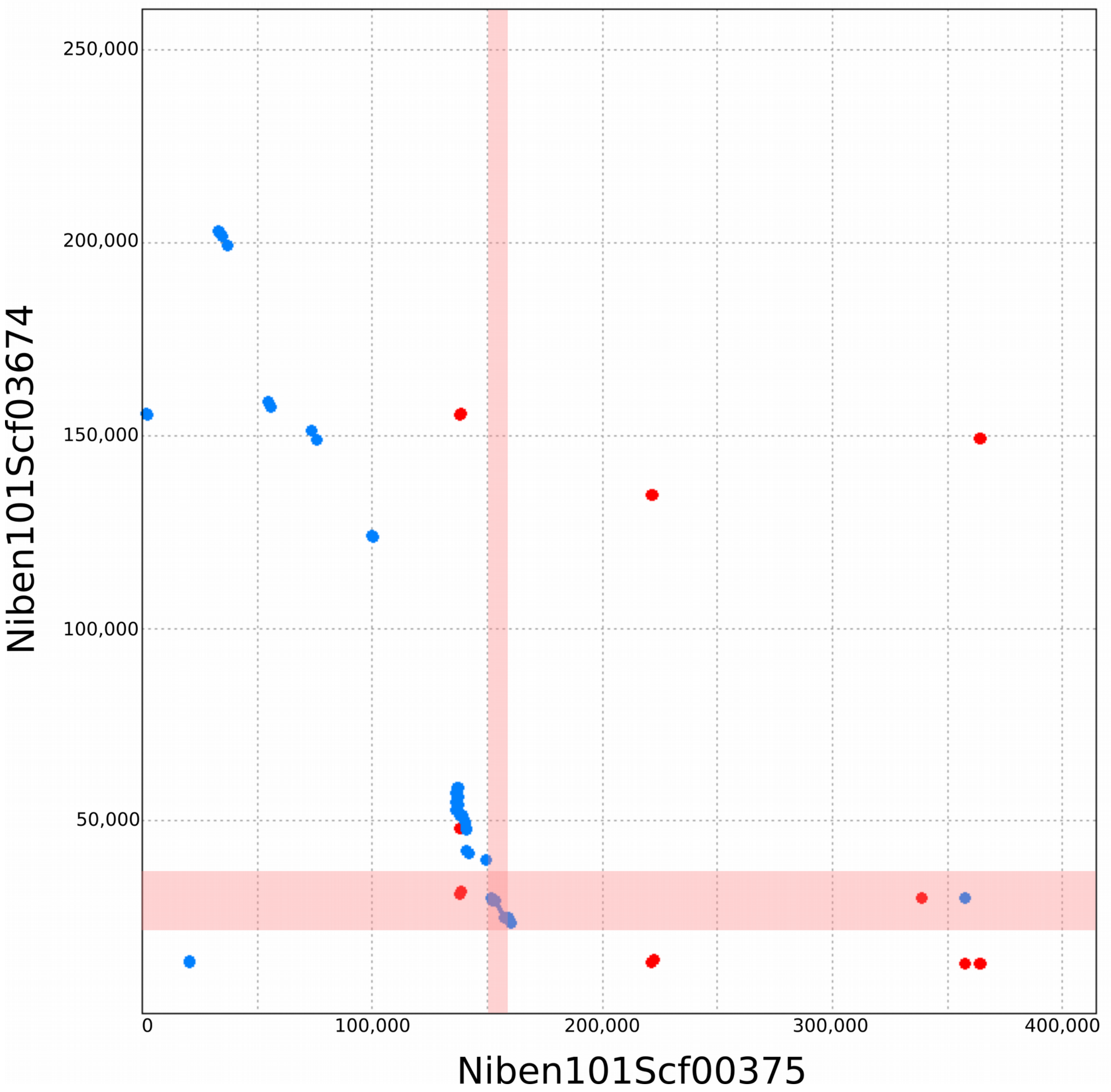

Supplementary Figure S5

```
g54961.t1          1 MEQTQ----PPPSPTPSPSTSTSQPTEQQQQQLQPPSPPPPP-SSAPATS     45
                     |||||    |||:|||||||||||||||.||||||.|||||| |||::||
g76921.t1          1 MEQTQQPPTPPPPPTPSPSTSTSQPTEQLQQQLQPQSPPPPPSSSAATTS     50

g54961.t1         46 QLPSTTTTTTSVVQSQSPQNLNPTTTTITATTTAATAAATSTQQQNPLTP     95
                     ||||:|||||||||||||||.|||||||||||||||||||||||||||||
g76921.t1         51 QLPSSTTTTTSVVQSQSPQNQNPTTTTITATTTAATAAATSTQQQNPLTP    100

g54961.t1         96 TLQNAQTRQPFNRPWQQPSPFQHFSLPPPPPPPPPH--SSSSSSITSSSS    143
                     ||||||||||||||||||||||||||||||||||||  ||||||||||||
g76921.t1        101 TLQNAQTRQPFNRPWQQPSPFQHFSLPPPPPPPPPHSSSSSSSSITSSSS    150

g54961.t1        144 SVSMQNPRGVGGMAVGVPAHHPPTSFSSLTPPPPSFGQQFGRNLPDSSAP    193
                     |||||:|||||||::|||||||:|||||||||:|||||||||||||.|||
g76921.t1        151 SVSMQSPRGVGGMGMGVPAHHPSTSFSSLTPPSPSFGQQFGRNLPDFSAP    200

g54961.t1        194 ISTTSQVRQPIQGMHGMGMMGSLGSTSPMRPAGVPQQLRPVASSLRPQTS    243
                     .||.|||||||||:||.||||||||||.||||||||||||.|||||||||
g76921.t1        201 TSTPSQVRQPIQGLHGTGMMGSLGSTSLMRPAGVPQQLRPFASSLRPQTS    250

g54961.t1        244 IVSQSAATQNYQGHGMLRVQSVGLPSSQLHTMSQSPRAQNQPWLSSGAQG    293
                     |.||||.|||:|||||.|                     |||||||||||
g76921.t1        251 IGSQSAVTQNFQGHGMPR---------------------NQPWLSSGAQG    279

g54961.t1        294 KPALPTPSLRPQISPQTLHQRSHILSQHQHTVTTSSSAQQSQLSTSSLSQ    343
                     ||:|||||||||||||||||||||||||||.||||||||||||||||.||
g76921.t1        280 KPPLPTPSLRPQISPQTLHQRSHILSQHQHIVTTSSSAQQSQLSTSSQSQ    329

g54961.t1        344 DHLGQQMPPSRIPQSLSNQPLARGQGLGVQRPSSHALMQSATVKPGPPSM    393
                     |||||||.||||:||||||||||||||||||||||||||||||||||||.
g76921.t1        330 DHLGQQMRPSRISQSLSNQPLARGQGLGVQRPSSHALMQSATVKPGPPSK    379

g54961.t1        394 ATTLETEEPCTRILSKRSIQEILTQIDPSEKLDTEVEDVLVDIAEEFVES    443
                     .||||||||||||||||||||||||||||||||:||||||||||||||||
g76921.t1        380 DTTLETEEPCTRILSKRSIQEILTQIDPSEKLDAEVEDVLVDIAEEFVES    429

g54961.t1        444 ITTFGCSLAKHRKSTTLEAKDILLHLERNWNMTLPGFSGDEIRTYKKPFT    493
                     |:|||||||||:||.|||||||||||||||||||||||||||||||||
g76921.t1        430 IATFGCSLAKHQKSNTLEAKDILLHLERNWNMTLPGFSGDEIRTYKKP--    477

g54961.t1        494 SDIHKERIAAIKRSALVAEMTNAKGSAQAGGGMKGHLAKGPACILGSPNA    543
                               ||:|||||||||||||||||||||||||||||.|||||||
g76921.t1        478 ----------IKKSALVAEMTNAKGSAQAGGGMKGHLAKGPANILGSPNA    517

g54961.t1        544 KT    545
                     ||
g76921.t1        518 KT    519
```

Supplementary Figure S6

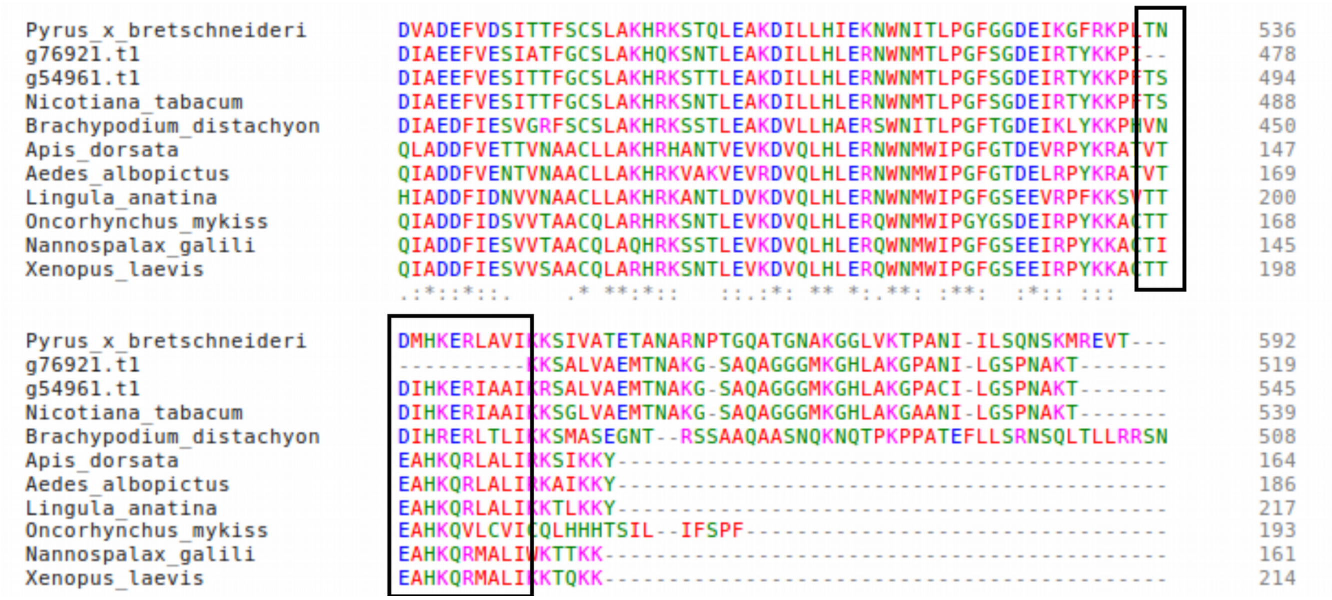

Supplementary Figure S7

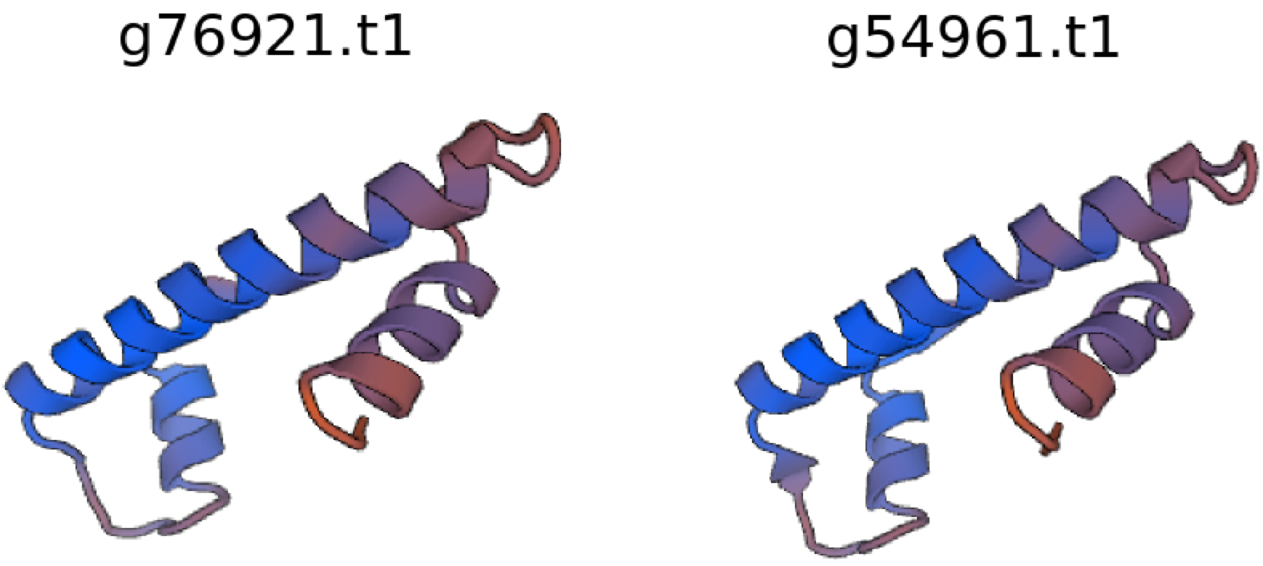

Supplementary Figure S8

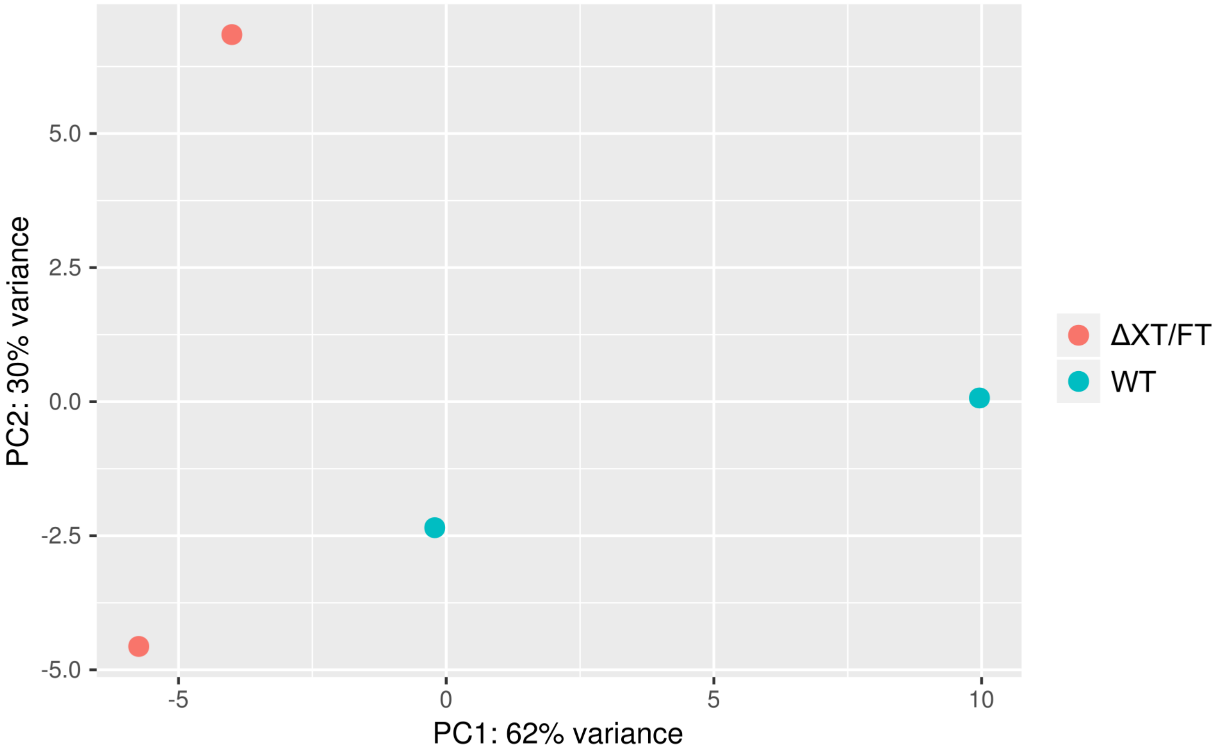

Supplementary Figure S9

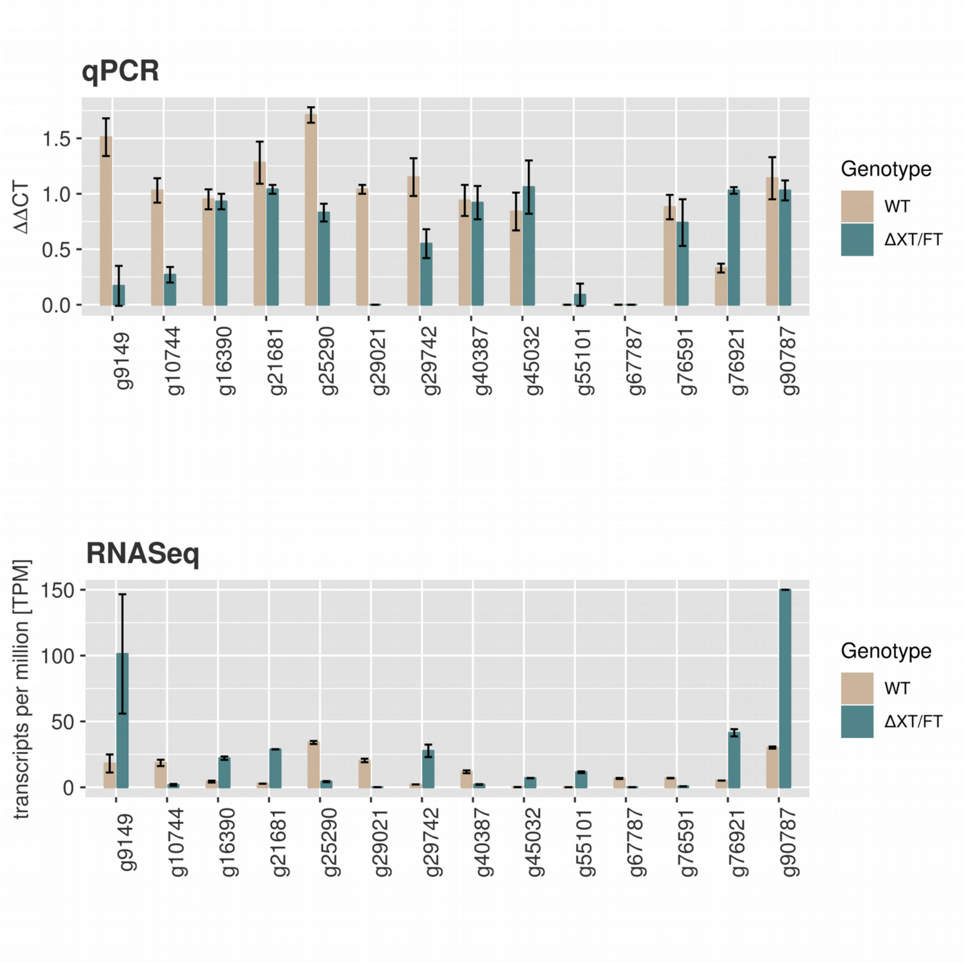

Supplementary Figure S10

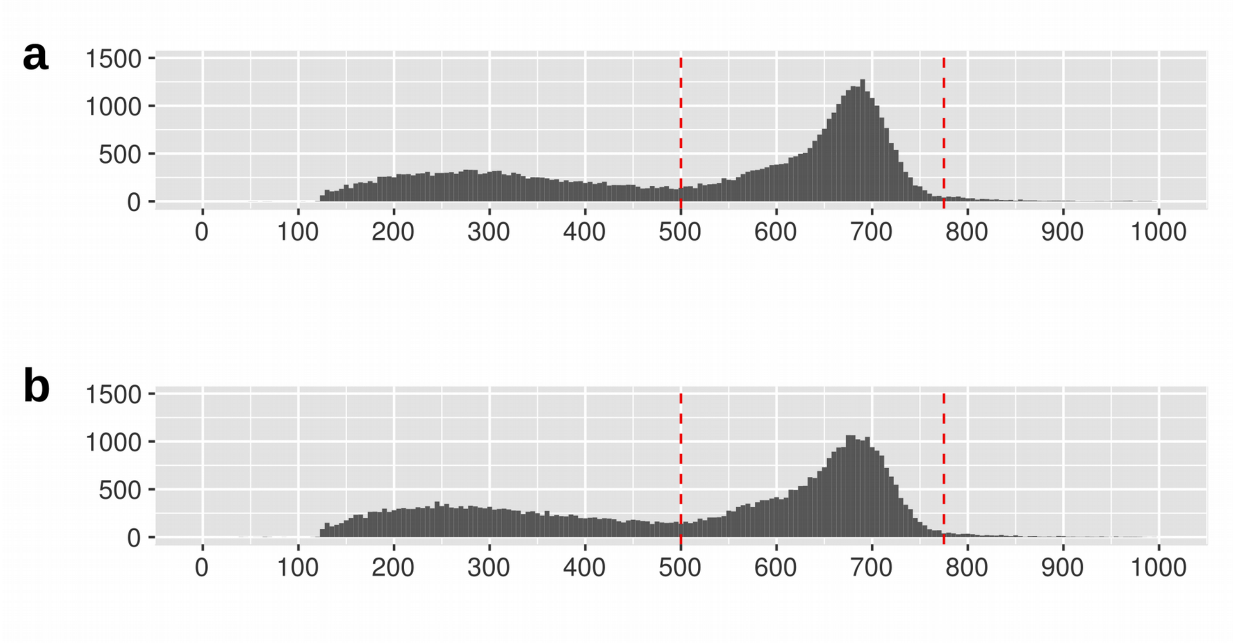

Supplementary Figure S11

Supplementary Table Legends

Supplementary Table S1. Transposable element classes within the *N. benthamiana* reference genome Nb-1 (18). Repeats were identified *de novo* using RepeatModeler; listed here are repeats masked prior to gene prediction.

Supplementary Table S2. Results of a BUSCO analysis to assess completeness of the *N. benthamiana* gene set NibSet-1 and SGN (18).

Supplementary Table S3. Number of sequences and database total length [bp] of each constructed database (downloaded from NCBI in March 2017).

Supplementary Table S4. Normalized counts as computed in DESeq2 (61) for the target genes of the FucT and XylT transgenes.The analysis was performed with wo replicates each for ΔXT/FT (light yellow) and for the wild type (dark yellow).

Supplementary Table S5. Regions of the FucT1, FucT-pseudogene, XylT1 and XylT2 transcripts (both in the NCBI and the NibSet-1 version) that are targeted by the transgenes, as obtained with Blast (see methods). The transcript version reports either the accession number of Genbank (source: "NCBI") or the gene name in NibSet-1 (source: "Augustus"). Reported are: the targeting transgene, the match length (nt), the number of matches (nt), the number of mismatches and the start-end coordinates on the transcript sequences. We report also a non-match for g40438.t1 as a means of comparison with its NCBI counterpart. Reasons are discussed within the supplementary text.

Supplementary Table S6. List of primer sequences used to perform qPCR on the differentially expressed genes found with mRNA-seq.

Supplementary Table S7. K-mer generation from the transgene sequences and their mapping onto NibSet-1. For the k-mer generation, only the sense fragments of the transgenes were used (426 nt for the FucT-transgene, 314 nt for the XylT-transgene). **a)** Total number of possible k-mers that can be generated from the transgene sequences. **b)** Number of k-mers mapped to each target (yellow) and off-target (white), for each tested k-mer size (19, 21, 25, 30). A dash represents absence of mapped k-mers.

Supplementary Figure Legends

Supplementary Figure S1. Each panel, from top to bottom: gene models obtained by mapping the NCBI FASTA sequences of the FucT and XylT genes onto the Nb-1 draft genome assembly; region targeted by the corresponding transgene; gene model predicted in NibSet-1. Gray: untranslated regions (UTR); red: coding regions (CDS); black blocks: transgene; thin lines: introns. All transcripts are shown from 5’ (left) to 3’ (right) regardless of the orientation in the genome. For each panel, a 1kb scale is shown. **a)** FucT1 (EF562630.1, g31184.t1), **b)** FucT-pseudogene (EF562631.1, g80352.t1), **c)** XylT1 (EF562628.1, g43728.t1), **d)** XylT2 (EF562629.1, g40438.t1).

Supplementary Figure S2. Genomic coverage per position in each of the two transgenes present within the ΔXT/FT genome. As both transgenes shared the same promoter and the same terminator region, in red we show the coverage in shared regions, while in cyan we show the coverage in the transgene-specific region. Horizontal dashed lines represent the average observed genomic coverage (~21x). **a)** FucT-transgene. **b)** XylT-transgene.

Supplementary Figure S3. Genomic coverage per position (red) in ΔXT/FT (**a** and **c**) and wild type (**b** and **d**) on scaffold Niben101Scf03674 (**a** and **b**) and Niben101Scf03823 (**c** and **d**). Horizontal dashed lines represents the average observed genomic coverage (~21x in ΔXT/FT, ~26x in the wild type). Vertical dashed lines show the junctions identified by chimeric reads (see results). Such junctions are visible only in the ΔXT/FT, as no transgenes are present in the wild type.

Supplementary Figure S4. Results of a re-assembly of the region where the insertion of the XylT transgene took place in scaffold Niben101Scf03823. **a)** Green and blue segments represent reassembled scaffolds in their mapping locations on the Nb-1 assembly (18). Orange segments show the transgene promoter and terminator. Numbered arrows indicate the ordering of the connections. Vertical dashed lines indicate the junctions, with their position. The horizontal dashed segment shows the uncovered region described in the results (positions 39,727 to 40,181). Grey segments indicate regions outside of the insertion junctions in scaffold Niben101Scf03823. **b)** Reordering of the insertion region, according to bridging pairs. Arrows indicate the proposed direction of each segment. The red arrow indicates both the direction of the transgene in the genome and its transcription direction. Note: the green segment is repeated twice, with reversed orientation.

Supplementary Figure S5. Alignment between the two scaffolds that contain genes g76921 and

g54961, showing aligned regions on the forward strand (red) and on the reverse strand (blue). The intersection of the highlighted areas indicates the positions where g76921 and g54961 map (scaffold Niben101Scf03674: gene g76921, positions 23406-38910; scaffold Niben101Scf00375: gene g54961 positions 151407-160415). The blue dots are approximately located on a diagonal, indicating that these two scaffolds are most likely derived from homeologous chromosomes that were inherited from the two ancestral species which gave rise to allotetraploid *N. benthamiana*.

Supplementary Figure S6. Global protein sequence alignment between genes g76921 and g54961, both annotated as TFIID subunit 12-like isoform X1. Pipes (|) indicate identical amino acids, colons (:) indicate different amino acids that share chemical properties (scoring > 0.5 in the Gonnet PAM 250 matrix), dots (.) indicate different amino acids that do not have similar chemical properties (scoring =< 0.5 in the Gonnet PAM 250 matrix). The region defining the TFIID 20 kDa protein family (PF03847) is marked in yellow in both sequences.

Supplementary Figure S7. Section of a multiple sequence alignment performed with g76921.t1, g54961.t1 and further protein sequences annotated as TFIID subunit 12-like isoform X1 available in NCBI-Protein. Black boxes indicate a region that only g76921.t1 is lacking.

Supplementary Figure S8. Folding of *N. benthamiana* proteins encoded by genes g76921 and g54961, as predicted with SWISS-MODEL.

Supplementary Figure S9. Principal component analysis (PCA) performed on the normalized read counts obtained from each replicate in each condition (total: four data points). Colors indicate the condition which each replicate (i.e. dot) belongs to.

Supplementary Figure S10. a) ΔΔCT values detected through qPCR in ΔXT/FT and WT (mean of three replicates), for each identified potential differentially expressed gene (DEG). Black lines indicate standard deviations among replicates. **b)** Transcripts per million (TPM) detected through mRNA-seq in ΔXT/FT and WT (mean of two replicates), for each identified potential DEG.

Supplementary Figure S11. Insert size estimation of the ΔXT/FT and WT genomic sequencing libraries, based on 50,000 read-pairs each mapped against the Nb-1 draft genome assembly (18). Red vertical dashed lines indicate the boundaries of insert size that were chosen for mapping (left: 500 bp; right: 775 bp). **a)** ΔXT/FT **b)** WT.

Supplementary References

70. Finn RD, Coggill P, Eberhardt RY, Eddy SR, Mistry J, Mitchell AL, et al. The Pfam protein families database: towards a more sustainable future. Nucleic Acids Research. 2016 Jan 4;44(D1):D279–85.

71. Chou PY, Fasman GD. Prediction of protein conformation. Biochemistry. 1974 Jan 15;13(2):222–45.

72. Biasini M, Bienert S, Waterhouse A, Arnold K, Studer G, Schmidt T, et al. SWISS-MODEL: modelling protein tertiary and quaternary structure using evolutionary information. Nucleic Acids Research. 2014 Jul 1;42(W1):W252–8.

73. Schwede T, Kopp J, Guex N, Peitsch MC. SWISS-MODEL: An automated protein homology-modeling server. Nucleic Acids Res. 2003 Jul 1;31(13):3381–5.

74. Deng Y, Wang CC, Choy KW, Du Q, Chen J, Wang Q, et al. Therapeutic potentials of gene silencing by RNA interference: Principles, challenges, and new strategies. Gene. 2014 Apr;538(2):217–27.

75. Martinez J, Patkaniowska A, Urlaub H, Lührmann R, Tuschl T. Single-stranded antisense siRNAs guide target RNA cleavage in RNAi. Cell. 2002 Sep 6;110(5):563–74.

76. Eulalio A, Huntzinger E, Izaurralde E. Getting to the Root of miRNA-Mediated Gene Silencing. Cell. 2008 Jan;132(1):9–14.

77. Thomas CL, Jones L, Baulcombe DC, Maule AJ. Size constraints for targeting post-transcriptional gene silencing and for RNA-directed methylation in Nicotiana benthamiana using a potato virus X vector: Size constraints for mediating PTGS and transgene methylation. The Plant Journal. 2001 Dec 23;25(4):417–25.

78. Baulcombe D. RNA silencing. Current Biology. 2002 Feb;12(3):R82–4.

79. Meister G, Tuschl T. Mechanisms of gene silencing by double-stranded RNA. Nature. 2004 Sep 16;431(7006):343–9.

80. Senthil-Kumar M, Hema R, Anand A, Kang L, Udayakumar M, Mysore KS. A systematic study to determine the extent of gene silencing in Nicotiana benthamiana and other Solanaceae species when heterologous gene sequences are used for virus-induced gene silencing. New Phytol. 2007;176(4):782–91.

81. Kurtz S, Phillippy A, Delcher AL, Smoot M, Shumway M, Antonescu C, et al. Versatile and open software for comparing large genomes. Genome Biol. 2004;5(2):R12.

82. Luo R, Liu B, Xie Y, Li Z, Huang W, Yuan J, et al. SOAPdenovo2: an empirically improved memory-efficient short-read de novo assembler. GigaScience [Internet]. 2012 Dec [cited 2018 Feb 6];1(1). Available from: https://academic.oup.com/gigascience/article-lookup/doi/10.1186/2047-

217X-1-18

83. Rice P, Longden I, Bleasby A. EMBOSS: The European Molecular Biology Open Software Suite. Trends in Genetics. 2000;16(6):276–7.

84. Edgar RC. MUSCLE: a multiple sequence alignment method with reduced time and space complexity. BMC Bioinformatics. 2004 Aug 19;5:113.

85. Eyrich VA, Przybylski D, Koh IYY, Grana O, Pazos F, Valencia A, et al. CAFASP3 in the spotlight of EVA. Proteins: Structure, Function, and Genetics. 2003;53(S6):548–60.
