# Supplementary material for: Genome and transcriptome characterization of the glycoengineered Nicotiana benthamiana line ΔXT/FT
Source: BMC Genomics. 2019 Jul 19;20:594. doi: 10.1186/s12864-019-5960-2 (PMC6642603; doi:10.1186/s12864-019-5960-2)
Supplement: Supplementary file 3 — Sequences of the constructs used for the generation of ΔXT/FT. (PDF 168 kb) [file 12864_2019_5960_MOESM3_ESM.pdf]

pGA643 vector

Reference:

- An G, Ebert P, Mitra A, Ita S (1988) Binary vectors. In SB Gelvin, RA Schilperoort, eds, Plant Molecular Biology Manual, Section A, Chapter 3. Kluwer Academic Publishers, Dordrecht, The Netherlands, pp 1-19.

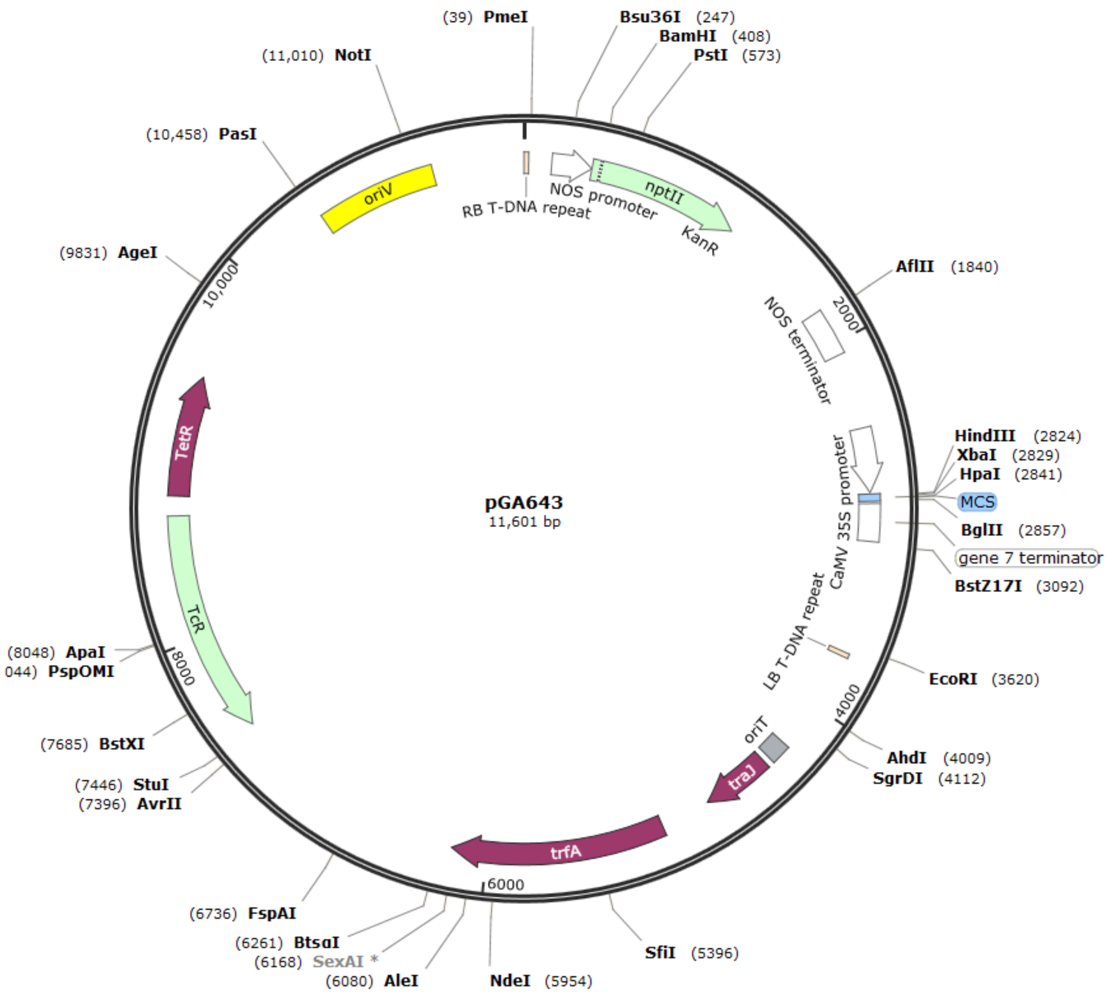

http://www.snapgene.com/resources/plasmid_files/plant_vectors/pGA643/

pGA643 sequence (empty vector)

GTTTACCCGCCAATATATCCTGTCAAACACTGATAGTTTAAACTGAAGGCGGGAAACGACAATCTGATCATGAGCGGAGA
ATTAAGGGAGTCACGTTATGACCCCCGCCGATGACGCGGGACAAGCCGTTTTACGTTTGGAACTGACAGAACCGCAACGT
TGAAGGAGCCACTCAGCCGCGGGTTTCTGGAGTTTAATGAGCTAAGCACATACGTCAGAAACCATTATTGCGCGTTCAAA
AGTCGCCTAAGGTCACTATCAGCTAGCAAATATTTCTTGTCAAAAATGCTCCACTGACGTACCATAAATTCCCCTCGGTA
TCCAATTAGAGTCTCATATTCACTCTCAATCCAAATAATCTGCAATGGCAATTACCTTATCCGCAACTTCTTTACCTATT
TCCGCCCGGATCCGGGCAGGTTCTCCGGCCGCTTGGGTGGAGAGGCTATTCGGCTATGACTGGGCACAACAGACAATCGG
CTGCTCTGATGCCGCCGTGTTCCGGCTGTCAGCGCAGGGGCGCCCGGTTCTTTTTGTCAAGACCGACCTGTCCGGTGCCC
TGAATGAACTGCAGGACGAGGCAGCGCGGCTATCGTGGCTGGCCACGACGGGCGTTCCTTGCGCAGCTGTGCTCGACGTT
GTCACTGAAGCGGGAAGGGACTGGCTGCTATTGGGCGAAGTGCCGGGGCAGGATCTCCTGTCATCTCACCTTGCTCCTGC
CGAGAAAGTATCCATCATGGCTGATGCAATGCGGCGGCTGCATACGCTTGATCCGGCTACCTGCCCATTCGACCACCAAG
CGAAACATCGCATCGAGCGAGCACGTACTCGGATGGAAGCCGGTCTTGTCGATCAGGATGATCTGGACGAAGAGCATCAG

GGGCTCGCGCCAGCCGAACTGTTCGCCAGGCTCAAGGCGCGCATGCCCGACGGCGAGGATCTCGTCGTGACCCATGGCGA
TGCCTGCTTGCCGAATATCATGGTGGAAAATGGCCGCTTTTCTGGATTCATCGACTGTGGCCGGCTGGGTGTGGCGGACC
GCTATCAGGACATAGCGTTGGCTACCCGTGATATTGCTGAAGAGCTTGGCGGCGAATGGGCTGACCGCTTCCTCGTGCTT
TACGGTATCGCCGCTCCCGATTCGCAGCGCATCGCCTTCTATCGCCTTCTTGACGAGTTCTTCTGAGCGGGACTCTGGGG
TTCGAAATGACCGACCAAGCGACGCCCAACCTGCCATCACGAGATTTCGATTCCACCGCCGCCTTCTATGAAAGGTTGGG
CTTCGGAATCGTTTTCCGGGACGCCGGCTGGATGATCCTCCAGCGCGGGGATCTCATGCTGGAGTTCTTCGCCCACCCCG
ATCCAACACTTACGTTTGCAACGTCCAAGAGCAAATAGACCACGAACGCCGGAAGGTTGCCGCAGCGTGTGGATTGCGTC
TCAATTCTCTCTTGCAGGAATGCAATGATGAATATGATACTGACTATGAAACTTTGAGGGAATACTGCCTAGCACCGTCA
CCTCATAACGTGCATCATGCATGCCCTGACAACATGGAACATCGCTATTTTTCTGAAGAATTATGCTCGTTGGAGGATGT
CGCGGCAATTGCAGCTATTGCCAAAATCGAAATACCCCTCACGCATGCATTCATCAATATTATTCATGCGGGGAAAGGCA
AGATTAATCCAACTGGCAAATCATCCAGCGTGATTGGTAACTTCAGTTCCAGCGACTTGATTCGTTTTGGTGCTACCCAC
GTTTTCAATAAGGACGAGATGGTGGAGTAAAGAAGGAGTGCGTCGAAGCAGATCGTTCAAACATTTGGCAATAAAGTTTC
TTAAGATTGAATCCTGTTGCCGGTCTTGCGATGATTATCATATAATTTCTGTTGAATTACGTTAAGCATGTAATAATTAA
CATGTAATGCATGACGTTATTTATGAGATGGGTTTTTATGATTAGAGTCCCGCAATTATACATTTAATACGCGATAGAAA
ACAAAATATAGCGCGCAAACTAGGATAAATTATCGCGCGCGGTGTCATCTATGTTACTAGATCGATCAAACTTCGGTACT
GTGTAATGACGATGAGCAATCGAGAGGCTGACTAACAAAAGGTATGCCCAAAAACAACCTCTCCAAACTGTTTCGAATTG
GAAGTTTCTGCTCATGCCGACAGGCATAACTTAGATATTCGCGGGCTATTCCCACTAATTCGTCCTGCTGGTTTGCGCCA
AGATAAATCAGTGCATCTCCTTACAAGTTCCTCTGTCTTGTGAAATGAACTGCTGACTGCCCCCCAAGAAAGCCTCCTCA
TCTCCCAGTTGGCGGCGGCTGATACACCATCGAAAACCCACGTCCGAACACTTGATACATGTGCCTGAGAAATAGGAACA
TGGTGGAGCACGACACTCTCGTCTACTCCAAGAATATCAAAGATACAGTCTCAGAAGACCAAAGGGCTATTGAGACTTTT
CAACAAAGGGTAATATCGGGAAACCTCCTCGGATTCCATTGCCCAGCTATCTGTCACTTCATCAAAAGGACAGTAGAAAA
GGAAGGTGGCACCTACAAATGCCATCATTGCGATAAAGGAAAGGCTATCGTTCAAGATGCCTCTGCCGACAGTGGTCCCA
AAGATGGACCCCCACCCACGAGGAGCATCGTGGAAAAAGAAGACGTTCCAACCACGTCTTCAAAGCAAGTGGATTGATGT
GATATCTCCACTGACGTAAGGGATGACGCACAATCCCACTATCCTTCGCAAGACCCTTCCTCTATATAAGGAAGTTCATT
TCATTTGGAGAGGACCCTCGACCAAGCTTCTAGAGCTCGTTAACGGTACCATCGATAGATCTGCGATGAGCTAAGCTAGC
TATATCATCAATTTATGTATTACACATAATATCGCACTCAGTCTTTCATCTACGGCAATGTACCAGCTGATATAATCAGT
TATTGAAATATTTCTGAATTTAAACTTGCATCAATAAATTTATGTTTTTGCTTGGACTATAATACCTGACTTGTTATTTT
ATCAATAAATATTTAAACTATATTTCTTTCAAGATATCATTCTTTACAAGTATACGTGTTTAAATTGAATACCATAAATT
TTTATTTTTCAAATACATGTAAAATTATGAAATGGGAGTGGTGGCGACCGAGCTCAAGCACACTTCAATTCCTATAACGG
ACCAAATCGCAAAAATTATAATAACATATTATTTCATCCTGGATTAAAAGAAAGTCACCGGGGATTATTTTGTGACGCCG
ATTACATACGGCGACAATAAAGACATTGGAAATCGTAGTACATATTGGAATACACTGATTATATTAATGATGAATACATA
CTTTAATATCCTTACGTAGGATCAACATATCTTGTTACAATCGGACACTTTTGCTTCATCCCCGCTAACACCTCTGCACC
TTAGACCAAGCGCTTCCACAAGGAACTGAGAGCCATAGCCCACCTCACCTTGGGTTCCTTTGGCCGCCTGTCTTTCTGAA
AGAGAGCCTTGCCCACCGCAACTATTTCAACACAGATAGGATCAACCCGGGATGGCGCTAAGAAGCTATTGCCGCCGATC
TTCATAGGGTACCGAGCTCGAATTCAGTACATTAAAAACGTCCGCAATGTGTTATTAAGTTGTCTAAGCGTCAATTTGTT
TACACCACAATATATCCTGCCACCAGCCAGCCAACAGCTCCCCGACCGGCAGCTCGGCACAAAATCACCACTCGATACAG
GCAGCCCATCAGTCCGGGACGGCGTCAGCGGGAGAGCCGTTGTAAGGCGGCAGACTTTGCTCATGTTACCGATGCTATTC
GGAAGAACGGCAACTAAGCTGCCGGGTTTGAAACACGGATGATCTCGCGGAGGGTAGCATGTTGATTGTAACGATGACAG
AGCGTTGCTGCCTGTGATCAAATATCATCTCCCTCGCAGAGATCCGAATTATCAGCCTTCTTATTCATTTCTCGCTTAAC
CGTGACAGGCTGTCGATCTTGAGAACTATGCCGACATAATAGGAAATCGCTGGATAAAGCCGCTGAGGAAGCTGAGTGGC
GCTATTTCTTTAGAAGTGAACGTTGACGATCGTCGACGGATCTTTTCCGCTGCATAACCCTGCTTCGGGGTCATTATAGC
GATTTTTTCGGTATATCCATCCTTTTTCGCACGATATACAGGATTTTGCCAAAGGGTTCGTGTAGACTTTCCTTGGTGTA
TCCAACGGCGTCAGCCGGGCAGGATAGGTGAAGTAGGCCCACCCGCGAGCGGGTGTTCCTTCTTCACTGTCCCTTATTCG
CACCTGGCGGTGCTCAACGGGAATCCTGCTCTGCGAGGCTGGCCGGCTACCGCCGGCGTAACAGATGAGGGCAAGCGGAT
GGCTGATGAAACCAAGCCAACCAGGAAGGGCAGCCCACCTATCAAGGTGTACTGCCTTCCAGACGAACGAAGAGCGATTG
AGGAAAAGGCGGCGGCGGCCGGCATGAGCCTGTCGGCCTACCTGCTGGCCGTCGGCCAGGGCTACAAAATCACGGGCGTC
GTGGACTATGAGCACGTCCGCGAGCTGGCCCGCATCAATGGCGACCTGGGCCGCCTGGGCGGCCTGCTGAAACTCTGGCT
CACCGACGACCCGCGCACGGCGCGGTTCGGTGATGCCACGATCCTCGCCCTGCTGGCGAAGATCGAAGAGAAGCAGGACG
AGCTTGGCAAGGTCATGATGGGCGTGGTCCGCCCGAGGGCAGAGCCATGACTTTTTTAGCCGCTAAAACGGCCGGGGGGT
GCGCGTGATTGCCAAGCACGTCCCCATGCGCTCCATCAAGAAGAGCGACTTCGCGGAGCTGGTATTCGTGCAGGGCAAGA
TTCGGAATACCAAGTACGAGAAGGACGGCCAGACGGTCTACGGGACCGACTTCATTGCCGATAAGGTGGATTATCTGGAC
ACCAAGGCACCAGGCGGGTCAAATCAGGAATAAGGGCACATTGCCCCGGCGTGAGTCGGGGCAATCCCGCAAGGAGGGTG
AATGAATCGGACGTTTGACCGGAAGGCATACAGGCAAGAACTGATCGACGCGGGGTTTTCCGCCGAGGATGCCGAAACCA
TCGCAAGCCGCACCGTCATGCGTGCGCCCCGCGAAACCTTCCAGTCCGTCGGCTCGATGGTCCAGCAAGCTACGGCCAAG
ATCGAGCGCGACAGCGTGCAACTGGCTCCCCCTGCCCTGCCCGCGCCATCGGCCGCCGTGGAGCGTTCGCGTCGTCTCGA
ACAGGAGGCGGCAGGTTTGGCGAAGTCGATGACCATCGACACGCGAGGAACTATGACGACCAAGAAGCGAAAAACCGCCG
GCGAGGACCTGGCAAAACAGGTCAGCGAGGCCAAGCAGGCCGCGTTGCTGAAACACACGAAGCAGCAGATCAAGGAAATG
CAGCTTTCCTTGTTCGATATTGCGCCGTGGCCGGACACGATGCGAGCGATGCCAAACGACACGGCCCGCTCTGCCCTGTT
CACCACGCGCAACAAGAAAATCCCGCGCGAGGCGCTGCAAAACAAGGTCATTTTCCACGTCAACAAGGACGTGAAGATCA
CCTACACCGGCGTCGAGCTGCGGGCCGACGATGACGAACTGGTGTGGCAGCAGGTGTTGGAGTACGCGAAGCGCACCCCT
ATCGGCGAGCCGATCACCTTCACGTTCTACGAGCTTTGCCAGGACCTGGGCTGGTCGATCAATGGCCGGTATTACACGAA
GGCCGAGGAATGCCTGTCGCGCCTACAGGCGACGGCGATGGGCTTCACGTCCGACCGCGTTGGGCACCTGGAATCGGTGT
CGCTGCTGCACCGCTTCCGCGTCCTGGACCGTGGCAAGAAAACGTCCCGTTGCCAGGTCCTGATCGACGAGGAAATCGTC
GTGCTGTTTGCTGGCGACCACTACACGAAATTCATATGGGAGAAGTACCGCAAGCTGTCGCCGACGGCCCGACGGATGTT
CGACTATTTCAGCTCGCACCGGGAGCCGTACCCGCTCAAGCTGGAAACCTTCCGCCTCATGTGCGGATCGGATTCCACCC
GCGTGAAGAAGTGGCGCGAGCAGGTCGGCGAAGCCTGCGAAGAGTTGCGAGGCAGCGGCCTGGTGGAACACGCCTGGGTC
AATGATGACCTGGTGCATTGCAAACGCTAGGGCCTTGTGGGGTCAGTTCCGGCTGGGGGTTCAGCAGCCAGCGCTTTACT
GGCATTTCAGGAACAAGCGGGCACTGCTCGACGCACTTGCTTCGCTCAGTATCGCTCGGGACGCACGGCGCGCTCTACGA
ACTGCCGATAAACAGAGGATTAAAATTGACAATTGTGATTAAGGCTCAGATTCGACGGCTTGGAGCGGCCGACGTGCAGG
ATTTCCGCGAGATCCGATTGTCGGCCCTGAAGAAAGCTCCAGAGATGTTCGGGTCCGTTTACGAGCACGAGGAGAAAAAG
CCCATGGAGGCGTTCGCTGAACGGTTGCGAGATGCCGTGGCATTCGGCGCCTACATCGACGGCGAGATCATTGGGCTGTC
GGTCTTCAAACAGGAGGACGGCCCCAAGGACGCTCACAAGGCGCATCTGTCCGGCGTTTTCGTGGAGCCCGAACAGCGAG
GCCGAGGGGTCGCCGGTATGCTGCTGCGGGCGTTGCCGGCGGGTTTATTGCTCGTGATGATCGTCCGACAGATTCCAACG
GGAATCTGGTGGATGCGCATCTTCATCCTCGGCGCACTTAATATTTCGCTATTCTGGAGCTTGTTGTTTATTTCGGTCTA
CCGCCTGCCGGGCGGGGTCGCGGCGACGGTAGGCGCTGTGCAGCCGCTGATGGTCGTGTTCATCTCTGCCGCTCTGCTAG
GTAGCCCGATACGATTGATGGCGGTCCTGGGGGCTATTTGCGGAACTGCGGGCGTGGCGCTGTTGGTGTTGACACCAAAC
GCAGCGCTAGATCCTGTCGGCGTCGCAGCGGGCCTGGCGGGGGCGGTTTCCATGGCGTTCGGAACCGTGCTGACCCGCAA
GTGGCAACCTCCCGTGCCTCTGCTCACCTTTACCGCCTGGCAACTGGCGGCCGGAGGACTTCTGCTCGTTCCAGTAGCTT
TAGTGTTTGATCCGCCAATCCCGATGCCTACAGGAACCAATGTTCTCGGCCTGGCGTGGCTCGGCCTGATCGGAGCGGGT
TTAACCTACTTCCTTTGGTTCCGGGGGATCTCGCGACTCGAACCTACAGTTGTTTCCTTACTGGGCTTTCTCAGCCCGGG

GACCGCCGTGTTGCTAGGATGGTTGTTCTTGGATCAGACGCTGAGTGCGCTTCAAATCATCGGCGTCCTGCTCGTGATCG
GGAGTATCTGGCTGGGCCAACGTTCCAACCGCACTCCTAGGGCGCGTATAGCTTGCCGGAAGTCGCCTTGACCCGCATGG
CATAGGCCTATCGTTTCCACGATCAGCGATCGGCTCGTTGCCCTGCGCCGCTCCAAAGCCCGCGACGCAGCGCCGGCAGG
CAGAGCAAGTAGAGGGCAGCGCCTGCAATCCATGCCCACCCGTTCCACGTTGTTATAGAAGCCGCATAGATCGCCGTGAA
GAGGAGGGGTCCGACGATCGAGGTCAGGCTGGTGAGCGCCGCCAGTGAGCCTTGCAGCTGCCCCTGACGTTCCTCATCCA
CCTGCCTGGACAACATTGCTTGCAGCGCCGGCATTCCGATGCCACCCGAAGCAAGCAGGACCATGATCGGGAACGCCATC
CATCCCCGTGTCGCGAAGGCAAGCAGGATGTAGCCTGTGCCGTCGGCAATCATTCCGAGCATGAGTGCCCGCCTTTCGCC
GAGCCGGGCGGCTACAGGGCCGGTGATCATTGCCTGGGCGAGTGAATGCAGAATGCCAAATGCGGCAAGCGAAATGCCGA
TCGTGGTCGCGTCCCAGTGAAAGCGATCCTCGCCGAAAATGACCCAAAGCGCGGCCGGCACCTGTCCGACAAGTTGCATG
ATGAAGAAGACCGCCATCAGGGCGGCGACGACGGTCATGCCCCGGGCCCACCGGAACGAAGCGAGCGGGTTGAGAGCCTC
CCGGCGTAACGGCCGGCGTTCGCCTTTGTGCGACTCCGGCAAAAGGAAACAGCCCGTCAGGAAATTGAGGCCGTTCAAGG
CTGCCGCGGCGAAGAACGGAGCGTGGGGGGAGAAACCGCCCATCAGCCCACCGAGCACAGGTCCCGCGACCATCCCGAAC
CCGAAACAGGCGCTCATGAAGCCGAAGTGCCGCGCGCGCTCATCGCCATCAGTGATATCGGCAATATAAGCGCCGGCTAC
CGCCCCAGTCGCCCCGGTGATGCCGGCCACGATCCGCCCGATATAGAGAACCCAAAGGAAAGGCGCTGTCGCCATGATGG
CGTAGTCGACAGTGGCGCCGGCCAGCGAGACGAGCAAGATTGGCCGCCGCCCGAAACGATCCGACAGCGCGCCCAGCACA
GGTGCGCAGGCAAATTGCACCAACGCATACAGCGCCAGCAGAATGCCATAGTGGGCGGTGACGTCGTTCGAGTGAACCAG
ATCGCGCAGGAGGCCCGGCAGCACCGGCATAATCAGGCCGATGCCGACAGCGTCGAGCGCGACAGTGCTCAGAATTACGA
TCAGGGGTATGTTGGGTTTCATGTCTGGCCTCCGGACCAGCCTCCGCTGGTCCGATTGAACGCGCGGATTCTTTATCACT
GATAAGTTGGTGGACATATTATGTTTATCAGTGATAAAGTGTCAAGCATGACAAAGTTGCAGCCGAATACAGTGATCCGT
GCCGCCCTGGACCTGTTGAACGAGGTCGGCGTAGACGGTCTGACGACACGCAAACTGGCGGAACGGTTGGGGGTTCAGCA
GCCGGCGCTTTACTGGCACTTCAGGAACAAGCGGGCGCTGCTCGACGCACTGGCCGAAGCCATGCTGGCGGAGAATCATA
CGCATTCGGTGCCGAGAGCCGACGACGACTGGCGCTCATTTCTGATCGGGAATGCCCGCAGCTTCAGGCAGGCGCTGCTC
GCCTACCGCGATGGCGCGCGCATCCATGCCGGCACGCGACCGGGCGCACCGCAGATGGAAACGGCCGACGCGCAGCTTCG
CTTCCTCTGCGAGGCGGGTTTTTCGGCCGGGGACGCCGTCAATGCGCTGATGACAATCAGCTACTTCACTGTTGGGGCCG
TGCTTGAGGAGCAGGCCGGCGACAGCGATGCCGGCGAGCGCGGCGGCACCGTTGAACAGGCTCCGCTCTCGCCGCTGTTG
CGGGCCGCGATAGACGCCTTCGACGAAGCCGGTCCGGACGCAGCGTTCGAGCAGGGACTCGCGGTGATTGTCGATGGATT
GGCGAAAAGGAGGCTCGTTGTCAGGAACGTTGAAGGACCGAGAAAGGGTGACGATTGATCAGGACCGCTGCCGGAGCGCA
ACCCACTCACTACAGCAGAGCCATGTAGACAACATCCCCTCCCCCTTTCCACCGCGTCAGACGCCCGTAGCAGCCCGCTA
CGGGCTTTTTCATGCCCTGCCCTAGCGTCCAAGCCTCACGGCCGCGCTCGGCCTCTCTGGCGGCCTTCTGGCGCTCCTGC
TGCGGCGTCCGCTCGTGGGCCGTGGCGCGGTCCGCGCGCCGGCCTCGTGCGCCTGGCGCTCGCGGGCGAGGTCCAGGGC
GGCCGTCTTCACGTTCTGCCTTGCGCAGATGAGATAGATCCAGTCTGCAAGTGTCAGAAACGAGTCTGCATTTGATGCCC
TGAACGTCTTCATACATCTGATAAGCCCCTCCCGGAAGTCTGCTTTCTCTCCGTCAGAAAGCATACCCTGACCGGTTTCA
ATCACGGACCGTTCTGTCAGGTAATGCTCTGTCCAGTCATACAGAGCCGGGTTTGTCACCCGTATAAGCTGAAGCAGGCA
CAAATCAGGGAAATAAACAAAATCCCGCATCCCCGGATAAAGAAAAATCAGGGAATTAATGGCCTGATGGATTTCCCGTG
GCGTCGAAAGTGCGGCACCATAGGTATCAGTCACCGCGATGAGATCCCTTACCATTCCAGAGTCTGGCGGTTGATTATTA
ATTTGCTGATATAGAGCCTCAGCCCGCTGGCGAAATTCATTACGTAAATCAAAGGCTTCAGGTCGGGGTAATTTAAAACT
AAGCTGAATGATTTTCTGGAGATAACGGCTGCCATCTTCGATATTCAGCGCATGTTCAACGGCATGAGTGATAATCTGCC
TGTCATAACAGAGAATATGGGTAAAGCGGGGCAGATCGGCTACTGCACGCACAAGCCTGAACACTTCCGCCACCTGGGAT
GGCTCCAGTCGGTCCAGATCATCCATGACAACAATGAACTTCAGATCCAGACTCACCAGTTGTCCTGCAATTTCAGCCCG
AAGCTAGCTTGGCTGCCATTTTTGGGGTGAGGCCGTTCGCGGCCGAGGGGCGCAGCCCCTGGGGGGATGGGAGGCCCGCG
TTAGCGGGCCGGGAGGGTTCGAGAAGGGGGGGCACCCCCCTTCGGCGTGCGCGGTCACGCGCACAGGGCGCAGCCCTGGT
TAAAAACAAGGTTTATAAATATTGGTTTAAAAGCAGGTTAAAAGACAGGTTAGCGGTGGCCGAAAAACGGGCGGAAACCC
TTGCAAATGCTGGATTTTCTGCCTGTGGACAGCCCCTCAAATGTCAATAGGTGCGCCCCTCATCTGTCAGCACTCTGCCC
CTCAAGTGTCAAGGATCGCGCCCCTCATCTGTCAGTAGTCGCGCCCCTCAAGTGTCAATACCGCAGGGCACTTATCCCCA
GGCTTGTCCACATCATCTGTGGGAAACTCGCGTAAAATCAGGCGTTTTCGCCGATTTGCGAGGCTGGCCAGCTCCACGTC
GCCGGCCGAAATCGAGCCTGCCCCTCATCTGTCAACGCCGCGCCGGGTGAGTCGGCCCCTCAAGTGTCAACGTCCGCCCC
TCATCTGTCAGTGAGGGCCAAGTTTTCCGCGAGGTATCCACAACGCCGGCGGCCGCGGTGTCTCGCACACGGCTTCGACG
GCGTTTCTGGCGCGTTTGCAGGGCCATAGACGGCCGCCAGCCCAGCGGCGAGGGCAACCAGCCCGGTGAGCGTCGGAAAG
GGTCGACGATCTTGCTGCGTTCGGATATTTTCGTGGAGTTCCCGCCACAGACCCGGATTGAAGGCGAGATCCAGCAACTC
GCGCCAGATCATCCTGTGACGGAACTTTGGCGCGTGATGACTGGCCAGGACGTCGGCCGAAAGAGCGACAAGCAGATCAC
GCTTTTCGACAGCGTCGGATTTGCGATCGAGGATTTTTCGGCGCTGCGCTACGTCCGCGACCGCGTTGAGGGATCAAGCC
ACAGCAGCCCACTCGACCTTCTAGCCGACCCAGACGAGCCAAGGGATCTTTTTGGAATGCTGCTCCGTCGTCAGGCTTTC
CGACGTTTGGGTGGTTGAACAGAAGTCATTATCGCACGGAATGCCAAGCACTCCCGAGGGGAACCCTGTGGTTGGCATGC
ACATACAAATGGACGAACGGATAAACCTTTTCACGCCCTTTTAAATATCCGATTATTCTAATAAACGCTCTTTTCTCTTA
G

GTTTACCCGCCAATATATCCTGTCA: RB-DNA repeat

GTTTACACCACAATATATCCTGCCA: LB-DNA repeat

pGAX1 (*N. benthamiana* XylT RNAi vector)
The assembled sequence was cloned into XbaI linearized plant expression vector pGA643 to create pGAX1.

XylT sense-intron-antisense RNAi fragment:

```
TCTAGATTAGCAATGAAGAGCAAGTATTTGATTCCATAAAGAGCTGGGCCTTAAACCACTCGGAGTGCAAATTAAATGTA
ATTAGTGGATTGTTTGCCCACATGTCCATGAAAGAGCAAGTTCGAGCAATCCAAGATGCTTTTGTCATTGTTGGTGCTCA
TGGAGCAGGTCTAACCCACATAGTTTCTGCAGCACCAAAAGCTGTAATACTAGAAATTATAAGCAGCGAATATAGGCGCC
CCCATTTTGCTCTGATTGCTCAATGGAAAGGATTGGAGTACCATCCCATATATTTGGAGGGGTCTTATGCGGATCCACTG
cacggtatgctcctcttcttgttcatggtcatgatccttatatgagcagggaaagtccagtttagacttgtagttagtta
ctcttcgttataggatttggatttcttgcgtgtttatggttttagtttccctcctttgatgaataaaattgaatcttgta
tgagtttcatatccatgttgtgaatctttttgcagacgcagctAGGTACCGCATAAGACCCCTCCAAATATATGGGATGG
TACTCCAATCCTTTCCATTGAGCAATCAGAGCAAAATGGGGGCGCCTATATTCGCTGCTTATAATTTCTAGTATTACAGC
TTTTGGTGCTGCAGAAACTATGTGGGTTAGACCTGCTCCATGAGCACCAACAATGACAAAAGCATCTTGGATTGCTCGAA
CTTGCTCTTTCATGGACATGTGGGCAAACAATCCACTAATTACATTTAATTTGCACTCCGAGTGGTTTAAGGCCCAGCTC
TTTATGGAATCAAATACTTGCTCTTCATTGCTAATCTAGA
```

yellow: XbaI site

GTTTACCCGCCAATATATCCTGTCA: RB-DNA repeat

GTTTACACCACAATATATCCTGCCA: LB-DNA repeat

pGAX1 sequence

```
GTTTACCCGCCAATATATCCTGTCAAACACTGATAGTTTAAACTGAAGGCGGGAAACGACAATCTGATCATGAGCGGAGA
ATTAAGGGAGTCACGTTATGACCCCCGCCGATGACGCGGGACAAGCCGTTTTACGTTTGGAACTGACAGAACCGCAACGT
TGAAGGAGCCACTCAGCCGCGGGTTTCTGGAGTTTAATGAGCTAAGCACATACGTCAGAAACCATTATTGCGCGTTCAAA
AGTCGCCTAAGGTCACTATCAGCTAGCAAATATTTCTTGTCAAAAATGCTCCACTGACGTACCATAAATTCCCCTCGGTA
TCCAATTAGAGTCTCATATTCACTCTCAATCCAAATAATCTGCAATGGCAATTACCTTATCCGCAACTTCTTTACCTATT
TCCGCCCGGATCCGGGCAGGTTCTCCGGCCGCTTGGGTGGAGAGGCTATTCGGCTATGACTGGGCACAACAGACAATCGG
CTGCTCTGATGCCGCCGTGTTCCGGCTGTCAGCGCAGGGGCGCCCGGTTCTTTTTGTCAAGACCGACCTGTCCGGTGCCC
TGAATGAACTGCAGGACGAGGCAGCGCGGCTATCGTGGCTGGCCACGACGGGCGTTCCTTGCGCAGCTGTGCTCGACGTT
GTCACTGAAGCGGGAAGGGACTGGCTGCTATTGGGCGAAGTGCCGGGGCAGGATCTCCTGTCATCTCACCTTGCTCCTGC
CGAGAAAGTATCCATCATGGCTGATGCAATGCGGCGGCTGCATACGCTTGATCCGGCTACCTGCCCATTCGACCACCAAG
CGAAACATCGCATCGAGCGAGCACGTACTCGGATGGAAGCCGGTCTTGTCGATCAGGATGATCTGGACGAAGAGCATCAG
GGGCTCGCGCCAGCCGAACTGTTCGCCAGGCTCAAGGCGCGCATGCCCGACGGCGAGGATCTCGTCGTGACCCATGGCGA
TGCCTGCTTGCCGAATATCATGGTGGAAAATGGCCGCTTTTCTGGATTCATCGACTGTGGCCGGCTGGGTGTGGCGGACC
GCTATCAGGACATAGCGTTGGCTACCCGTGATATTGCTGAAGAGCTTGGCGGCGAATGGGCTGACCGCTTCCTCGTGCTT
TACGGTATCGCCGCTCCCGATTCGCAGCGCATCGCCTTCTATCGCCTTCTTGACGAGTTCTTCTGAGCGGGACTCTGGGG
TTCGAAATGACCGACCAAGCGACGCCCAACCTGCCATCACGAGATTTCGATTCCACCGCCGCCTTCTATGAAAGGTTGGG
CTTCGGAATCGTTTTCCGGGACGCCGGCTGGATGATCCTCCAGCGCGGGGATCTCATGCTGGAGTTCTTCGCCCACCCCG
ATCCAACACTTACGTTTGCAACGTCCAAGAGCAAATAGACCACGAACGCCGGAAGGTTGCCGCAGCGTGTGGATTGCGTC
TCAATTCTCTCTTGCAGGAATGCAATGATGAATATGATACTGACTATGAAACTTTGAGGGAATACTGCCTAGCACCGTCA
CCTCATAACGTGCATCATGCATGCCCTGACAACATGGAACATCGCTATTTTTCTGAAGAATTATGCTCGTTGGAGGATGT
CGCGGCAATTGCAGCTATTGCCAAAATCGAAATACCCCTCACGCATGCATTCATCAATATTATTCATGCGGGGAAAGGCA
AGATTAATCCAACTGGCAAATCATCCAGCGTGATTGGTAACTTCAGTTCCAGCGACTTGATTCGTTTTGGTGCTACCCAC
GTTTTCAATAAGGACGAGATGGTGGAGTAAAGAAGGAGTGCGTCGAAGCAGATCGTTCAAACATTTGGCAATAAAGTTTC
TTAAGATTGAATCCTGTTGCCGGTCTTGCGATGATTATCATATAATTTCTGTTGAATTACGTTAAGCATGTAATAATTAA
CATGTAATGCATGACGTTATTTATGAGATGGGTTTTTATGATTAGAGTCCCGCAATTATACATTTAATACGCGATAGAAA
ACAAAATATAGCGCGCAAACTAGGATAAATTATCGCGCGCGGTGTCATCTATGTTACTAGATCGATCAAACTTCGGTACT
GTGTAATGACGATGAGCAATCGAGAGGCTGACTAACAAAAGGTATGCCCAAAAACAACCTCTCCAAACTGTTTCGAATTG
GAAGTTTCTGCTCATGCCGACAGGCATAACTTAGATATTCGCGGGCTATTCCCACTAATTCGTCCTGCTGGTTTGCGCCA
AGATAAATCAGTGCATCTCCTTACAAGTTCCTCTGTCTTGTGAAATGAACTGCTGACTGCCCCCCAAGAAAGCCTCCTCA
TCTCCCAGTTGGCGGCGGCTGATACACCATCGAAAACCCACGTCCGAACACTTGATACATGTGCCTGAGAAATAGGAACA
TGGTGGAGCACGACACTCTCGTCTACTCCAAGAATATCAAAGATACAGTCTCAGAAGACCAAAGGGCTATTGAGACTTTT
CAACAAAGGGTAATATCGGGAAACCTCCTCGGATTCCATTGCCCAGCTATCTGTCACTTCATCAAAAGGACAGTAGAAAA
GGAAGGTGGCACCTACAAATGCCATCATTGCGATAAAGGAAAGGCTATCGTTCAAGATGCCTCTGCCGACAGTGGTCCCA
AAGATGGACCCCCACCCACGAGGAGCATCGTGGAAAAAGAAGACGTTCCAACCACGTCTTCAAAGCAAGTGGATTGATGT
GATATCTCCACTGACGTAAGGGATGACGCACAATCCCACTATCCTTCGCAAGACCCTTCCTCTATATAAGGAAGTTCATT
TCATTTGGAGAGGACCCTCGACCAAGCTTCTAGATTAGCAATGAAGAGCAAGTATTTGATTCCATAAAGAGCTGGGCCTT
AAACCACTCGGAGTGCAAATTAAATGTAATTAGTGGATTGTTTGCCCACATGTCCATGAAAGAGCAAGTTCGAGCAATCC
AAGATGCTTTTGTCATTGTTGGTGCTCATGGAGCAGGTCTAACCCACATAGTTTCTGCAGCACCAAAAGCTGTAATACTA
GAAATTATAAGCAGCGAATATAGGCGCCCCCATTTTGCTCTGATTGCTCAATGGAAAGGATTGGAGTACCATCCCATATA
```

TTTGGAGGGGTCTTATGCGGATCCACTGcacggtatgctcctcttcttgttcatggtcatgatccttatatgagcaggga
aagtccagtttagacttgtagttagttactcttcgttataggatttggatttcttgcgtgtttatggttttagtttccct
cctttgatgaataaaattgaatcttgtatgagtttcatatccatgttgtgaatctttttgcagacgcagctAGGTACCGC
ATAAGACCCCTCCAAATATATGGGATGGTACTCCAATCCTTTCCATTGAGCAATCAGAGCAAAATGGGGGCGCCTATATT
CGCTGCTTATAATTTCTAGTATTACAGCTTTTGGTGCTGCAGAAACTATGTGGGTTAGACCTGCTCCATGAGCACCAACA
ATGACAAAAGCATCTTGGATTGCTCGAACTTGCTCTTTCATGGACATGTGGGCAAACAATCCACTAATTACATTTAATTT
GCACTCCGAGTGGTTTAAGGCCCAGCTCTTTATGGAATCAAATACTTGCTCTTCATTGCTAATCTAGAGCTCGTTAACGG
TACCATCGATAGATCTGCGATGAGCTAAGCTAGCTATATCATCAATTTATGTATTACACATAATATCGCACTCAGTCTTT
CATCTACGGCAATGTACCAGCTGATATAATCAGTTATTGAAATATTTCTGAATTTAAACTTGCATCAATAAATTTATGTT
TTTGCTTGGACTATAATACCTGACTTGTTATTTTATCAATAAATATTTAAACTATATTTCTTTCAAGATATCATTCTTTA
CAAGTATACGTGTTTAAATTGAATACCATAAATTTTTATTTTTCAAATACATGTAAAATTATGAAATGGGAGTGGTGGCG
ACCGAGCTCAAGCACACTTCAATTCCTATAACGGACCAAATCGCAAAAATTATAATAACATATTATTTCATCCTGGATTA
AAAGAAAGTCACCGGGGATTATTTTGTGACGCCGATTACATACGGCGACAATAAAGACATTGGAAATCGTAGTACATATT
GGAATACACTGATTATATTAATGATGAATACATACTTTAATATCCTTACGTAGGATCAACATATCTTGTTACAATCGGAC
ACTTTTGCTTCATCCCCGCTAACACCTCTGCACCTTAGACCAAGCGCTTCCACAAGGAACTGAGAGCCATAGCCCACCTC
ACCTTGGGTTCCTTTGGCCGCCTGTCTTTCTGAAAGAGAGCCTTGCCCACCGCAACTATTTCAACACAGATAGGATCAAC
CCGGGATGGCGCTAAGAAGCTATTGCCGCCGATCTTCATAGGGTACCGAGCTCGAATTCAGTACATTAAAAACGTCCGCA
ATGTGTTATTAAGTTGTCTAAGCGTCAATTTGTTTACACCACAATATATCCTGCCACCAGCCAGCCAACAGCTCCCCGAC
CGGCAGCTCGGCACAAAATCACCACTCGATACAGGCAGCCCATCAGTCCGGGACGGCGTCAGCGGGAGAGCCGTTGTAAG
GCGGCAGACTTTGCTCATGTTACCGATGCTATTCGGAAGAACGGCAACTAAGCTGCCGGGTTTGAAACACGGATGATCTC
GCGGAGGGTAGCATGTTGATTGTAACGATGACAGAGCGTTGCTGCCTGTGATCAAATATCATCTCCCTCGCAGAGATCCG
AATTATCAGCCTTCTTATTCATTTCTCGCTTAACCGTGACAGGCTGTCGATCTTGAGAACTATGCCGACATAATAGGAAA
TCGCTGGATAAAGCCGCTGAGGAAGCTGAGTGGCGCTATTTCTTTAGAAGTGAACGTTGACGATCGTCGACGGATCTTTT
CCGCTGCATAACCCTGCTTCGGGGTCATTATAGCGATTTTTTCGGTATATCCATCCTTTTTCGCACGATATACAGGATTT
TGCCAAAGGGTTCGTGTAGACTTTCCTTGGTGTATCCAACGGCGTCAGCCGGGCAGGATAGGTGAAGTAGGCCCACCCGC
GAGCGGGTGTTCCTTCTTCACTGTCCCTTATTCGCACCTGGCGGTGCTCAACGGGAATCCTGCTCTGCGAGGCTGGCCGG
CTACCGCCGGCGTAACAGATGAGGGCAAGCGGATGGCTGATGAAACCAAGCCAACCAGGAAGGGCAGCCCACCTATCAAG
GTGTACTGCCTTCCAGACGAACGAAGAGCGATTGAGGAAAAGGCGGCGGCGGCCGGCATGAGCCTGTCGGCCTACCTGCT
GGCCGTCGGCCAGGGCTACAAAATCACGGGCGTCGTGGACTATGAGCACGTCCGCGAGCTGGCCCGCATCAATGGCGACC
TGGGCCGCCTGGGCGGCCTGCTGAAACTCTGGCTCACCGACGACCCGCGCACGGCGCGGTTCGGTGATGCCACGATCCTC
GCCCTGCTGGCGAAGATCGAAGAGAAGCAGGACGAGCTTGGCAAGGTCATGATGGGCGTGGTCCGCCCGAGGGCAGAGCC
ATGACTTTTTTAGCCGCTAAAACGGCCGGGGGGTGCGCGTGATTGCCAAGCACGTCCCCATGCGCTCCATCAAGAAGAGC
GACTTCGCGGAGCTGGTATTCGTGCAGGGCAAGATTCGGAATACCAAGTACGAGAAGGACGGCCAGACGGTCTACGGGAC
CGACTTCATTGCCGATAAGGTGGATTATCTGGACACCAAGGCACCAGGCGGGTCAAATCAGGAATAAGGGCACATTGCCC
CGGCGTGAGTCGGGGCAATCCCGCAAGGAGGGTGAATGAATCGGACGTTTGACCGGAAGGCATACAGGCAAGAACTGATC
GACGCGGGGTTTTCCGCCGAGGATGCCGAAACCATCGCAAGCCGCACCGTCATGCGTGCGCCCCGCGAAACCTTCCAGTC
CGTCGGCTCGATGGTCCAGCAAGCTACGGCCAAGATCGAGCGCGACAGCGTGCAACTGGCTCCCCCTGCCCTGCCCGCGC
CATCGGCCGCCGTGGAGCGTTCGCGTCGTCTCGAACAGGAGGCGGCAGGTTTGGCGAAGTCGATGACCATCGACACGCGA
GGAACTATGACGACCAAGAAGCGAAAAACCGCCGGCGAGGACCTGGCAAAACAGGTCAGCGAGGCCAAGCAGGCCGCGTT
GCTGAAACACACGAAGCAGCAGATCAAGGAAATGCAGCTTTCCTTGTTCGATATTGCGCCGTGGCCGGACACGATGCGAG
CGATGCCAAACGACACGGCCCGCTCTGCCCTGTTCACCACGCGCAACAAGAAAATCCCGCGCGAGGCGCTGCAAAACAAG
GTCATTTTCCACGTCAACAAGGACGTGAAGATCACCTACACCGGCGTCGAGCTGCGGGCCGACGATGACGAACTGGTGTG
GCAGCAGGTGTTGGAGTACGCGAAGCGCACCCCTATCGGCGAGCCGATCACCTTCACGTTCTACGAGCTTTGCCAGGACC
TGGGCTGGTCGATCAATGGCCGGTATTACACGAAGGCCGAGGAATGCCTGTCGCGCCTACAGGCGACGGCGATGGGCTTC
ACGTCCGACCGCGTTGGGCACCTGGAATCGGTGTCGCTGCTGCACCGCTTCCGCGTCCTGGACCGTGGCAAGAAAACGTC
CCGTTGCCAGGTCCTGATCGACGAGGAAATCGTCGTGCTGTTTGCTGGCGACCACTACACGAAATTCATATGGGAGAAGT
ACCGCAAGCTGTCGCCGACGGCCCGACGGATGTTCGACTATTTCAGCTCGCACCGGGAGCCGTACCCGCTCAAGCTGGAA
ACCTTCCGCCTCATGTGCGGATCGGATTCCACCCGCGTGAAGAAGTGGCGCGAGCAGGTCGGCGAAGCCTGCGAAGAGTT
GCGAGGCAGCGGCCTGGTGGAACACGCCTGGGTCAATGATGACCTGGTGCATTGCAAACGCTAGGGCCTTGTGGGGTCAG
TTCCGGCTGGGGGTTCAGCAGCCAGCGCTTTACTGGCATTTCAGGAACAAGCGGGCACTGCTCGACGCACTTGCTTCGCT
CAGTATCGCTCGGGACGCACGGCGCGCTCTACGAACTGCCGATAAACAGAGGATTAAAATTGACAATTGTGATTAAGGCT
CAGATTCGACGGCTTGGAGCGGCCGACGTGCAGGATTTCCGCGAGATCCGATTGTCGGCCCTGAAGAAAGCTCCAGAGAT
GTTCGGGTCCGTTTACGAGCACGAGGAGAAAAAGCCCATGGAGGCGTTCGCTGAACGGTTGCGAGATGCCGTGGCATTCG
GCGCCTACATCGACGGCGAGATCATTGGGCTGTCGGTCTTCAAACAGGAGGACGGCCCCAAGGACGCTCACAAGGCGCAT
CTGTCCGGCGTTTTCGTGGAGCCCGAACAGCGAGGCCGAGGGGTCGCCGGTATGCTGCTGCGGGCGTTGCCGGCGGGTTT
ATTGCTCGTGATGATCGTCCGACAGATTCCAACGGGAATCTGGTGGATGCGCATCTTCATCCTCGGCGCACTTAATATTT
CGCTATTCTGGAGCTTGTTGTTTATTTCGGTCTACCGCCTGCCGGGCGGGGTCGCGGCGACGGTAGGCGCTGTGCAGCCG
CTGATGGTCGTGTTCATCTCTGCCGCTCTGCTAGGTAGCCCGATACGATTGATGGCGGTCCTGGGGGCTATTTGCGGAAC
TGCGGGCGTGGCGCTGTTGGTGTTGACACCAAACGCAGCGCTAGATCCTGTCGGCGTCGCAGCGGGCCTGGCGGGGGCGG
TTTCCATGGCGTTCGGAACCGTGCTGACCCGCAAGTGGCAACCTCCCGTGCCTCTGCTCACCTTTACCGCCTGGCAACTG
GCGGCCGGAGGACTTCTGCTCGTTCCAGTAGCTTTAGTGTTTGATCCGCCAATCCCGATGCCTACAGGAACCAATGTTCT
CGGCCTGGCGTGGCTCGGCCTGATCGGAGCGGGTTTAACCTACTTCCTTTGGTTCCGGGGGATCTCGCGACTCGAACCTA
CAGTTGTTTCCTTACTGGGCTTTCTCAGCCCGGGGACCGCCGTGTTGCTAGGATGGTTGTTCTTGGATCAGACGCTGAGT
GCGCTTCAAATCATCGGCGTCCTGCTCGTGATCGGGAGTATCTGGCTGGGCCAACGTTCCAACCGCACTCCTAGGGCGCG
TATAGCTTGCCGGAAGTCGCCTTGACCCGCATGGCATAGGCCTATCGTTTCCACGATCAGCGATCGGCTCGTTGCCCTGC
GCCGCTCCAAAGCCCGCGACGCAGCGCCGGCAGGCAGAGCAAGTAGAGGGCAGCGCCTGCAATCCATGCCCACCCGTTCC
ACGTTGTTATAGAAGCCGCATAGATCGCCGTGAAGAGGAGGGGTCCGACGATCGAGGTCAGGCTGGTGAGCGCCGCCAGT
GAGCCTTGCAGCTGCCCCTGACGTTCCTCATCCACCTGCCTGGACAACATTGCTTGCAGCGCCGGCATTCCGATGCCACC
CGAAGCAAGCAGGACCATGATCGGGAACGCCATCCATCCCCGTGTCGCGAAGGCAAGCAGGATGTAGCCTGTGCCGTCGG
CAATCATTCCGAGCATGAGTGCCCGCCTTTCGCCGAGCCGGGCGGCTACAGGGCCGGTGATCATTGCCTGGGCGAGTGAA
TGCAGAATGCCAAATGCGGCAAGCGAAATGCCGATCGTGGTCGCGTCCCAGTGAAAGCGATCCTCGCCGAAAATGACCCA

AAGCGCGGCCGGCACCTGTCCGACAAGTTGCATGATGAAGAAGACCGCCATCAGGGCGGCGACGACGGTCATGCCCCGGG
CCCACCGGAACGAAGCGAGCGGGTTGAGAGCCTCCCGGCGTAACGGCCGGCGTTCGCCTTTGTGCGACTCCGGCAAAAGG
AAACAGCCCGTCAGGAAATTGAGGCCGTTCAAGGCTGCCGCGGCGAAGAACGGAGCGTGGGGGGAGAAACCGCCCATCAG
CCCACCGAGCACAGGTCCCGCGACCATCCCGAACCCGAAACAGGCGCTCATGAAGCCGAAGTGCCGCGCGCGCTCATCGC
CATCAGTGATATCGGCAATATAAGCGCCGGCTACCGCCCCAGTCGCCCCGGTGATGCCGGCCACGATCCGCCCGATATAG
AGAACCCAAAGGAAAGGCGCTGTCGCCATGATGGCGTAGTCGACAGTGGCGCCGGCCAGCGAGACGAGCAAGATTGGCCG
CCGCCCGAAACGATCCGACAGCGCGCCCAGCACAGGTGCGCAGGCAAATTGCACCAACGCATACAGCGCCAGCAGAATGC
CATAGTGGGCGGTGACGTCGTTCGAGTGAACCAGATCGCGCAGGAGGCCCGGCAGCACCGGCATAATCAGGCCGATGCCG
ACAGCGTCGAGCGCGACAGTGCTCAGAATTACGATCAGGGGTATGTTGGGTTTCATGTCTGGCCTCCGGACCAGCCTCCG
CTGGTCCGATTGAACGCGCGGATTCTTTATCACTGATAAGTTGGTGGACATATTATGTTTATCAGTGATAAAGTGTCAAG
CATGACAAAGTTGCAGCCGAATACAGTGATCCGTGCCGCCCTGGACCTGTTGAACGAGGTCGGCGTAGACGGTCTGACGA
CACGCAAACTGGCGGAACGGTTGGGGGTTCAGCAGCCGGCGCTTTACTGGCACTTCAGGAACAAGCGGGCGCTGCTCGAC
GCACTGGCCGAAGCCATGCTGGCGGAGAATCATACGCATTCGGTGCCGAGAGCCGACGACGACTGGCGCTCATTTCTGAT
CGGGAATGCCCGCAGCTTCAGGCAGGCGCTGCTCGCCTACCGCGATGGCGCGCGCATCCATGCCGGCACGCGACCGGGCG
CACCGCAGATGGAAACGGCCGACGCGCAGCTTCGCTTCCTCTGCGAGGCGGGTTTTTCGGCCGGGGACGCCGTCAATGCG
CTGATGACAATCAGCTACTTCACTGTTGGGGCCGTGCTTGAGGAGCAGGCCGGCGACAGCGATGCCGGCGAGCGCGGCGG
CACCGTTGAACAGGCTCCGCTCTCGCCGCTGTTGCGGGCCGCGATAGACGCCTTCGACGAAGCCGGTCCGGACGCAGCGT
TCGAGCAGGGACTCGCGGTGATTGTCGATGGATTGGCGAAAAGGAGGCTCGTTGTCAGGAACGTTGAAGGACCGAGAAAG
GGTGACGATTGATCAGGACCGCTGCCGGAGCGCAACCCACTCACTACAGCAGAGCCATGTAGACAACATCCCCTCCCCCT
TTCCACCGCGTCAGACGCCCGTAGCAGCCCGCTACGGGCTTTTTCATGCCCTGCCCTAGCGTCCAAGCCTCACGGCCGCG
CTCGGCCTCTCTGGCGGCCTTCTGGCGCTCCTGCTGCGGCGTCCGCTCGTGGGCCGTGGCGCGGGTCCGCGCGCCGGCCT
CGTGCGCCTGGCGCTCGCGGGCGAGGTCCAGGGCGGCCGTCTTCACGTTCTGCCTTGCGCAGATGAGATAGATCCAGTCT
GCAAGTGTCAGAAACGAGTCTGCATTTGATGCCCTGAACGTCTTCATACATCTGATAAGCCCCTCCCGGAAGTCTGCTTT
CTCTCCGTCAGAAAGCATACCCTGACCGGTTTCAATCACGGACCGTTCTGTCAGGTAATGCTCTGTCCAGTCATACAGAG
CCGGGTTTGTCACCCGTATAAGCTGAAGCAGGCACAAATCAGGGAAATAAACAAAATCCCGCATCCCCGGATAAAGAAAA
ATCAGGGAATTAATGGCCTGATGGATTTCCCGTGGCGTCGAAAGTGCGGCACCATAGGTATCAGTCACCGCGATGAGATC
CCTTACCATTCCAGAGTCTGGCGGTTGATTATTAATTTGCTGATATAGAGCCTCAGCCCGCTGGCGAAATTCATTACGTA
AATCAAAGGCTTCAGGTCGGGGTAATTTAAAACTAAGCTGAATGATTTTCTGGAGATAACGGCTGCCATCTTCGATATTC
AGCGCATGTTCAACGGCATGAGTGATAATCTGCCTGTCATAACAGAGAATATGGGTAAAGCGGGGCAGATCGGCTACTGC
ACGCACAAGCCTGAACACTTCCGCCACCTGGGATGGCTCCAGTCGGTCCAGATCATCCATGACAACAATGAACTTCAGAT
CCAGACTCACCAGTTGTCCTGCAATTTCAGCCCGAAGCTAGCTTGGCTGCCATTTTTGGGGTGAGGCCGTTCGCGGCCGA
GGGGCGCAGCCCCTGGGGGGATGGGAGGCCCGCGTTAGCGGGCCGGGAGGGTTCGAGAAGGGGGGGCACCCCCCTTCGGC
GTGCGCGGTCACGCGCACAGGGCGCAGCCCTGGTTAAAAACAAGGTTTATAAATATTGGTTTAAAAGCAGGTTAAAAGAC
AGGTTAGCGGTGGCCGAAAAACGGGCGGAAACCCTTGCAAATGCTGGATTTTCTGCCTGTGGACAGCCCCTCAAATGTCA
ATAGGTGCGCCCCTCATCTGTCAGCACTCTGCCCCTCAAGTGTCAAGGATCGCGCCCCTCATCTGTCAGTAGTCGCGCCC
CTCAAGTGTCAATACCGCAGGGCACTTATCCCCAGGCTTGTCCACATCATCTGTGGGAAACTCGCGTAAAATCAGGCGTT
TTCGCCGATTTGCGAGGCTGGCCAGCTCCACGTCGCCGGCCGAAATCGAGCCTGCCCCTCATCTGTCAACGCCGCGCCGG
GTGAGTCGGCCCCTCAAGTGTCAACGTCCGCCCCTCATCTGTCAGTGAGGGCCAAGTTTTCCGCGAGGTATCCACAACGC
CGGCGGCCGCGGTGTCTCGCACACGGCTTCGACGGCGTTTCTGGCGCGTTTGCAGGGCCATAGACGGCCGCCAGCCCAGC
GGCGAGGGCAACCAGCCCGGTGAGCGTCGGAAAGGGTCGACGATCTTGCTGCGTTCGGATATTTTCGTGGAGTTCCCGCC
ACAGACCCGGATTGAAGGCGAGATCCAGCAACTCGCGCCAGATCATCCTGTGACGGAACTTTGGCGCGTGATGACTGGCC
AGGACGTCGGCCGAAAGAGCGACAAGCAGATCACGCTTTTCGACAGCGTCGGATTTGCGATCGAGGATTTTTCGGCGCTG
CGCTACGTCCGCGACCGCGTTGAGGGATCAAGCCACAGCAGCCCACTCGACCTTCTAGCCGACCCAGACGAGCCAAGGGA
TCTTTTTGGAATGCTGCTCCGTCGTCAGGCTTTCCGACGTTTGGGTGGTTGAACAGAAGTCATTATCGCACGGAATGCCA
AGCACTCCCGAGGGGAACCCTGTGGTTGGCATGCACATACAAATGGACGAACGGATAAACCTTTTCACGCCCTTTTAAAT
ATCCGATTATTCTAATAAACGCTCTTTTCTCTTAG

pGAX3 (N. benthamiana FucT_RNAi vector)
The assembled sequence was cloned into XbaI linearized plant expression vector pGA643 to create pGAX3.

FucT sense-intron-antisense RNAi fragment:

TCTAGAGGATCCTTGGCAGCGGCTTTCATTTCTAATTGTGGTGCTCGCAACTTCCGTTTGCAAGCTTTAGAAGCCCTTGA
AAGGGCAAATATCAGAATTGACTCTTATGGAAGTTGTCATCATAACAGGGATGGAAGAGTTGACAAAGTGGCAGCACTGA
AGCGTTACCAGTTTAGCCTGGCTTTTGGGAATTCTAATGAGGAGGACTATGTAACTGAAAAATTCTTTCAGTCTCTGGTA
GCTGGGTCAATCCCTGTGGTGGTTGGTGCTCCAAACATCCAAGACTTTGCGCCTTCTCCTAATTCAGTTTTACACATTAA
AGAGATAAAAGATGCTGAATCAATTGCCAATACCATGAAGTACCTTGCTCAAAACCCTATTGCATATAATGAGTCATTAA
GGTGGAAGTTTGAGGGCCCATCTGATGGATCCactgcacggtatgctcctcttcttgttcatggtcatgatccttatatg
agcagggaaagtccagtttagacttgtagttagttactcttcgttataggatttggatttcttgcgtgtttatggtttta
gtttccctcctttgatgaataaaattgaatcttgtatgagtttcatatccatgttgtgaatctttttgcagacgcagcta
GGTACCGGATCCATCAGATGGGCCCTCAAACTTCCACCTTAATGACTCATTATATGCAATAGGGTTTTGAGCAAGGTACT
TCATGGTATTGGCAATTGATTCAGCATCTTTTATCTCTTTAATGTGTAAAACTGAATTAGGAGAAGGCGCAAAGTCTTGG
ATGTTTGGAGCACCAACCACCACAGGGATTGACCCAGCTACCAGAGACTGAAAGAATTTTTCAGTTACATAGTCCTCCTC
ATTAGAATTCCCAAAAGCCAGGCTAAACTGGTAACGCTTCAGTGCTGCCACTTTGTCAACTCTTCCATCCCTGTTATGAT
GACAACTTCCATAAGAGTCAATTCTGATATTTGCCCTTTCAAGGGCTTCTAAAGCTTGCAAACGGAAGTTGCGAGCACCA
CAATTAGAAATGAAAGCCGCTGCCAATCTAGA

yellow: XbaI site

GTTTACCCGCCAATATATCCTGTCA: RB-DNA repeat

GTTTACACCACAATATATCCTGCCA: LB-DNA repeat

pGAX3 sequence

GTTTACCCGCCAATATATCCTGTCAAACACTGATAGTTTAAACTGAAGGCGGGAAACGACAATCTGATCATGAGCGGAGA
ATTAAGGGAGTCACGTTATGACCCCCGCCGATGACGCGGGACAAGCCGTTTTACGTTTGGAACTGACAGAACCGCAACGT
TGAAGGAGCCACTCAGCCGCGGGTTTCTGGAGTTTAATGAGCTAAGCACATACGTCAGAAACCATTATTGCGCGTTCAAA
AGTCGCCTAAGGTCACTATCAGCTAGCAAATATTTCTTGTCAAAAATGCTCCACTGACGTACCATAAATTCCCCTCGGTA
TCCAATTAGAGTCTCATATTCACTCTCAATCCAAATAATCTGCAATGGCAATTACCTTATCCGCAACTTCTTTACCTATT
TCCGCCCGGATCCGGGCAGGTTCTCCGGCCGCTTGGGTGGAGAGGCTATTCGGCTATGACTGGGCACAACAGACAATCGG
CTGCTCTGATGCCGCCGTGTTCCGGCTGTCAGCGCAGGGGCGCCCGGTTCTTTTTGTCAAGACCGACCTGTCCGGTGCCC
TGAATGAACTGCAGGACGAGGCAGCGCGGCTATCGTGGCTGGCCACGACGGGCGTTCCTTGCGCAGCTGTGCTCGACGTT
GTCACTGAAGCGGGAAGGGACTGGCTGCTATTGGGCGAAGTGCCGGGGCAGGATCTCCTGTCATCTCACCTTGCTCCTGC
CGAGAAAGTATCCATCATGGCTGATGCAATGCGGCGGCTGCATACGCTTGATCCGGCTACCTGCCCATTCGACCACCAAG
CGAAACATCGCATCGAGCGAGCACGTACTCGGATGGAAGCCGGTCTTGTCGATCAGGATGATCTGGACGAAGAGCATCAG
GGGCTCGCGCCAGCCGAACTGTTCGCCAGGCTCAAGGCGCGCATGCCCGACGGCGAGGATCTCGTCGTGACCCATGGCGA
TGCCTGCTTGCCGAATATCATGGTGGAAAATGGCCGCTTTTCTGGATTCATCGACTGTGGCCGGCTGGGTGTGGCGGACC
GCTATCAGGACATAGCGTTGGCTACCCGTGATATTGCTGAAGAGCTTGGCGGCGAATGGGCTGACCGCTTCCTCGTGCTT
TACGGTATCGCCGCTCCCGATTCGCAGCGCATCGCCTTCTATCGCCTTCTTGACGAGTTCTTCTGAGCGGGACTCTGGGG
TTCGAAATGACCGACCAAGCGACGCCCAACCTGCCATCACGAGATTTCGATTCCACCGCCGCCTTCTATGAAAGGTTGGG
CTTCGGAATCGTTTTCCGGGACGCCGGCTGGATGATCCTCCAGCGCGGGGATCTCATGCTGGAGTTCTTCGCCCACCCCG
ATCCAACACTTACGTTTGCAACGTCCAAGAGCAAATAGACCACGAACGCCGGAAGGTTGCCGCAGCGTGTGGATTGCGTC
TCAATTCTCTCTTGCAGGAATGCAATGATGAATATGATACTGACTATGAAACTTTGAGGGAATACTGCCTAGCACCGTCA
CCTCATAACGTGCATCATGCATGCCCTGACAACATGGAACATCGCTATTTTTCTGAAGAATTATGCTCGTTGGAGGATGT
CGCGGCAATTGCAGCTATTGCCAAAATCGAAATACCCCTCACGCATGCATTCATCAATATTATTCATGCGGGGAAAGGCA
AGATTAATCCAACTGGCAAATCATCCAGCGTGATTGGTAACTTCAGTTCCAGCGACTTGATTCGTTTTGGTGCTACCCAC
GTTTTCAATAAGGACGAGATGGTGGAGTAAAGAAGGAGTGCGTCGAAGCAGATCGTTCAAACATTTGGCAATAAAGTTTC
TTAAGATTGAATCCTGTTGCCGGTCTTGCGATGATTATCATATAATTTCTGTTGAATTACGTTAAGCATGTAATAATTAA
CATGTAATGCATGACGTTATTTATGAGATGGGTTTTTATGATTAGAGTCCCGCAATTATACATTTAATACGCGATAGAAA
ACAAAATATAGCGCGCAAACTAGGATAAATTATCGCGCGCGGTGTCATCTATGTTACTAGATCGATCAAACTTCGGTACT
GTGTAATGACGATGAGCAATCGAGAGGCTGACTAACAAAAGGTATGCCCAAAAACAACCTCTCCAAACTGTTTCGAATTG
GAAGTTTCTGCTCATGCCGACAGGCATAACTTAGATATTCGCGGGCTATTCCCACTAATTCGTCCTGCTGGTTTGCGCCA
AGATAAATCAGTGCATCTCCTTACAAGTTCCTCTGTCTTGTGAAATGAACTGCTGACTGCCCCCCAAGAAAGCCTCCTCA
TCTCCCAGTTGGCGGCGGCTGATACACCATCGAAAACCCACGTCCGAACACTTGATACATGTGCCTGAGAAATAGGAACA
TGGTGGAGCACGACACTCTCGTCTACTCCAAGAATATCAAAGATACAGTCTCAGAAGACCAAAGGGCTATTGAGACTTTT
CAACAAAGGGTAATATCGGGAAACCTCCTCGGATTCCATTGCCCAGCTATCTGTCACTTCATCAAAAGGACAGTAGAAAA
GGAAGGTGGCACCTACAAATGCCATCATTGCGATAAAGGAAAGGCTATCGTTCAAGATGCCTCTGCCGACAGTGGTCCCA
AAGATGGACCCCCACCCACGAGGAGCATCGTGGAAAAAGAAGACGTTCCAACCACGTCTTCAAAGCAAGTGGATTGATGT

GATATCTCCACTGACGTAAGGGATGACGCACAATCCCACTATCCTTCGCAAGACCCTTCCTCTATATAAGGAAGTTCATT
TCATTTGGAGAGGACCCTCGACCAAGCTTCTAGAGGATCCTTGGCAGCGGCTTTCATTTCTAATTGTGGTGCTCGCAACT
TCCGTTTGCAAGCTTTAGAAGCCCTTGAAAGGGCAAATATCAGAATTGACTCTTATGGAAGTTGTCATCATAACAGGGAT
GGAAGAGTTGACAAAGTGGCAGCACTGAAGCGTTACCAGTTTAGCCTGGCTTTTGGGAATTCTAATGAGGAGGACTATGT
AACTGAAAAATTCTTTCAGTCTCTGGTAGCTGGGTCAATCCCTGTGGTGGTTGGTGCTCCAAACATCCAAGACTTTGCGC
CTTCTCCTAATTCAGTTTTACACATTAAAGAGATAAAAGATGCTGAATCAATTGCCAATACCATGAAGTACCTTGCTCAA
AACCCTATTGCATATAATGAGTCATTAAGGTGGAAGTTTGAGGGCCCATCTGATGGATCCactgcacggtatgctcctct
tcttgttcatggtcatgatccttatatgagcagggaaagtccagtttagacttgtagttagttactcttcgttataggat
ttggatttcttgcgtgtttatggttttagtttccctcctttgatgaataaaattgaatcttgtatgagtttcatatccat
gttgtgaatctttttgcagacgcagctaGGTACCGGATCCATCAGATGGGCCCTCAAACTTCCACCTTAATGACTCATTA
TATGCAATAGGGTTTTGAGCAAGGTACTTCATGGTATTGGCAATTGATTCAGCATCTTTTATCTCTTTAATGTGTAAAAC
TGAATTAGGAGAAGGCGCAAAGTCTTGGATGTTTGGAGCACCAACCACCACAGGGATTGACCCAGCTACCAGAGACTGAA
AGAATTTTTCAGTTACATAGTCCTCCTCATTAGAATTCCCAAAAGCCAGGCTAAACTGGTAACGCTTCAGTGCTGCCACT
TTGTCAACTCTTCCATCCCTGTTATGATGACAACTTCCATAAGAGTCAATTCTGATATTTGCCCTTTCAAGGGCTTCTAA
AGCTTGCAAACGGAAGTTGCGAGCACCACAATTAGAAATGAAAGCCGCTGCCAATCTAGAGCTCGTTAACGGTACCATCG
ATAGATCTGCGATGAGCTAAGCTAGCTATATCATCAATTTATGTATTACACATAATATCGCACTCAGTCTTTCATCTACG
GCAATGTACCAGCTGATATAATCAGTTATTGAAATATTTCTGAATTTAAACTTGCATCAATAAATTTATGTTTTTGCTTG
GACTATAATACCTGACTTGTTATTTTATCAATAAATATTTAAACTATATTTCTTTCAAGATATCATTCTTTACAAGTATA
CGTGTTTAAATTGAATACCATAAATTTTTATTTTTCAAATACATGTAAAATTATGAAATGGGAGTGGTGGCGACCGAGCT
CAAGCACACTTCAATTCCTATAACGGACCAAATCGCAAAAATTATAATAACATATTATTTCATCCTGGATTAAAAGAAAG
TCACCGGGGATTATTTTGTGACGCCGATTACATACGGCGACAATAAAGACATTGGAAATCGTAGTACATATTGGAATACA
CTGATTATATTAATGATGAATACATACTTTAATATCCTTACGTAGGATCAACATATCTTGTTACAATCGGACACTTTTGC
TTCATCCCCGCTAACACCTCTGCACCTTAGACCAAGCGCTTCCACAAGGAACTGAGAGCCATAGCCCACCTCACCTTGGG
TTCCTTTGGCCGCCTGTCTTTCTGAAAGAGAGCCTTGCCCACCGCAACTATTTCAACACAGATAGGATCAACCCGGGATG
GCGCTAAGAAGCTATTGCCGCCGATCTTCATAGGGTACCGAGCTCGAATTCAGTACATTAAAAACGTCCGCAATGTGTTA
TTAAGTTGTCTAAGCGTCAATTTGTTTACACCACAATATATCCTGCCACCAGCCAGCCAACAGCTCCCCGACCGGCAGCT
CGGCACAAAATCACCACTCGATACAGGCAGCCCATCAGTCCGGGACGGCGTCAGCGGGAGAGCCGTTGTAAGGCGGCAGA
CTTTGCTCATGTTACCGATGCTATTCGGAAGAACGGCAACTAAGCTGCCGGGTTTGAAACACGGATGATCTCGCGGAGGG
TAGCATGTTGATTGTAACGATGACAGAGCGTTGCTGCCTGTGATCAAATATCATCTCCCTCGCAGAGATCCGAATTATCA
GCCTTCTTATTCATTTCTCGCTTAACCGTGACAGGCTGTCGATCTTGAGAACTATGCCGACATAATAGGAAATCGCTGGA
TAAAGCCGCTGAGGAAGCTGAGTGGCGCTATTTCTTTAGAAGTGAACGTTGACGATCGTCGACGGATCTTTTCCGCTGCA
TAACCCTGCTTCGGGGTCATTATAGCGATTTTTTCGGTATATCCATCCTTTTTCGCACGATATACAGGATTTTGCCAAAG
GGTTCGTGTAGACTTTCCTTGGTGTATCCAACGGCGTCAGCCGGGCAGGATAGGTGAAGTAGGCCCACCCGCGAGCGGGT
GTTCCTTCTTCACTGTCCCTTATTCGCACCTGGCGGTGCTCAACGGGAATCCTGCTCTGCGAGGCTGGCCGGCTACCGCC
GGCGTAACAGATGAGGGCAAGCGGATGGCTGATGAAACCAAGCCAACCAGGAAGGGCAGCCCACCTATCAAGGTGTACTG
CCTTCCAGACGAACGAAGAGCGATTGAGGAAAAGGCGGCGGCGGCCGGCATGAGCCTGTCGGCCTACCTGCTGGCCGTCG
GCCAGGGCTACAAAATCACGGGCGTCGTGGACTATGAGCACGTCCGCGAGCTGGCCCGCATCAATGGCGACCTGGGCCGC
CTGGGCGGCCTGCTGAAACTCTGGCTCACCGACGACCCGCGCACGGCGCGGTTCGGTGATGCCACGATCCTCGCCCTGCT
GGCGAAGATCGAAGAGAAGCAGGACGAGCTTGGCAAGGTCATGATGGGCGTGGTCCGCCCGAGGGCAGAGCCATGACTTT
TTTAGCCGCTAAAACGGCCGGGGGGTGCGCGTGATTGCCAAGCACGTCCCCATGCGCTCCATCAAGAAGAGCGACTTCGC
GGAGCTGGTATTCGTGCAGGGCAAGATTCGGAATACCAAGTACGAGAAGGACGGCCAGACGGTCTACGGGACCGACTTCA
TTGCCGATAAGGTGGATTATCTGGACACCAAGGCACCAGGCGGGTCAAATCAGGAATAAGGGCACATTGCCCCGGCGTGA
GTCGGGGCAATCCCGCAAGGAGGGTGAATGAATCGGACGTTTGACCGGAAGGCATACAGGCAAGAACTGATCGACGCGGG
GTTTTCCGCCGAGGATGCCGAAACCATCGCAAGCCGCACCGTCATGCGTGCGCCCCGCGAAACCTTCCAGTCCGTCGGCT
CGATGGTCCAGCAAGCTACGGCCAAGATCGAGCGCGACAGCGTGCAACTGGCTCCCCCTGCCCTGCCCGCGCCATCGGCC
GCCGTGGAGCGTTCGCGTCGTCTCGAACAGGAGGCGGCAGGTTTGGCGAAGTCGATGACCATCGACACGCGAGGAACTAT
GACGACCAAGAAGCGAAAAACCGCCGGCGAGGACCTGGCAAAACAGGTCAGCGAGGCCAAGCAGGCCGCGTTGCTGAAAC
ACACGAAGCAGCAGATCAAGGAAATGCAGCTTTCCTTGTTCGATATTGCGCCGTGGCCGGACACGATGCGAGCGATGCCA
AACGACACGGCCCGCTCTGCCCTGTTCACCACGCGCAACAAGAAAATCCCGCGCGAGGCGCTGCAAAACAAGGTCATTTT
CCACGTCAACAAGGACGTGAAGATCACCTACACCGGCGTCGAGCTGCGGGCCGACGATGACGAACTGGTGTGGCAGCAGG
TGTTGGAGTACGCGAAGCGCACCCCTATCGGCGAGCCGATCACCTTCACGTTCTACGAGCTTTGCCAGGACCTGGGCTGG
TCGATCAATGGCCGGTATTACACGAAGGCCGAGGAATGCCTGTCGCGCCTACAGGCGACGGCGATGGGCTTCACGTCCGA
CCGCGTTGGGCACCTGGAATCGGTGTCGCTGCTGCACCGCTTCCGCGTCCTGGACCGTGGCAAGAAAACGTCCCGTTGCC
AGGTCCTGATCGACGAGGAAATCGTCGTGCTGTTTGCTGGCGACCACTACACGAAATTCATATGGGAGAAGTACCGCAAG
CTGTCGCCGACGGCCCGACGGATGTTCGACTATTTCAGCTCGCACCGGGAGCCGTACCCGCTCAAGCTGGAAACCTTCCG
CCTCATGTGCGGATCGGATTCCACCCGCGTGAAGAAGTGGCGCGAGCAGGTCGGCGAAGCCTGCGAAGAGTTGCGAGGCA
GCGGCCTGGTGGAACACGCCTGGGTCAATGATGACCTGGTGCATTGCAAACGCTAGGGCCTTGTGGGGTCAGTTCCGGCT
GGGGGTTCAGCAGCCAGCGCTTTACTGGCATTTCAGGAACAAGCGGGCACTGCTCGACGCACTTGCTTCGCTCAGTATCG
CTCGGGACGCACGGCGCGCTCTACGAACTGCCGATAAACAGAGGATTAAAATTGACAATTGTGATTAAGGCTCAGATTCG
ACGGCTTGGAGCGGCCGACGTGCAGGATTTCCGCGAGATCCGATTGTCGGCCCTGAAGAAAGCTCCAGAGATGTTCGGGT
CCGTTTACGAGCACGAGGAGAAAAAGCCCATGGAGGCGTTCGCTGAACGGTTGCGAGATGCCGTGGCATTCGGCGCCTAC
ATCGACGGCGAGATCATTGGGCTGTCGGTCTTCAAACAGGAGGACGGCCCCAAGGACGCTCACAAGGCGCATCTGTCCGG
CGTTTTCGTGGAGCCCGAACAGCGAGGCCGAGGGGTCGCCGGTATGCTGCTGCGGGCGTTGCCGGCGGGTTTATTGCTCG
TGATGATCGTCCGACAGATTCCAACGGGAATCTGGTGGATGCGCATCTTCATCCTCGGCGCACTTAATATTTCGCTATTC
TGGAGCTTGTTGTTTATTTCGGTCTACCGCCTGCCGGGCGGGGTCGCGGCGACGGTAGGCGCTGTGCAGCCGCTGATGGT
CGTGTTCATCTCTGCCGCTCTGCTAGGTAGCCCGATACGATTGATGGCGGTCCTGGGGGCTATTTGCGGAACTGCGGGCG
TGGCGCTGTTGGTGTTGACACCAAACGCAGCGCTAGATCCTGTCGGCGTCGCAGCGGGCCTGGCGGGGGCGGTTTCCATG
GCGTTCGGAACCGTGCTGACCCGCAAGTGGCAACCTCCCGTGCCTCTGCTCACCTTTACCGCCTGGCAACTGGCGGCCGG
AGGACTTCTGCTCGTTCCAGTAGCTTTAGTGTTTGATCCGCCAATCCCGATGCCTACAGGAACCAATGTTCTCGGCCTGG
CGTGGCTCGGCCTGATCGGAGCGGGTTTAACCTACTTCCTTTGGTTCCGGGGGATCTCGCGACTCGAACCTACAGTTGTT
TCCTTACTGGGCTTTCTCAGCCCGGGGACCGCCGTGTTGCTAGGATGGTTGTTCTTGGATCAGACGCTGAGTGCGCTTCA

AATCATCGGCGTCCTGCTCGTGATCGGGAGTATCTGGCTGGGCCAACGTTCCAACCGCACTCCTAGGGCGCGTATAGCTT
GCCGGAAGTCGCCTTGACCCGCATGGCATAGGCCTATCGTTTCCACGATCAGCGATCGGCTCGTTGCCCTGCGCCGCTCC
AAAGCCCGCGACGCAGCGCCGGCAGGCAGAGCAAGTAGAGGGCAGCGCCTGCAATCCATGCCCACCCGTTCCACGTTGTT
ATAGAAGCCGCATAGATCGCCGTGAAGAGGAGGGGTCCGACGATCGAGGTCAGGCTGGTGAGCGCCGCCAGTGAGCCTTG
CAGCTGCCCCTGACGTTCCTCATCCACCTGCCTGGACAACATTGCTTGCAGCGCCGGCATTCCGATGCCACCCGAAGCAA
GCAGGACCATGATCGGGAACGCCATCCATCCCCGTGTCGCGAAGGCAAGCAGGATGTAGCCTGTGCCGTCGGCAATCATT
CCGAGCATGAGTGCCCGCCTTTCGCCGAGCCGGGCGGCTACAGGGCCGGTGATCATTGCCTGGGCGAGTGAATGCAGAAT
GCCAAATGCGGCAAGCGAAATGCCGATCGTGGTCGCGTCCCAGTGAAAGCGATCCTCGCCGAAAATGACCCAAAGCGCGG
CCGGCACCTGTCCGACAAGTTGCATGATGAAGAAGACCGCCATCAGGGCGGCGACGACGGTCATGCCCCGGGCCCACCGG
AACGAAGCGAGCGGGTTGAGAGCCTCCCGGCGTAACGGCCGGCGTTCGCCTTTGTGCGACTCCGGCAAAAGGAAACAGCC
CGTCAGGAAATTGAGGCCGTTCAAGGCTGCCGCGGCGAAGAACGGAGCGTGGGGGGAGAAACCGCCCATCAGCCCACCGA
GCACAGGTCCCGCGACCATCCCGAACCCGAAACAGGCGCTCATGAAGCCGAAGTGCCGCGCGCGCTCATCGCCATCAGTG
ATATCGGCAATATAAGCGCCGGCTACCGCCCCAGTCGCCCCGGTGATGCCGGCCACGATCCGCCCGATATAGAGAACCCA
AAGGAAAGGCGCTGTCGCCATGATGGCGTAGTCGACAGTGGCGCCGGCCAGCGAGACGAGCAAGATTGGCCGCCGCCCGA
AACGATCCGACAGCGCGCCCAGCACAGGTGCGCAGGCAAATTGCACCAACGCATACAGCGCCAGCAGAATGCCATAGTGG
GCGGTGACGTCGTTCGAGTGAACCAGATCGCGCAGGAGGCCCGGCAGCACCGGCATAATCAGGCCGATGCCGACAGCGTC
GAGCGCGACAGTGCTCAGAATTACGATCAGGGGTATGTTGGGTTTCATGTCTGGCCTCCGGACCAGCCTCCGCTGGTCCG
ATTGAACGCGCGGATTCTTTATCACTGATAAGTTGGTGGACATATTATGTTTATCAGTGATAAAGTGTCAAGCATGACAA
AGTTGCAGCCGAATACAGTGATCCGTGCCGCCCTGGACCTGTTGAACGAGGTCGGCGTAGACGGTCTGACGACACGCAAA
CTGGCGGAACGGTTGGGGGTTCAGCAGCCGGCGCTTTACTGGCACTTCAGGAACAAGCGGGCGCTGCTCGACGCACTGGC
CGAAGCCATGCTGGCGGAGAATCATACGCATTCGGTGCCGAGAGCCGACGACGACTGGCGCTCATTTCTGATCGGGAATG
CCCGCAGCTTCAGGCAGGCGCTGCTCGCCTACCGCGATGGCGCGCGCATCCATGCCGGCACGCGACCGGGCGCACCGCAG
ATGGAAACGGCCGACGCGCAGCTTCGCTTCCTCTGCGAGGCGGGTTTTTCGGCCGGGGACGCCGTCAATGCGCTGATGAC
AATCAGCTACTTCACTGTTGGGGCCGTGCTTGAGGAGCAGGCCGGCGACAGCGATGCCGGCGAGCGCGGCGGCACCGTTG
AACAGGCTCCGCTCTCGCCGCTGTTGCGGGCCGCGATAGACGCCTTCGACGAAGCCGGTCCGGACGCAGCGTTCGAGCAG
GGACTCGCGGTGATTGTCGATGGATTGGCGAAAAGGAGGCTCGTTGTCAGGAACGTTGAAGGACCGAGAAAGGGTGACGA
TTGATCAGGACCGCTGCCGGAGCGCAACCCACTCACTACAGCAGAGCCATGTAGACAACATCCCCTCCCCCTTTCCACCG
CGTCAGACGCCCGTAGCAGCCCGCTACGGGCTTTTTCATGCCCTGCCCTAGCGTCCAAGCCTCACGGCCGCGCTCGGCCT
CTCTGGCGGCCTTCTGGCGCTCCTGCTGCGGCGTCCGCTCGTGGGCCGTGGCGCGGGTCCGCGCGCCGGCCTCGTGCGCC
TGGCGCTCGCGGGCGAGGTCCAGGGCGGCCGTCTTCACGTTCTGCCTTGCGCAGATGAGATAGATCCAGTCTGCAAGTGT
CAGAAACGAGTCTGCATTTGATGCCCTGAACGTCTTCATACATCTGATAAGCCCCTCCCGGAAGTCTGCTTTCTCTCCGT
CAGAAAGCATACCCTGACCGGTTTCAATCACGGACCGTTCTGTCAGGTAATGCTCTGTCCAGTCATACAGAGCCGGGTTT
GTCACCCGTATAAGCTGAAGCAGGCACAAATCAGGGAAATAAACAAAATCCCGCATCCCCGGATAAAGAAAAATCAGGGA
ATTAATGGCCTGATGGATTTCCCGTGGCGTCGAAAGTGCGGCACCATAGGTATCAGTCACCGCGATGAGATCCCTTACCA
TTCCAGAGTCTGGCGGTTGATTATTAATTTGCTGATATAGAGCCTCAGCCCGCTGGCGAAATTCATTACGTAAATCAAAG
GCTTCAGGTCGGGGTAATTTAAAACTAAGCTGAATGATTTTCTGGAGATAACGGCTGCCATCTTCGATATTCAGCGCATG
TTCAACGGCATGAGTGATAATCTGCCTGTCATAACAGAGAATATGGGTAAAGCGGGGCAGATCGGCTACTGCACGCACAA
GCCTGAACACTTCCGCCACCTGGGATGGCTCCAGTCGGTCCAGATCATCCATGACAACAATGAACTTCAGATCCAGACTC
ACCAGTTGTCCTGCAATTTCAGCCCGAAGCTAGCTTGGCTGCCATTTTTGGGGTGAGGCCGTTCGCGGCCGAGGGGCGCA
GCCCCTGGGGGGATGGGAGGCCCGCGTTAGCGGGCCGGGAGGGTTCGAGAAGGGGGGGCACCCCCCTTCGGCGTGCGCGG
TCACGCGCACAGGGCGCAGCCCTGGTTAAAAACAAGGTTTATAAATATTGGTTTAAAAGCAGGTTAAAAGACAGGTTAGC
GGTGGCCGAAAAACGGGCGGAAACCCTTGCAAATGCTGGATTTTCTGCCTGTGGACAGCCCCTCAAATGTCAATAGGTGC
GCCCCTCATCTGTCAGCACTCTGCCCCTCAAGTGTCAAGGATCGCGCCCCTCATCTGTCAGTAGTCGCGCCCCTCAAGTG
TCAATACCGCAGGGCACTTATCCCCAGGCTTGTCCACATCATCTGTGGGAAACTCGCGTAAAATCAGGCGTTTTCGCCGA
TTTGCGAGGCTGGCCAGCTCCACGTCGCCGGCCGAAATCGAGCCTGCCCCTCATCTGTCAACGCCGCGCCGGGTGAGTCG
GCCCCTCAAGTGTCAACGTCCGCCCCTCATCTGTCAGTGAGGGCCAAGTTTTCCGCGAGGTATCCACAACGCCGGCGGCC
GCGGTGTCTCGCACACGGCTTCGACGGCGTTTCTGGCGCGTTTGCAGGGCCATAGACGGCCGCCAGCCCAGCGGCGAGGG
CAACCAGCCCGGTGAGCGTCGGAAAGGGTCGACGATCTTGCTGCGTTCGGATATTTTCGTGGAGTTCCCGCCACAGACCC
GGATTGAAGGCGAGATCCAGCAACTCGCGCCAGATCATCCTGTGACGGAACTTTGGCGCGTGATGACTGGCCAGGACGTC
GGCCGAAAGAGCGACAAGCAGATCACGCTTTTCGACAGCGTCGGATTTGCGATCGAGGATTTTTCGGCGCTGCGCTACGT
CCGCGACCGCGTTGAGGGATCAAGCCACAGCAGCCCACTCGACCTTCTAGCCGACCCAGACGAGCCAAGGGATCTTTTTG
GAATGCTGCTCCGTCGTCAGGCTTTCCGACGTTTGGGTGGTTGAACAGAAGTCATTATCGCACGGAATGCCAAGCACTCC
CGAGGGGAACCCTGTGGTTGGCATGCACATACAAATGGACGAACGGATAAACCTTTTCACGCCCTTTTAAATATCCGATT
ATTCTAATAAACGCTCTTTTCTCTTAG
